# One-Pot Synthesis of 1,3,4-Oxadiazines from Acylhydrazides and Allenoates

**DOI:** 10.3390/molecules28093815

**Published:** 2023-04-29

**Authors:** Su Been Kim, Santanu Maiti, Eun Sun Park, Ga Young Kim, Yunji Choun, Soon Kil Ahn, Jae Kwang Kim, Jinho Kim

**Affiliations:** 1 Department of Chemistry and Research Institute of Basic Sciences, Incheon National University, Incheon 22012, Republic of Korea; ksb9433@naver.com (S.B.K.); santanumaiti.chem@gmail.com (S.M.); qkrdmstjs228@gmail.com (E.S.P.); kyy07020@naver.com (G.Y.K.); choun0817@naver.com (Y.C.); 2 Institute for New Drug Development, Division of Life Sciences, Incheon National University, Incheon 22012, Republic of Korea; skahn@inu.ac.kr; 3 Division of Life Sciences, Incheon National University, Incheon 22012, Republic of Korea; kjkpj@inu.ac.kr

**Keywords:** 1,3,4-oxadiazines, acylhydrazides, allenoates, aerobic oxidation, green synthesis

## Abstract

The framework of 1,3,4-oxadiazine is crucial for numerous bioactive molecules, but only a limited number of synthetic methods have been reported for its production. In 2015, Wang’s group developed a 4-(dimethylamino)pyridine (DMAP)-catalyzed [2 + 4] cycloaddition of allenoates with *N*-acyldiazenes, which provided an atom-efficient route for 1,3,4-oxadiazines. However, the practicality of this method was limited by the instability of *N*-acyldiazenes as starting materials. Building upon our ongoing research about the aerobic oxidation of hydrazides and their synthetic applications, we hypothesized that aerobic oxidative cycloadditions using acylhydrazides instead of *N*-acyldiazenes may provide a more practical synthetic route for 1,3,4-oxadiazines. In this manuscript, we describe a one-pot synthetic protocol for 1,3,4-oxadiazines from acylhydrazides and allenoates. The developed one-pot protocol consists of aerobic oxidations of acylhydrazides into *N*-acyldiazenes using NaNO_2_ and HNO_3_, followed by the DMAP-catalyzed cycloaddition of allenoate with the generated *N*-acyldiazenes. A variety of 1,3,4-oxadiazines were produced in good to high yields. In addition, the practicality of the developed method was demonstrated by a gram-scale synthesis of 1,3,4-oxadiazine.

## 1. Introduction

Azo compounds are one of the most versatile organic molecules in organic synthesis [1,2,3,4,5]. Azo compounds such as diethyl azodicarboxylate (DEAD) play a crucial role to facilitate the stereoselective conversion of alcohols in the Mitsunobu reaction [6,7,8,9,10]. In addition, the azo compounds can be employed as oxidants in dehydrogenations [11,12,13,14,15,16] as well as cross dehydrogenative couplings [17,18,19,20,21,22]. It was also found that azo compounds can serve as a directing group for C-H activation [23].

Among various azo compounds, *N*-acyldiazenes have recently emerged as attractive participants in cycloadditions for the construction of biologically important *N*-heterocycles [24]. A variety of synthetic methods for pyrazolidinones [25], 1,3,4-oxadiazin-6-ones [26,27,28,29,30], Blatter radicals [31], 1,3,4-oxadiazines [32,33], 1,3,4-oxadiazoles [34], isatin derivatives [35], 3-spiropyrazole-2-oxindoles [36], and dihydrobenzo[*e*]indoles [37] have been developed through cycloadditions between the *N*-acyldiazenes and corresponding partners.

**Scheme 1:**
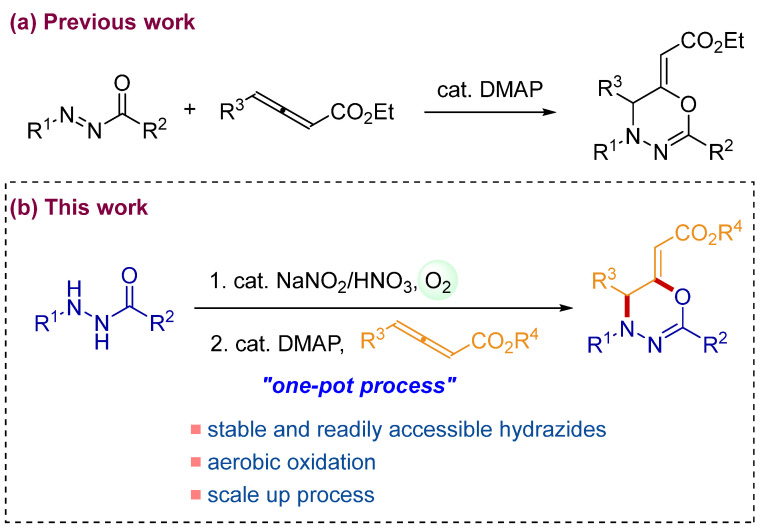
Synthesis of 1,3,4-oxadiazines from allenoates. (**a**) Previously reported DMAP-catalyzed cycloaddition of allenoates with *N*-acyldiazenes. (**b**) Our developed one-pot synthesis of 1,3,4-oxadiazine from acylhydrazides and allenoates.

In 2015, Wang’s group developed an atom-efficient synthetic route for 1,3,4-oxadiazines using a [2 + 4] cycloaddition of allenoates with *N*-acyldiazenes catalyzed by 4-(dimethylamino)pyridine (DMAP) (Scheme 1a) [38]. Subsequently, Li and coworkers reported that the asymmetric synthesis of 1,3,4-oxadiazines from allenoates and *N*-acyldiazenes was achieved by chiral DMAP analogs as catalysts (70–87% enantiomeric excess) [39]. While these methods are promising for the synthesis of biologically important 1,3,4-oxadiazines, the use of less-stable *N*-acyldiazenes as starting materials requires laborious preparation and careful storage. Therefore, the development of simpler and more practical protocols for 1,3,4-oxadiazines using stable precursors of azo compounds is highly desirable.

Our group has investigated the aerobic oxidations of acylhydrazides to *N*-acyldiazenes [40,41,42] and their synthetic applications in organic transformations [43,44]. Building upon our studies in this research area, we envisioned that aerobic oxidative cycloaddition using acylhydrazides instead of *N*-acyldiazenes may provide more practical and green synthetic routes to 1,3,4-oxadiazines [45,46,47,48,49]. Because the acylhydrazides are relatively more stable than *N*-acyldiazenes, and only water is produced during the aerobic oxidations of the acylhydrazides, the laborious preparations and purifications of less-stable *N*-acyldiazenes are not required in our envisaged strategy. Herein, we report a one-pot route to 1,3,4-oxadiazines through the aerobic oxidation of acylhydrazides and the DMAP-catalyzed cycloaddition of the in situ generated *N*-acyldiazenes with allenoates (Scheme 1b).

## 2. Results

We initially examined the direct aerobic oxidative cyclization of *N′*-phenylbenzohydrazide (**1a**) and benzyl allenoate (**2a**) using the CuCl/DMAP system (Table 1, entry 1) [40]. We presumed that the used DMAP facilitates not only the aerobic oxidation of acylhydrazide to *N*-acyldiazene but also cyclization between the generated azo compound and allenoate; however, only 14% of 1,3,4-oxadiazine **3a** was produced. The use of the NO*_x_* catalytic system (NaNO_2_ and HNO_3_) with DMAP showed a promising result [41] compared to other catalytic systems such as Fe(Pc) and Mn(Pc) (Table 1, entries 2–4) [44,50]. Despite implementing several optimizations in the direct aerobic oxidative cyclization approach, however, there was no notable improvement in the yield of **3a**.

Next, we tested the feasibility of a one-pot synthesis of **3a** from **1a** and **2a** (Table 2). Full conversions of **1a** to benzoyl-2-phenyldiazene **4a** were observed in the aerobic oxidations catalyzed by the previously reported catalytic systems such as CuCl/DMAP, Fe(Pc), and Mn(Pc) in 2 h; however, the following DAMP-catalyzed cycloadditions between **2a** and the generated **4a** were less reactive (entries 1–3). We assumed that the remaining metal catalysts probably hampered the desired DMAP-catalyzed cycloaddition. Interestingly, it was found that the one-pot sequential protocol consisting of aerobic oxidation using the NO*_x_* catalytic system followed by DMAP-catalyzed cycloaddition produced the desired **3a** with a good yield (entry 4). This result indicated that the used NaNO_2_/HNO_3_ reagents and the byproducts formed during aerobic oxidation are compatible with the second step, DMAP-catalyzed cyclization of the generated **4a** and **2a**. With the NO*_x_* catalytic system, other bases and solvents were screened. The use of pyridine as a base showed an inferior result to DMAP (entry 5), and no reaction was observed when 1,8-diazabicyclo [5.4.0] undec-7-ene (DBU) was employed as a base (entry 6). Various solvents, such as CH_3_CN, CH_2_Cl_2_, and 1,4-dioxane, were tested as reaction media, but they resulted in lower yields compared to toluene (entries 7–9). The one-pot synthesis in an eco-friendly solvent such as EtOH was sluggish (entry 10). We aimed to minimize the amounts of DAMP and **2a** and determined that the use of 30 mol % of DMAP and 1.2 equivalents of **2a** was sufficient to facilitate the developed one-pot 1,3,4-oxadiazine synthesis (entry 11).

**Figure 1:**
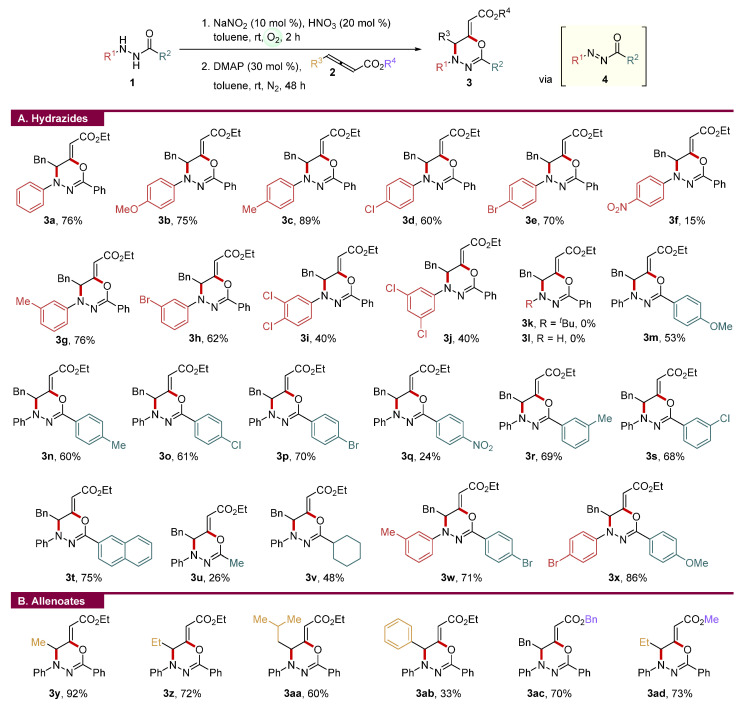
Substrate scope of acylhydrazides and allenoates *^a^*^,*b*^. *^a^* Reaction conditions: **1** (0.3 mmol), NaNO_2_ (10 mol %), and HNO_3_ (20 mol %) in toluene (1.5 mL) under O_2_ at room temperature for 2 h were used for the first step. The reaction mixture of the first step was added to DMAP (30 mol %) and **2** (0.36 mmol) in toluene (1.5 mL) under N_2_ at room temperature; then, the reaction mixture of the second step was stirred for 48 h. *^b^* Isolated yield.

The optimized one-pot reaction conditions were then tested with various acylhydrazides to gain insight into the substrate scope, and the results are described in Figure 1A. First, the reactivities of acylhydrazides bearing the para-substituted phenyl group at the R^1^ position were studied. Electron-rich acylhydrazides or halogen-substituted acylhydrazides produced the corresponding 1,3,4-oxadiazines in good to high yields (**3b**–**3e**). The present one-pot protocol was successful in preparing 4-nitro phenyl substituted 1,3,4-oxadiazine **3f**, which was not accessible using the previous method [38], albeit in a low yield. Both meta-substituted phenyl hydrazides and di-substituted phenyl hydrazides were successfully employed for the synthesis of 1,3,4-oxadiazines (**3g**–**3j**). However, several problematic substrates were observed in the developed one-pot protocol. For example, aliphatic acylhydrazides such as *N′*-propybenzohydrazide, *N′*-isopropylbenzohydrazide, and *N*′-*tert*-butylbenzohydrazide did not produce the desired 1,3,4-oxadiazine in spite of the full conversion to the corresponding *N*-acyldiazenes (**3k**) [51]. Non-substituted benzoyl hydrazide was also tested, but the desired 1,3,4-oxadiazines could not be accessible, presumably due to decomposition of the unstable azo intermediate (**3l**).

Following that, the substrate scope of the R^2^ position was investigated. In general, various para- or meta-substituted benzoyl hydrazides smoothly underwent the developed one-pot protocol regardless of the electronic environments (**3m**–**3s**). The 1,3,4-oxadiazine with a naphthalene moiety was synthesized with 75% yield through the developed one-pot protocol (**3t**). It is noteworthy that the synthesis of 1,3,4-oxadiazines with aliphatic groups at the R^2^ position could be synthesized by the present one-pot method, producing **3u** with 26% yield and **3v** with 48% yield. Other multi-substituted 1,3,4-oxadiazines could be prepared with good to high yields (**3w** and **3x**).

The substrate scope of the γ-substituted allenoates was also investigated (Figure 1B) [52]. In addition to the benzyl substituent at γ-position, other aliphatic substituents such as methyl, ethyl, and isobutyl were successfully employed, and the corresponding 1,3,4-oxadiazines were produced with good yields (**3y**–**3aa**). The one-pot cyclization using the allenoate-bearing phenyl group at the γ-position had a poor yield in spite of the full conversion of allenoate (**3ab**). It was revealed that the present one-pot protocol was not significantly influenced by the ester group of allenoate (**3ac** and **3ad**).

**Scheme 2:**
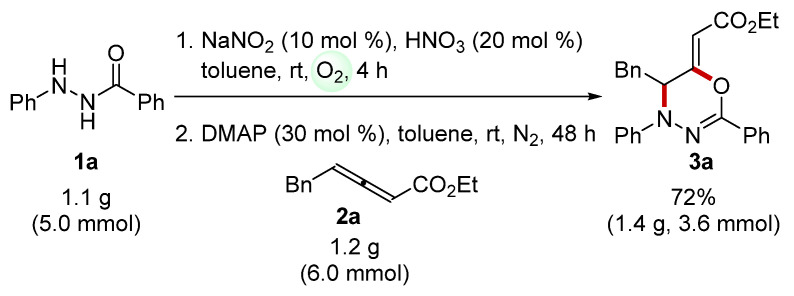
Scaled-up process for the one-pot synthesis of **3a**.

In order to showcase the practicality and efficiency of the present one-pot protocol, we carried out a gram-scale reaction using **1a** (1.1 g, 5.0 mmol) and **2a** (1.2 g, 6.0 mmol) under slightly modified conditions (Scheme 2). The desired 1,3,4-oxadiazine **3a** was obtained with 72% yield (1.4 g, 3.6 mmol) without a significant decrease in reactivity. The previous reports only achieved small-scale (0.2 mmol) synthesis of 1,3,4-oxadiazines, possibly because of the challenging isolation and purification of *N*-acyldiazenes. Therefore, our developed one-pot method for 1,3,4-oxadiazine from acylhydrazides offers a practical and efficient approach for the synthesis of 1,3,4-oxadiazines on a larger scale.

**Figure 2:**
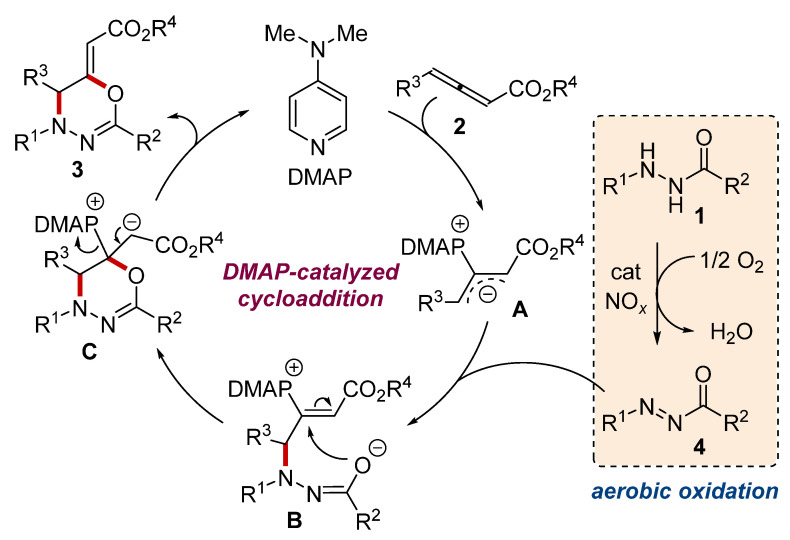
Proposed mechanism of the developed one-pot protocol.

The proposed mechanism of the developed one-pot synthesis 1,3,4-oxadiazines is depicted in Figure 2. Initially, the allenoate **2** was activated by DMAP and zwitterionic intermediate **A** was generated. The conjugate addition of the generated **A** to *N*-acyldiazene **4**, which was produced by the NO*_x_*-catalyzed aerobic oxidation of acylhydrazide **1**, provided intermediate **B**. Then, intramolecular 1,4-addition followed by elimination yielded the desired 1,3,4-oxadiazine **3** and DMAP catalyst.

## 3. Materials and Methods

### 3.1. General Information

All commercially available compounds and solvents were purchased and used as received, unless otherwise noted. Analytical thin-layer chromatography (TLC) was performed on precoated silica gel 60 F254 plates. TLC visualization was achieved by the use of UV light (254 nm) and treatment with phosphomolybdic acid, *p*-anisaldehyde, KMnO_4_, or Vanillin stain followed by heating. Flash chromatography was performed using silica gel (particle size 40−63 μm, 230−400 mesh). ^1^H and ^13^C NMR spectra were recorded using 300 MHz NMR (300 MHz for ^1^H, 75 MHz for ^13^C) or 400 MHz NMR (400 MHz for ^1^H, 101 MHz for ^13^C). Chemical shift values are given in parts per million relative to internal TMS (0.00 ppm for ^1^H) or CDCl_3_ (77.06 ppm for ^13^C). The following abbreviations were used to describe peak splitting patterns when appropriate: br = broad, s = singlet, d = doublet, t = triplet, q = quartet, p = pentet, m = multiplet, dd = double of doublet, dt = double of triplet, td = triple of doublet, tt = triple of triplet. Coupling constants, *J*, were reported in hertz unit (Hz). High-resolution mass spectra were obtained from the Korea Basic Science Institute (Daegu) by using the EI method and magnetic sector mass analyzer. Melting points were determined on a digital melting point apparatus, and temperatures were uncorrected.

### 3.2. Preparation of Acylhydrazides and Allenoates

#### 3.2.1. Preparation of Acylhydrazides (**1a–1j**, **1l–1u**, and **1w–1x**) [49]

To a 50 mL round-bottom flask equipped with a magnetic stir bar, hydrazine hydrochloride (5.0 mmol) and CH_2_Cl_2_ (5.0 mL) were added. The solution was cooled to 0 °C, and pyridine (11.0 mmol, 2.2 equiv) was added. Then, acyl chloride (5.5 mmol, 1.1 equiv) was added dropwise. The reaction mixture was stirred at room temperature for 4 h. The mixture was diluted with CH_2_Cl_2_ and washed with 1.0 M HCl aqueous solution three times; then, the combined organic layer was dried over MgSO_4_, filtered, and concentrated on a rotary evaporator. Recrystallization with EtOH yielded the desired acylhydrazide.

#### 3.2.2. Preparation of *N′*-(*tert*-butyl)benzohydrazide (**1k**) [51]

To a 100 mL round-bottom flask equipped with a magnetic stir bar, *tert*-butylhydrazine hydrochloride (10.0 mmol), Et_3_N (22.0 mmol, 2.2 equiv), and CH_2_Cl_2_ (20.0 mL) were added. The solution was cooled to 0 °C, and benzoyl chloride (10.0 mmol, 1.0 equiv) was added dropwise. The reaction mixture was stirred overnight at room temperature. The mixture was washed with water three times; then, the combined organic layer was dried over MgSO_4_, filtered, and concentrated on a rotary evaporator. Recrystallization with EtOH yielded the desired *N*′-(*tert*-butyl)benzohydrazide.

#### 3.2.3. Preparation of *N′*-phenylcyclohexanecarbohydrazide (**1v**)

A 100 mL flame-dried round-bottom flask, which was equipped with a magnetic stir bar and charged with phenylhydrazine hydrochloride (12.0 mmol), was evacuated and backfilled with nitrogen (this process was repeated three times). After CH_2_Cl_2_ (30.0 mL) was added, the solution was cooled to 0 °C. To the reaction mixture, pyridine (24.0 mmol, 2.0 equiv) was added slowly, and then cyclohexanecarbonyl chloride (13.2 mmol, 1.1 equiv) was added dropwise. The reaction mixture was stirred at room temperature for 5 h. The mixture was diluted with CH_2_Cl_2_ and washed with 4.0 M HCl aqueous solution three times; then, the combined organic layer was dried over MgSO_4_, filtered, and concentrated on a rotary evaporator. Recrystallization with 1:4 EtOAc/Hx yielded the desired acylhydrazide.

#### 3.2.4. Preparation of Allenoates [52]

A 100 mL round-bottom flask, which was equipped with a magnetic stir bar and charged with triphenylphosphorane (10.0 mmol), was evacuated and backfilled with nitrogen (this process was repeated three times). After CH_2_Cl_2_ (40 mL) and trimethylamine (11.0 mmol, 1.1 equiv) were added, acyl chloride (11.0 mmol, 1.1 equiv) was added dropwise at 0 °C. The reaction mixture was stirred at room temperature overnight. The mixture was filtered by a short pad of silica and concentrated on a rotary evaporator. The pure allenoates were obtained by column chromatography.

Ethyl 6-methylhepta-2,3-dienoate (for **3aa**); colorless oil, EtOAc/Hx = 1:10, ^1^H NMR (300 MHz, CDCl_3_) δ 5.61–5.52 (m, 2H), 4.20–4.12 (m, 2H), 2.07–1.99 (m, 2H), 1.74 (dt, *J* = 13.3, 6.7 Hz, 1H), 1.27 (t, *J* = 7.1 Hz, 3H), 0.96 (dd, *J* = 6.6, 1.6 Hz, 6H); ^13^C NMR (75 MHz, CDCl_3_) δ 212.6, 166.2, 93.7, 87.6, 60.6, 36.8, 28.2, 22.1, 21.9, 14.2; HRMS (EI) *m*/*z* calcd. For C_10_H_16_O_2_ [M]^+^: 168.1150, found 168.1152.

Methyl hexa-2,3-dienoate (for **3ad**); colorless oil, EtOAc/Hx = 1:10, ^1^H NMR (400 MHz, CDCl_3_) δ 5.69 (q, *J* = 6.3 Hz, 1H), 5.62 (dt, *J* = 6.2, 3.4 Hz, 1H), 3.74 (s, 3H), 2.24–2.08 (m, 2H), 1.08 (t, *J* = 7.4 Hz, 3H); ^13^C NMR (101 MHz, CDCl_3_) δ 212.2, 166.7, 97.1, 88.5, 51.9, 20.8, 13.0; HRMS (EI) *m*/*z* calcd. For C_7_H_10_O_2_ [M]^+^: 126.0681, found 126.0680.

### 3.3. General Procedure for One-Pot Synthesis of 1,3,4-Oxadiazines

One 10 mL flame-dried test tube (Tube A), which was equipped with a magnetic stir bar and charged with acylhydrazide (0.3 mmol) and NaNO_2_ (0.03 mmol, 10 mol %), was evacuated and backfilled with oxygen (this process was repeated three times). After toluene (1.0 mL), HNO_3_ (0.06 mmol, 20 mol %), and additional toluene (0.5 mL) were added in sequence, the reaction mixture was stirred for 2 h. The other 10 mL flame-dried test tube (Tube B), which was equipped with a magnetic stir bar, was evacuated and backfilled with nitrogen (this process was repeated three times). Allenoate (0.36 mmol, 1.2 equiv) in toluene (0.5 mL) was added to Tube B. Then, the reaction mixture in Tube A was added to Tube B using a syringe. By using toluene (0.5 mL), Tube A was washed, and the solution was transferred to Tube B. After the combined mixture in Tube B was stirred at room temperature for 0.5 h, DMAP (0.09 mmol, 30 mol %) in toluene (0.5 mL) was added. After 48 h, the reaction mixture in Tube B was diluted by adding CH_2_Cl_2_ and washed with a saturated aqueous solution of Na_2_CO_3_. Two layers were separated, and the aqueous layer was extracted with CH_2_Cl_2_. The combined organic layer was dried over MgSO_4_, filtered, and concentrated on a rotary evaporator. The residue was purified by column chromatography to yield 1,3,4-oxadiazines.

### 3.4. Characterizations of the Newly Synthesized 1,3,4-Oxadiazines

(*Z*)-Ethyl 2-(5-benzyl-4-(4-methoxyphenyl)-2-phenyl-4*H*-1,3,4-oxadiazin-6(5*H*)-ylidene)acetate (**3b**); yellow oil, EtOAc/Hx = 1:10, ^1^H NMR (400 MHz, CDCl_3_) δ 8.11 (d, *J* = 6.9 Hz, 2H), 7.45 (d, *J* = 6.4 Hz, 3H), 7.29–7.14 (m, 8H), 6.92 (d, *J* = 8.8 Hz, 2H), 4.72 (s, 1H), 4.59 (dd, *J* = 9.2, 4.6 Hz, 1H), 4.25–4.15 (m, 2H), 3.82 (s, 3H), 2.98–2.84 (m, 2H), 1.30 (t, *J* = 7.1 Hz, 3H); ^13^C NMR (75 MHz, CDCl_3_) δ 164.5, 154.6, 153.3, 141.8, 138.8, 136.5, 130.1, 129.8, 129.5, 128.7, 128.5, 127.0, 125.6, 116.1, 114.7, 98.0, 60.1, 57.4, 55.7, 33.5, 14.3; HRMS (EI) *m*/*z* calcd. For C_27_H_26_N_2_O_4_ [M]^+^: 442.1893, found 442.1895.

(*Z*)-Ethyl 2-(5-benzyl-4-(4-nitrophenyl)-2-phenyl-4*H*-1,3,4-oxadiazin-6(5*H*)-ylidene)acetate (**3f**); yellow oil, EtOAc/Hx = 1:10, ^1^H NMR (400 MHz, CDCl_3_) δ 8.06–7.96 (m, 4H), 7.49 (d, *J* = 7.0 Hz, 3H), 7.20–7.04 (m, 7H), 6.65 (dd, *J* = 8.2, 5.8 Hz, 1H), 5.73 (s, 1H), 4.22–4.14 (m, 2H), 3.10–2.99 (m, 2H), 1.29 (t, *J* = 7.1 Hz, 3H); ^13^C NMR (101 MHz, CDCl_3_) δ 166.2, 158.3, 149.6, 144.2, 140.0, 135.2, 130.9, 129.9, 129.1, 128.6, 128.5, 127.3, 125.8, 125.5, 112.3, 99.0, 60.6, 50.6, 35.2, 14.3; HRMS (EI) *m*/*z* calcd. For C_26_H_23_N_3_O_5_ [M]^+^: 457.1638, found 457.1635.

(*Z*)-Ethyl 2-(5-benzyl-2-phenyl-4-(*m*-tolyl)-4H-1,3,4-oxadiazin-6(5*H*)-ylidene)acetate (**3g**); yellow solid, EtOAc/Hx = 1:10, mp 102–103 °C, ^1^H NMR (400 MHz, CDCl_3_) δ 8.18–8.04 (m, 2H), 7.48–7.43 (m, 3H), 7.30–7.25 (m, 2H), 7.24–7.17 (m, 3H), 7.15 (s, 1H), 7.10 (s, 1H), 7.03 (dd, *J* = 8.2, 2.2 Hz, 1H), 6.82–6.76 (m, 1H), 4.75 (s, 1H), 4.67 (dd, *J* = 9.4, 4.8 Hz, 1H), 4.35–4.14 (m, 2H), 3.11–2.84 (m, 2H), 2.37 (s, 3H), 1.41–1.23 (m, 3H); ^13^C NMR (75 MHz, CDCl_3_) δ 164.5, 153.0, 144.6, 141.8, 139.3, 136.4, 130.1, 129.9, 129.5, 129.2, 128.7, 128.5, 127.1, 125.7, 121.9, 115.0, 111.2, 98.2, 60.1, 56.3, 34.0, 21.9, 14.4; HRMS (EI) *m*/*z* calcd. For C_27_H_26_N_2_O_3_ [M]^+^: 426.1943, found 426.1941.

(*Z*)-Ethyl 2-(5-benzyl-4-(3-bromophenyl)-2-phenyl-4*H*-1,3,4-oxadiazin-6(5*H*)-ylidene)acetate (**3h**); yellow oil, EtOAc/Hx = 1:10, ^1^H NMR (400 MHz, CDCl_3_) δ 8.12 (d, *J* = 4.2 Hz, 2H), 7.47 (s, 3H), 7.43 (s, 1H), 7.28 (s, 2H), 7.23 (d, *J* = 7.4 Hz, 1H), 7.16 (d, *J* = 7.3 Hz, 3H), 7.10–7.04 (m, 2H), 4.83 (s, 1H), 4.68–4.63 (m, 1H), 4.22 (d, *J* = 6.4 Hz, 2H), 3.01–2.89 (m, 2H), 1.31 (t, *J* = 7.1 Hz, 3H); ^13^C NMR (101 MHz, CDCl_3_) δ 164.3, 152.6, 145.7, 142.4, 135.8, 130.5, 130.2, 129.6, 129.5, 128.7, 128.5, 127.3, 125.8, 123.5, 123.4, 117.1, 112.0, 98.6, 60.2, 55.9, 34.3, 14.3; HRMS (EI) *m*/*z* calcd. For C_26_H_23_BrN_2_O_3_ [M]^+^: 490.0892, found 490.0895.

(*Z*)-Ethyl 2-(5-benzyl-2-(4-nitrophenyl)-4-phenyl-4*H*-1,3,4-oxadiazin-6(5*H*)-ylidene)acetate (**3q**); orange solid, EtOAc/Hx =1:10, mp 75–76 °C, ^1^H NMR (400 MHz, CDCl_3_) δ 8.31–8.19 (m, 4H), 7.43–7.34 (m, 2H), 7.34–7.24 (m, 4H), 7.23–7.13 (m, 3H), 7.03 (t, *J* = 7.2 Hz, 1H), 4.88 (s, 1H), 4.77 (dd, *J* = 9.1, 4.7 Hz, 1H), 4.27–4.17 (m, 2H), 3.07–2.89 (m, 2H), 1.31 (t, *J* = 7.1 Hz, 3H); ^13^C NMR (101 MHz, CDCl_3_) δ 164.2, 152.1, 148.2, 143.8, 139.7, 135.9, 135.7, 129.5, 129.5, 128.7, 127.3, 126.1, 123.8, 121.8, 114.2, 99.0, 60.3, 56.0, 34.7, 14.3; HRMS (EI) *m*/*z* calcd. For C_26_H_23_N_3_O_5_ [M]^+^: 457.1638, found 457.1639.

(*Z*)-Ethyl 2-(5-benzyl-4-phenyl-2-(*m*-tolyl)-4*H*-1,3,4-oxadiazin-6(5*H*)-ylidene)acetate (**3r**); yellow oil, EtOAc/Hx = 1:10, ^1^H NMR (300 MHz, CDCl_3_) δ 7.94 (d, *J* = 7.5 Hz, 2H), 7.39–7.32 (m, 3H), 7.31–7.20 (m, 6H), 7.20–7.14 (m, 2H), 7.00–6.91 (m, 1H), 4.74 (s, 1H), 4.68 (dd, *J* = 9.5, 4.9 Hz, 1H), 4.20 (dd, *J* = 7.1, 5.6 Hz, 2H), 3.01–2.84 (m, 2H), 2.45 (s, 3H), 1.31 (t, *J* = 7.1 Hz, 3H); ^13^C NMR (75 MHz, CDCl_3_) δ 164.6, 153.0, 144.6, 142.2, 138.2, 136.4, 130.9, 130.0, 129.5, 129.4, 128.8, 128.5, 127.2, 126.2, 123.1, 121.0, 114.2, 98.4, 60.2, 56.3, 33.9, 21.6, 14.4; HRMS (EI) *m*/*z* calcd. For C_27_H_26_N_2_O_3_ [M]^+^: 426.1943, found 426.1939.

(*Z*)-Ethyl 2-(5-benzyl-2-(3-chlorophenyl)-4-phenyl-4*H*-1,3,4-oxadiazin-6(5*H*)-ylidene)acetate (**3s**); yellow oil, EtOAc/Hx = 1:10, ^1^H NMR (300 MHz, CDCl_3_) δ 8.07 (q, *J* = 1.3 Hz, 1H), 7.99 (dd, *J* = 5.5, 1.8 Hz, 1H), 7.42–7.33 (m, 4H), 7.32–7.21 (m, 5H), 7.18–7.13 (m, 2H), 6.99 (d, *J* = 7.2 Hz, 1H), 4.78 (s, 1H), 4.71 (dd, *J* = 9.3, 4.8 Hz, 1H), 4.21 (dd, *J* = 7.1, 5.0 Hz, 2H), 3.02–2.84 (m, 2H), 1.32 (t, *J* = 7.1 Hz, 3H); ^13^C NMR (75 MHz, CDCl_3_) δ 164.4, 152.4, 144.2, 140.6, 136.1, 134.6, 131.8, 129.8, 129.8, 129.5, 129.4, 128.7, 127.2, 125.6, 123.8, 121.3, 114.1, 98.8, 60.3, 56.2, 34.1, 14.4; HRMS (EI) *m*/*z* calcd. For C_26_H_23_ClN_2_O_3_ [M]^+^: 446.1397, found 446.1400.

(*Z*)-Ethyl 2-(5-benzyl-2-(naphthalen-2-yl)-4-phenyl-4*H*-1,3,4-oxadiazin-6(5*H*)-ylidene)acetate (**3t**); yellow solid, EtOAc/Hx = 1:10, mp 66–67 °C, ^1^H NMR (400 MHz, CDCl_3_) δ 8.61 (s, 1H), 8.25 (d, *J* = 8.6 Hz, 1H), 8.05–7.98 (m, 1H), 7.96–7.85 (m, 2H), 7.63–7.52 (m, 2H), 7.46–7.18 (m, 9H), 7.02 (t, *J* = 6.8 Hz, 1H), 4.82 (s, 1H), 4.76 (dd, *J* = 9.3, 4.6 Hz, 1H), 4.28 (dd, *J* = 12.4, 6.9 Hz, 2H), 3.07–2.94 (m, 2H), 1.39 (t, *J* = 7.1 Hz, 3H); ^13^C NMR (75 MHz, CDCl_3_) δ 164.6, 152.9, 144.5, 142.1, 136.3, 134.1, 133.1, 129.5, 129.4, 128.8, 128.7, 128.2, 127.8, 127.4, 127.2, 126.9, 126.5, 125.4, 123.0, 121.1, 114.1, 98.5, 60.2, 56.3, 34.1, 14.5; HRMS (EI) calcd, for C_30_H_26_N_2_O_3_ [M]^+^: 462.1943, found: 462.1947.

(*Z*)-Ethyl 2-(5-benzyl-2-cyclohexyl-4-phenyl-4*H*-1,3,4-oxadiazin-6(5*H*)-ylidene)acetate (**3v**); yellow oil, EtOAc/Hx = 1:10, ^1^H NMR (300 MHz, CDCl_3_) δ 7.33–7.26 (m, 3H), 7.26–7.21 (m, 2H), 7.19–7.11 (m, 4H), 6.91 (d, *J* = 7.2 Hz, 1H), 4.61 (s, 1H), 4.54 (dd, *J* = 9.3, 5.0 Hz, 1H), 4.25–4.11 (m, 2H), 2.90–2.74 (m, 2H), 2.60–2.44 (m, 1H), 2.14–2.05 (m, 2H), 1.85 (d, *J* = 10.6 Hz, 2H), 1.67 (dd, *J* = 27.1, 13.4 Hz, 3H), 1.41–1.29 (m, 3H), 1.28–1.24 (m, 3H); ^13^C NMR (75 MHz, CDCl_3_) δ 164.4, 153.8, 149.1, 144.9, 136.4, 129.5, 129.3, 128.6, 127.0, 120.4, 113.9, 97.6, 59.9, 56.1, 40.7, 32.8, 29.7, 25.9, 25.7, 14.3; HRMS (EI) *m*/*z* calcd. For C_26_H_30_N_2_O_3_ [M]^+^: 418.2256, found 418.2254.

(*Z*)-Ethyl 2-(5-benzyl-2-(4-bromophenyl)-4-(*m*-tolyl)-4*H*-1,3,4-oxadiazin-6(5*H*)-ylidene)acetate (**3w**); yellow solid, EtOAc/Hx = 1:10, mp 95–96 °C, ^1^H NMR (400 MHz, CDCl_3_) δ 8.06–7.89 (m, 2H), 7.64–7.52 (m, 2H), 7.29 (d, *J* = 7.9 Hz, 2H), 7.23 (d, *J* = 7.8 Hz, 2H), 7.16 (d, *J* = 7.3 Hz, 2H), 7.09 (s, 1H), 7.04 (d, *J* = 8.3 Hz, 1H), 6.80 (d, *J* = 7.4 Hz, 1H), 4.78 (s, 1H), 4.69 (dd, *J* = 9.5, 4.7 Hz, 1H), 4.31–4.11 (m, 2H), 3.01–2.85 (m, 2H), 2.39 (s, 3H), 1.29 (dd, *J* = 7.9, 6.3 Hz, 3H); ^13^C NMR (75 MHz, CDCl_3_) δ 164.4, 152.8, 144.3, 141.0, 139.3, 136.2, 131.6, 129.5, 129.2, 129.0, 128.8, 128.7, 127.2, 124.2, 122.1, 115.0, 111.2, 98.4, 60.1, 56.2, 34.2, 21.9, 14.3; HRMS (EI) *m*/*z* calcd. For C_27_H_25_BrN_2_O_3_ [M]^+^: 504.1049, found 504.1050.

(*Z*)-Ethyl 2-(5-isobutyl-2,4-diphenyl-4*H*-1,3,4-oxadiazin-6(5*H*)-ylidene)acetate (**3aa**); yellow oil, EtOAc/Hx = 1:10, ^1^H NMR (300 MHz, CDCl_3_) δ 8.14–8.05 (m, 2H), 7.47–7.39 (m, 3H), 7.37–7.31 (m, 2H), 7.29–7.23 (m, 2H), 6.95 (tt, *J* = 7.2, 1.2 Hz, 1H), 5.20 (s, 1H), 4.61 (dd, *J* = 10.9, 3.9 Hz, 1H), 4.30–4.22 (m, 2H), 1.74–1.62 (m, 2H), 1.35 (t, *J* = 7.1 Hz, 4H), 1.04 (d, *J* = 6.4 Hz, 3H), 0.90 (d, *J* = 6.5 Hz, 3H); ^13^C NMR (75 MHz, CDCl_3_) δ 164.7, 154.0, 144.6, 141.4, 130.0, 129.8, 129.3, 128.4, 125.6, 120.8, 113.8, 97.5, 60.2, 52.2, 35.1, 24.7, 23.5, 21.4, 14.4; HRMS (EI) *m*/*z* calcd. For C_23_H_26_N_2_O_3_ [M]^+^: 378.1943, found 378.1945.

(*Z*)-Ethyl 2-(2,4,5-triphenyl-4*H*-1,3,4-oxadiazin-6(5*H*)-ylidene)acetate (**3ab**); yellow oil, EtOAc/Hx = 1:10, ^1^H NMR (300 MHz, CDCl_3_) δ 8.10–8.01 (m, 2H), 7.42–7.37 (m, 3H), 7.34–7.22 (m, 9H), 7.00–6.89 (m, 1H), 5.71 (s, 1H), 5.52 (s, 1H), 4.26 (q, *J* = 7.1 Hz, 2H), 1.35 (t, *J* = 7.2 Hz, 3H); ^13^C NMR (126 MHz, CDCl_3_) δ 164.7, 153.9, 144.9, 141.5, 134.6, 130.0, 129.8, 129.3, 129.1, 128.5, 128.4, 126.8, 125.6, 121.0, 113.9, 98.2, 60.4, 57.0, 14.4; HRMS (EI) *m*/*z* calcd. For C_25_H_22_N_2_O_3_ [M]^+^: 398.1630, found 398.1634.

(*Z*)-Benzyl 2-(5-benzyl-2,4-diphenyl-4*H*-1,3,4-oxadiazin-6(5*H*)-ylidene)acetate (**3ac**); yellow oil, EtOAc/Hx =1:10, ^1^H NMR (300 MHz, CDCl_3_) δ 8.04–7.98 (m, 2H), 7.44–7.31 (m, 10H), 7.29–7.19 (m, 5H), 7.18–7.13 (m, 2H), 6.96 (tt, *J* = 7.1, 1.3 Hz, 1H), 5.23–5.11 (m, 2H), 4.80 (s, 1H), 4.70 (dd, *J* = 9.5, 4.9 Hz, 1H), 3.03–2.81 (m, 2H); ^13^C NMR (75 MHz, CDCl_3_) δ 164.2, 153.4, 144.5, 141.9, 136.2, 136.0, 129.8, 129.5, 129.4, 128.7, 128.6, 128.4, 128.3, 128.2, 127.1, 125.7, 121.0, 114.1, 98.0, 66.1, 56.2, 33.9.; HRMS (EI) *m*/*z* calcd. For C_31_H_26_N_2_O_3_ [M]^+^: 474.1943, found 474.1944.

(*Z*)-Methyl 2-(5-ethyl-2,4-diphenyl-4*H*-1,3,4-oxadiazin-6(5*H*)-ylidene)acetate (**3ad**); yellow oil, EtOAc/Hx = 1:10, ^1^H NMR (300 MHz, CDCl_3_) δ 8.10–7.99 (m, 2H), 7.47–7.23 (m, 7H), 6.94 (tt, *J* = 7.0, 1.3 Hz, 1H), 5.20 (s, 1H), 4.43 (dd, *J* = 8.0, 6.6 Hz, 1H), 3.79 (s, 3H), 1.83–1.65 (m, 2H), 0.99 (t, *J* = 7.5 Hz, 3H); δ ^13^C NMR (75 MHz, CDCl_3_) δ 165.0, 154.1, 144.7, 141.4, 130.0, 129.7, 129.3, 128.4, 125.6, 120.7, 113.9, 97.2, 55.4, 51.4, 20.4, 10.7; HRMS (EI) *m*/*z* calcd. For C_20_H_20_N_2_O_3_ [M]^+^: 336.1474, found 336.1471.

### 3.5. ^1^H and ^13^C NMR Spectra

For the ^1^H and ^13^C NMR spectra, see Supplementary Materials.

## 4. Conclusions

In conclusion, we have developed a practical and green one-pot synthesis of 1,3,4-oxadiazines from acylhydrazides. The newly developed one-pot protocol consists of aerobic oxidations of acylhydrazides into *N*-acyldiazenes using a NO*_x_* catalytic system, followed by the DMAP-catalyzed cycloaddition of allenoate with the generated *N*-acyldiazenes. The present method was able to utilize various acylhydrazides to generate 1,3,4-oxadiazines with good to high yields. Interestingly, the electron-deficient phenyl-substituted 1,3,4-oxadiazines, which could not be synthesized by the previous method using *N*-acyldiazene, were able to be synthesized by the present one-pot method. However, aliphatic acylhydrazides displayed limited substrate scope. The practicality of the one-pot synthesis of 1,3,4-oxadiazines from acylhydrazides was demonstrated by the gram-scale experiment, which was not achieved by the previous synthesis of 1,3,4-oxadiazines from *N*-acyldiazenes. The synthesis of other heterocycles using *N*-acyldiazenes or acylhydrazides is underway in our laboratory.

## Figures and Tables

**Table 1:** Direct aerobic oxidative cyclization of *N′*-phenylbenzohydrazide (**1a**) and benzyl allenoate (**2a**) *^a^*.

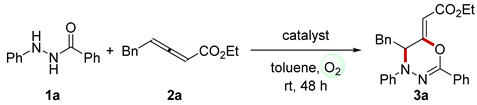		
Entry	Catalyst (mol %)	Yield (%) *^b^*
1	CuCl (10)/DMAP (60)	14
2	Fe(Pc) (10)/DMAP (40)	15
3	Mn(Pc) (10)/DMAP (40)	19
4	NaNO_2_ (10)/HNO_3_ (20)/DMAP (40)	30

*^a^* Reaction conditions: **1a** (0.3 mmol), **2a** (0.45 mmol), and catalyst in toluene (3.0 mL) under O_2_ for 48 h. *^b^* Isolated yield.

**Table 2:** Optimization of the one-pot synthesis of 1,3,4-oxadiazine (**3a**) from *N*′-phenylbenzohydrazide (**1a**) and benzyl allenoate (**2a**) *^a^*.

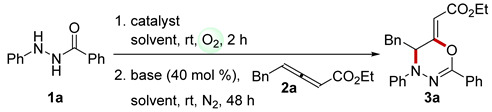				
Entry	Catalyst (mol %)	Base	Solvent	Yield (%) *^b^*
1	CuCl (10)/DMAP (20)	DMAP	toluene	24
2	Fe(Pc) (10)	DMAP	toluene	30
3	Mn(Pc) (10)	DMAP	toluene	13
4	NaNO_2_ (10)/HNO_3_ (20)	DMAP	toluene	75
5	NaNO_2_ (10)/HNO_3_ (20)	pyridine	toluene	13
6	NaNO_2_ (10)/HNO_3_ (20)	DBU	toluene	0
7	NaNO_2_ (10)/HNO_3_ (20)	DMAP	CH_3_CN	20
8	NaNO_2_ (10)/HNO_3_ (20)	DMAP	CH_2_Cl_2_	46
9	NaNO_2_ (10)/HNO_3_ (20)	DMAP	1,4-dioxane	45
10	NaNO_2_ (10)/HNO_3_ (20)	DMAP	EtOH	25
11 *^c^*	NaNO_2_ (10)/HNO_3_ (20)	DMAP	toluene	76

*^a^* Reaction conditions: **1a** (0.3 mmol) and catalyst in solvent (1.5 mL) under O_2_ at room temperature for 2 h were used for the first step. The reaction mixture of the first step was added to the base (40 mol %) and **2a** (0.45 mmol) in solvent (1.5 mL) under N_2_ at room temperature; then, the reaction mixture of the second step was stirred for 48 h. *^b^* Isolated yield. *^c^* The use of 1.2 equiv of **2a** and 30 mol % of DMAP.

## Data Availability

Not applicable.

## Supplementary Materials

The following supporting information can be downloaded at https://www.mdpi.com/article/10.3390/molecules28093815/s1, Preparation of acylhydrazides and allenoates, detailed optimizations, experimental procedures, spectroscopic data, and copies of ^1^H and ^13^C NMR spectra.

## Sample Availability

Not available.
