# Supplementary material for: One-Pot Synthesis of 1,3,4-Oxadiazines from Acylhydrazides and Allenoates
Source: Molecules. 2023 Apr 29;28(9):3815. doi: 10.3390/molecules28093815 (PMC10180079; doi:10.3390/molecules28093815)

Supporting Information for

One-pot Synthesis of 1,3,4-Oxadiazines from Acylhydrazides and Allenoates

Su Been Kim,[1,†] Santanu Maiti,[1,†] Eun Sun Park,[1] Ga Young Kim,[1] Yunji Choun,[1] Soon Kil Ahn,[2] Jae Kwang Kim,[3] and Jinho Kim[1,*]

[1]*Department of Chemistry, and Research Institute of Basic Sciences,*

Incheon National University, Incheon 22012, Republic of Korea.

jinho@inu.ac.kr

[2]*Institute for New Drug Development, Division of Life Sciences,*

Incheon National University, Incheon 22012, Republic of Korea

[3]*Division of Life Sciences, Incheon National University, Incheon 22012, Republic of Korea*

[†]*These authors contributed equally*

Table of Contents

1.	General information	S1
2.	Preparation of acylhydrazides and allenoates	S2
3.	Experimental procedure for Table 1	S3
4.	Optimization of one-pot synthesis of 1,3,4-oxadiazine	S4
5.	General procedure for one-pot synthesis of 1,3,4-oxadiazines	S5
6.	Scale-up process for one-pot synthesis of **3a**	S14
7.	References	S15
8.	^{1}H and ^{13}C NMR spectra	S16

1. General information

All commercially available compounds and solvents were used as received, unless otherwise noted. Analytical thin-layer chromatography (TLC) was performed on precoated silica gel 60 F254 plates. Visualization on TLC was achieved with UV light (254 nm) and staining with phosphomolybdic acid, *p*-anisaldehyde, $KMnO_4$, or vanillin, followed by heating. Flash chromatography was performed using silica gel (particle size 40−63 μm, 230−400 mesh). ^{1}H and ^{13}C NMR spectra were recorded on 300 MHz NMR (300 MHz for ^{1}H, 75 MHz for ^{13}C) or 400 MHz NMR (400 MHz for ^{1}H, 101 MHz for ^{13}C). Chemical shift values are given in parts per million relative to internal TMS (0.00 ppm for ^{1}H) or $CDCl_3$ (77.06 ppm for ^{13}C). The following abbreviations were used to describe peak splitting patterns when

appropriate: br = broad, s = singlet, d = doublet, t = triplet, q = quartet, p = pentet, m = multiplet, dd = double of doublet, dt = double of triplet, td = triple of doublet, tt = triple of triplet. Coupling constants, *J*, were reported in hertz unit (Hz). High-resolution mass spectra were obtained using EI method and magnetic sector mass analyzer at the Korea Basic Science Institute (Daegu). Melting points were determined on a digital apparatus and the reported temperatures were uncorrected.

2. Preparation of acylhydrazides and allenoates

Preparation of acylhydrazides (1a–1j, 1l–1u, and 1w–1x)[1]

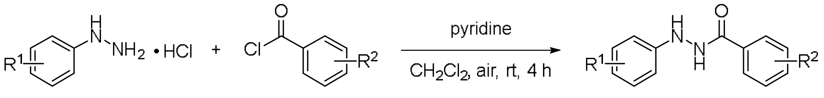

To a 50 mL round-bottom flask equipped with a magnetic stir bar, hydrazine hydrochloride (5.0 mmol) and CH_2Cl_2 (5.0 mL) were added. The solution was cooled to 0 °C, and pyridine (11.0 mmol, 2.2 equiv) was added. Then, acyl chloride (5.5 mmol, 1.1 equiv) was added dropwise. The reaction mixture was stirred at room temperature for 4 h. The mixture was diluted with CH_2Cl_2 and washed with 1.0 M HCl aqueous solution three times, then the combined organic layer was dried over $MgSO_4$, filtered, and concentrated on a rotary evaporator. Recrystallization with EtOH yielded the desired acylhydrazide.

Preparation of *N'*-(*tert*-butyl)benzohydrazide (1k)[2]

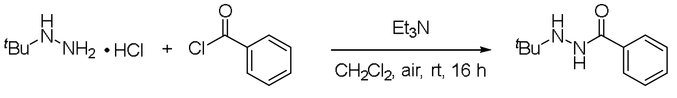

To a 100 mL round-bottom flask equipped with a magnetic stir bar, *tert*-butylhydrazine hydrochloride (10.0 mmol), Et_3N (22.0 mmol, 2.2 equiv), and CH_2Cl_2 (20.0 mL) were added. The solution was cooled to 0 °C, and benzoyl chloride (10.0 mmol, 1.0 equiv) was added dropwise. The reaction mixture was stirred overnight at room temperature. The mixture was washed with water three times, then the combined organic layer was dried over $MgSO_4$, filtered, and concentrated on a rotary evaporator. Recrystallization with EtOH yielded the desired *N'*-(*tert*-butyl)benzohydrazide.

Preparation of *N'*-phenylcyclohexanecarbohydrazide (1v)

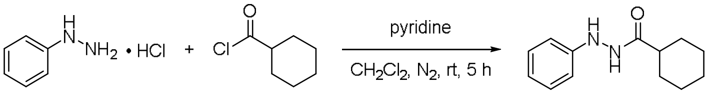

A 100 mL flame-dried round-bottom flask, which was equipped with a magnetic stir bar and charged with phenylhydrazine hydrochloride (12.0 mmol), was evacuated and backfilled with nitrogen (this process was repeated three times). After CH_2Cl_2 (30.0 mL) were added, the solution was cooled to 0 °C. To the reaction mixture, pyridine (24.0 mmol, 2.0 equiv) was added slowly, then, cyclohexanecarbonyl chloride (13.2 mmol, 1.1 equiv) was added dropwise. The reaction mixture was stirred at room

temperature for 5 h. The mixture was diluted with CH_2Cl_2 and washed with 4.0 M HCl aqueous solution three times, then the combined organic layer was dried over $MgSO_4$, filtered, and concentrated on a rotary evaporator. Recrystallization with 1:4 EtOAc/Hx yielded the desired acylhydrazide.

Preparation of allenoates[3]

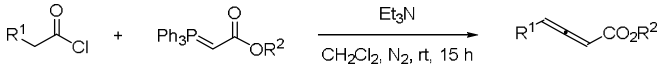

A 100 mL flame-dried round-bottom flask, which was equipped with a magnetic stir bar and charged with triphenylphosphorane (10.0 mmol), was evacuated and backfilled with nitrogen (this process was repeated three times). After CH_2Cl_2 (40 mL) and trimethylamine (11.0 mmol, 1.1 equiv) were added, acyl chloride (11.0 mmol, 1.1 equiv) was added dropwise at 0 °C. The reaction mixture was stirred at room temperature overnight. The mixture was filtered by short pad of silica and concentrated on a rotary evaporator. The pure allenoates were obtained by column chromatography.

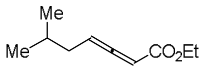

Ethyl 6-methylhepta-2,3-dienoate (for 3aa); colorless oil, EtOAc/Hx = 1:10, ^{1}H NMR (300 MHz, $CDCl_3$) δ 5.61 – 5.52 (m, 2H), 4.20 – 4.12 (m, 2H), 2.07 – 1.99 (m, 2H), 1.74 (dt, J = 13.3, 6.7 Hz, 1H), 1.27 (t, J = 7.1 Hz, 3H), 0.96 (dd, J = 6.6, 1.6 Hz, 6H); ^{13}C NMR (75 MHz, $CDCl_3$) δ 212.6, 166.2, 93.7, 87.6, 60.6, 36.8, 28.2, 22.1, 21.9, 14.2; HRMS (EI) m/z calcd. For $C_{10}H_{16}O_2$ $[M]^+$: 168.1150, found 168.1152.

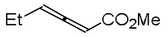

Methyl hexa-2,3-dienoate (for 3ad); colorless oil, EtOAc/Hx = 1:10, ^{1}H NMR (400 MHz, $CDCl_3$) δ 5.69 (q, J = 6.3 Hz, 1H), 5.62 (dt, J = 6.2, 3.4 Hz, 1H), 3.74 (s, 3H), 2.24 – 2.08 (m, 2H), 1.08 (t, J = 7.4 Hz, 3H); ^{13}C NMR (101 MHz, $CDCl_3$) δ 212.2, 166.7, 97.1, 88.5, 51.9, 20.8, 13.0; HRMS (EI) m/z calcd. For $C_7H_{10}O_2$ $[M]^+$: 126.0681, found 126.0680.

3. Experimental procedure for Table 1

Entry 1

A 10 mL flame-dried test tube, which was equipped with a magnetic stir bar and charged with **1a** (0.3 mmol), CuCl (0.03 mmol, 10 mol %), and DMAP (0.18 mmol, 60 mol %), was evacuated and backfilled with oxygen (this process was repeated three times). After toluene (1.5 mL) and **2a** (0.45 mmol, 1.5 equiv) in toluene (1.5 mL) was added to the test tube, the reaction mixture was stirred at room temperature. After 48 h, mixture was diluted by adding CH_2Cl_2 and washed with a saturated aqueous solution of NH_4Cl. Two layers were separated, and the aqueous layer was extracted with CH_2Cl_2. The

combined organic layer was dried over $MgSO_4$, filtered, and concentrated on a rotary evaporator. The residue was purified by column chromatography (EtOAc/Hx = 1:10) to give 1,3,4-oxadiazine **3a**.

Entry 2 and 3

A 10 mL flame-dried test tube, which was equipped with a magnetic stir bar and charged with **1a** (0.3 mmol), M(Pc) (M=Fe, Mn) (0.03 mmol, 10 mol %), and DMAP (0.12 mmol, 40 mol %), was evacuated and backfilled with oxygen (this process was repeated three times). After toluene (1.5 mL) and **2a** (0.45 mmol, 1.5 equiv) in toluene (1.5 mL) was added to the test tube, the reaction mixture was stirred at room temperature. After 48 h, the reaction mixture was filtered through pad of silica. The filtrate was concentrated on a rotary evaporator. The residue was purified by column chromatography (EtOAc/Hx = 1:10) to give 1,3,4-oxadiazine **3a**.

Entry 4

A 10 mL flame-dried test tube, which was equipped with a magnetic stir bar and charged with **1a** (0.3 mmol), $NaNO_2$ (0.03 mmol, 10 mol %), and DMAP (0.12 mmol, 40 mol %), was evacuated and backfilled with oxygen (this process was repeated three times). After toluene (1.5 mL) was added, HNO_3 was added. Then, **2a** (0.45 mmol, 1.5 equiv) in toluene (1.5 mL) were added to the test tube. After the reaction mixture was stirred at room temperature for 48 h, the reaction mixture was diluted by adding CH_2Cl_2 and washed with a saturated aqueous solution of Na_2CO_3. Two layers were separated, and the aqueous layer was extracted with CH_2Cl_2. The combined organic layer was dried over $MgSO_4$, filtered, and concentrated on a rotary evaporator. The residue was purified by column chromatography (EtOAc/Hx = 1:10) to give 1,3,4-oxadiazine **3a**.

4. Optimization of one-pot synthesis of 1,3,4-oxadiazine

One 10 mL flame-dried test tube (Tube A), which was equipped with a magnetic stir bar and charged with *N'*-phenylbenzohydrazide (**1a**) (0.3 mmol) and catalyst, was evacuated and backfilled with oxygen (this process was repeated three times). After solvent (1.5 mL) was added, the reaction mixture was stirred for 2 h. The other 10 mL flame-dried test tube (Tube B), which was equipped with a magnetic stir bar, was evacuated and backfilled with nitrogen (this process was repeated three times). Benzyl allenoate (**2a**) (1.5 eq, 0.45 mmol) in solvent (0.5 mL) was added to Tube B. Then, the reaction mixture in Tube A was added to Tube B. By using the solvent (0.5 mL), the Tube A was washed, and the solution was transferred to Tube B using a syringe. After the combined mixture in Tube B was stirred at room temperature for 0.5 h, DMAP (0.12 mmol, 40 mol %) in solvent (0.5 mL) was added. After 48 h, the reaction mixture in Tube B was diluted by adding CH_2Cl_2 and washed with a saturated aqueous solution of Na_2CO_3. Two layers were separated, and the aqueous layer was extracted with CH_2Cl_2. The combined

organic layer was dried over $MgSO_4$, filtered, and concentrated on a rotary evaporator. The residue was purified by column chromatography (EtOAc/Hx = 1:10) to give 1,3,4-oxadiazine **3a**.

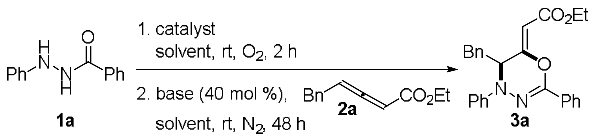

Entry	Catalyst (mol %)	Base	Solvent	Yield (%)[a]
1	CuCl (10) / DMAP (20)	DMAP	toluene	24
2	Fe(Pc) (10)	DMAP	Toluene	30
3	Mn(Pc) (10)	DMAP	Toluene	13
4	$NaNO_2$ (10) / HNO_3 (20)	DMAP	Toluene	75
5	$NaNO_2$ (10) / HNO_3 (20)	pyridine	Toluene	13
6	$NaNO_2$ (10) / HNO_3 (20)	DBU	Toluene	0
7	$NaNO_2$ (10) / HNO_3 (20)	PPh_3	Toluene	0
8	$NaNO_2$ (10) / HNO_3 (20)	DMAP	DMSO	24
9	$NaNO_2$ (10) / HNO_3 (20)	DMAP	DMF	23
10	$NaNO_2$ (10) / HNO_3 (20)	DMAP	CH_3CN	20
11	$NaNO_2$ (10) / HNO_3 (20)	DMAP	CH_2Cl_2	46
12	$NaNO_2$ (10) / HNO_3 (20)	DMAP	1,4-dioxane	45
13	$NaNO_2$ (10) / HNO_3 (20)	DMAP	EtOH	25
14[b]	$NaNO_2$ (10) / HNO_3 (20)	DMAP	Toluene	76
15[c]	$NaNO_2$ (10) / HNO_3 (20)	DMAP	Toluene	76
16[d]	$NaNO_2$ (10) / HNO_3 (20)	DMAP	Toluene	67
17[c]	$NaNO_2$ (10) / HNO_3 (10)	DMAP	Toluene	49
18[c]	$NaNO_2$ (10) / HNO_3 (30)	DMAP	Toluene	74
19[c]	$NaNO_2$ (5) / HNO_3 (10)	DMAP	Toluene	45
20[c,e]	$NaNO_2$ (10) / HNO_3 (20)	DMAP	Toluene	31

[a] Isolated yield. [b] The use of 30 mol % of DMAP. [c] The use of 1.2 equiv of **2a** and 30 mol % of DMAP. [d] The use of 1.0 equiv of **2a** and 30 mol % DMAP. [e] Under air.

5. General procedure for one-pot synthesis of 1,3,4-oxadiazines

One 10 mL flame-dried test tube (Tube A), which was equipped with a magnetic stir bar and charged with acylhydrazide (0.3 mmol) and $NaNO_2$ (0.03 mmol, 10 mol %), was evacuated and backfilled with oxygen (this process was repeated three times). After toluene (1.0 mL), HNO_3 (0.06 mmol, 20 mol %), and additional toluene (0.5 mL) were added in sequence, the reaction mixture was stirred for 2 h. The

other 10 mL flame-dried test tube (Tube B), which was equipped with a magnetic stir bar, was evacuated and backfilled with nitrogen (this process was repeated three times). Allenoate (0.36 mmol, 1.2 equiv) in toluene (0.5 mL) was added to Tube B. Then, the reaction mixture in Tube A was added to Tube B using a syringe. By using toluene (0.5 mL), the Tube A was washed, and the solution was transferred to Tube B. After the combined mixture in Tube B was stirred at room temperature for 0.5 h, DMAP (0.09 mmol, 30 mol %) in toluene (0.5 mL) was added. After 48 h, the reaction mixture in Tube B was diluted by adding CH_2Cl_2 and washed with a saturated aqueous solution of Na_2CO_3. Two layers were separated, and the aqueous layer was extracted with CH_2Cl_2. The combined organic layer was dried over $MgSO_4$, filtered, and concentrated on a rotary evaporator. The residue was purified by column chromatography to give 1,3,4-oxadiazines.

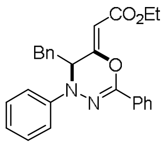

(*Z*)-Ethyl 2-(5-benzyl-2,4-diphenyl-4*H*-1,3,4-oxadiazin-6(5*H*)-ylidene)acetate (3a)[4]; yellow solid, EtOAc/Hx = 1:10, ^{1}H NMR (400 MHz, $CDCl_3$) δ 8.15 – 8.07 (m, 2H), 7.45 (dd, *J* = 5.0, 2.4 Hz, 3H), 7.35 (dd, *J* = 8.8, 7.2 Hz, 2H), 7.29 – 7.25 (m, 4H), 7.19 – 7.15 (m, 3H), 6.96 (t, *J* = 7.2 Hz, 1H), 4.75 (s, 1H), 4.69 (dd, *J* = 9.5, 4.8 Hz, 1H), 4.27 – 4.08 (m, 2H), 3.03 – 2.84 (m, 2H), 1.29 (t, *J* = 7.1 Hz, 3H); ^{13}C NMR (101 MHz, $CDCl_3$) δ 164.5, 153.1, 144.6, 142.0, 136.4, 130.1, 130.0, 129.6, 129.5, 128.8, 128.6, 127.2, 125.8, 121.1, 114.2, 98.4, 60.2, 56.3, 34.1, 14.4.

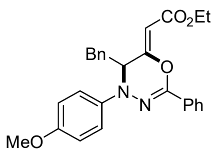

(*Z*)-Ethyl 2-(5-benzyl-4-(4-methoxyphenyl)-2-phenyl-4*H*-1,3,4-oxadiazin-6(5*H*)-ylidene)acetate (3b); yellow oil, EtOAc/Hx = 1:10, ^{1}H NMR (400 MHz, $CDCl_3$) δ 8.11 (d, *J* = 6.9 Hz, 2H), 7.45 (d, *J* = 6.4 Hz, 3H), 7.29 – 7.14 (m, 8H), 6.92 (d, *J* = 8.8 Hz, 2H), 4.72 (s, 1H), 4.59 (dd, *J* = 9.2, 4.6 Hz, 1H), 4.25 – 4.15 (m, 2H), 3.82 (s, 3H), 2.98 – 2.84 (m, 2H), 1.30 (t, *J* = 7.1 Hz, 3H); ^{13}C NMR (75 MHz, $CDCl_3$) δ 164.5, 154.6, 153.3, 141.8, 138.8, 136.5, 130.1, 129.8, 129.5, 128.7, 128.5, 127.0, 125.6, 116.1, 114.7, 98.0, 60.1, 57.4, 55.7, 33.5, 14.3; HRMS (EI) m/z calcd. For $C_{27}H_{26}N_2O_4$ $[M]^+$: 442.1893, found 442.1895.

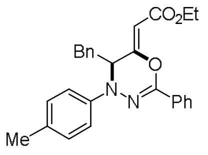

(*Z*)-Ethyl 2-(5-benzyl-2-phenyl-4-(*p*-tolyl)-4*H*-1,3,4-oxadiazin-6(5*H*)-ylidene)acetate (3c)[4]; yellow oil, EtOAc/Hx = 1:10, ^{1}H NMR (300 MHz, $CDCl_3$) δ 8.12 (dd, *J* = 7.7, 2.1 Hz, 2H), 7.57 – 7.37 (m, 3H), 7.28 – 7.07 (m, 9H), 4.72 (s, 1H), 4.64 (dd, *J* = 9.5, 4.7 Hz, 1H), 4.23 – 4.14 (m, 2H), 2.98 – 2.83 (m, 2H), 2.31 (s, 3H), 1.28 (t, *J* = 7.2 Hz, 3H); ^{13}C NMR (75 MHz, $CDCl_3$) δ 164.4, 153.0, 142.3, 141.6, 136.4, 130.4, 130.1, 129.9, 129.7, 129.4, 128.6, 128.4, 127.0, 125.6, 114.3, 98.1, 60.0, 56.5, 33.6, 20.5, 14.3.

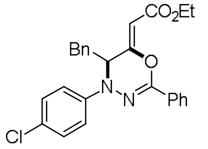

(*Z*)-Ethyl 2-(5-benzyl-4-(4-chlorophenyl)-2-phenyl-4*H*-1,3,4-oxadiazin-6(5*H*)-ylidene)acetate (3d)[4]; yellow oil, EtOAc/Hx = 1:10, ^{1}H NMR (400 MHz, $CDCl_3$) δ 8.16 – 8.07 (m, 2H), 7.49 – 7.44 (m, 3H), 7.28 (d, *J* = 4.6 Hz, 3H), 7.23 (d, *J* = 7.1 Hz, 2H), 7.16 (t, *J* = 7.3 Hz, 4H), 4.80 (s, 1H), 4.65 (dd, *J* = 8.9, 5.4 Hz, 1H), 4.26 – 4.16 (m, 2H), 2.99 – 2.86 (m, 2H), 1.31 (t, *J* = 7.1 Hz, 3H); ^{13}C NMR (75 MHz, $CDCl_3$) δ 164.3, 152.7, 143.2, 142.3, 136.0, 130.1, 129.8, 129.5, 129.2, 128.8, 128.5, 127.2, 125.8, 125.8, 115.2, 98.5, 60.2, 56.2, 34.1, 14.3.

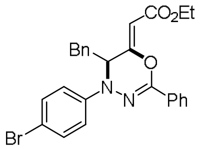

(*Z*)-Ethyl 2-(5-benzyl-4-(4-bromophenyl)-2-phenyl-4*H*-1,3,4-oxadiazin-6(5*H*)-ylidene)acetate (3e)[4]; yellow oil, EtOAc/Hx = 1:10, ^{1}H NMR (400 MHz,) δ 8.14 – 8.07 (m, 2H), 7.49 – 7.42 (m, 3H), 7.42 – 7.37 (m, 2H), 7.30 – 7.25 (m, 1H), 7.25 – 7.18 (m, 2H), 7.15 – 7.08 (m, 4H), 4.80 (s, 1H), 4.63 (dd, J = 9.0, 5.3 Hz, 1H), 4.27 – 4.14 (m, 2H), 3.01 – 2.81 (m, 2H), 1.29 (t, J = 7.1 Hz, 3H); ^{13}C NMR (75 MHz, $CDCl_3$) δ 164.3, 152.7, 143.6, 142.3, 135.9, 132.1, 130.1, 129.7, 129.4, 128.7, 128.5, 127.2, 125.8, 115.6, 113.1, 98.5, 60.2, 56.1, 34.1, 14.3.

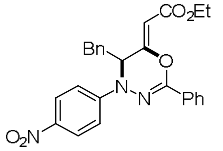

(*Z*)-Ethyl 2-(5-benzyl-4-(4-nitrophenyl)-2-phenyl-4*H*-1,3,4-oxadiazin-6(5*H*)-ylidene)acetate (3f); yellow oil, EtOAc/Hx = 1 : 10, ^{1}H NMR (400 MHz, $CDCl_3$) δ 8.06 – 7.96 (m, 4H), 7.49 (d, *J* = 7.0 Hz, 3H), 7.20 – 7.04 (m, 7H), 6.65 (dd, *J* = 8.2, 5.8 Hz, 1H), 5.73 (s, 1H), 4.22 – 4.14 (m, 2H), 3.10 – 2.99 (m, 2H), 1.29 (t, *J* = 7.1 Hz, 3H); ^{13}C NMR (101 MHz, $CDCl_3$) δ 166.2, 158.3, 149.6, 144.2, 140.0,

135.2, 130.9, 129.9, 129.1, 128.6, 128.5, 127.3, 125.8, 125.5, 112.3, 99.0, 60.6, 50.6, 35.2, 14.3; HRMS (EI) m/z calcd. For $C_{26}H_{23}N_3O_5$ $[M]^+$: 457.1638, found 457.1635.

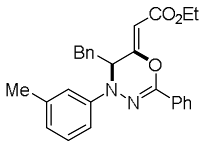

(*Z*)-Ethyl 2-(5-benzyl-2-phenyl-4-(*m*-tolyl)-4H-1,3,4-oxadiazin-6(5*H*)-ylidene)acetate (3g); yellow solid, EtOAc/Hx = 1:10, mp 102–103 °C, ^{1}H NMR (400 MHz, $CDCl_3$) δ 8.18 – 8.04 (m, 2H), 7.48 – 7.43 (m, 3H), 7.30 – 7.25 (m, 2H), 7.24 – 7.17 (m, 3H), 7.15 (s, 1H), 7.10 (s, 1H), 7.03 (dd, *J* = 8.2, 2.2 Hz, 1H), 6.82 – 6.76 (m, 1H), 4.75 (s, 1H), 4.67 (dd, *J* = 9.4, 4.8 Hz, 1H), 4.35 – 4.14 (m, 2H), 3.11 – 2.84 (m, 2H), 2.37 (s, 3H), 1.41 – 1.23 (m, 3H); ^{13}C NMR (75 MHz, $CDCl_3$) δ 164.5, 153.0, 144.6, 141.8, 139.3, 136.4, 130.1, 129.9, 129.5, 129.2, 128.7, 128.5, 127.1, 125.7, 121.9, 115.0, 111.2, 98.2, 60.1, 56.3, 34.0, 21.9, 14.4; HRMS (EI) m/z calcd. For $C_{27}H_{26}N_2O_3$ $[M]^+$: 426.1943, found 426.1941.

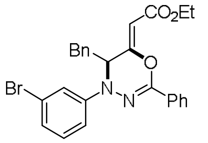

(*Z*)-Ethyl 2-(5-benzyl-4-(3-bromophenyl)-2-phenyl-4*H*-1,3,4-oxadiazin-6(5*H*)-ylidene)acetate (3h); yellow oil, EtOAc/Hx = 1:10, ^{1}H NMR (400 MHz, $CDCl_3$) δ 8.12 (d, *J* = 4.2 Hz, 2H), 7.47 (s, 3H), 7.43 (s, 1H), 7.28 (s, 2H), 7.23 (d, *J* = 7.4 Hz, 1H), 7.16 (d, *J* = 7.3 Hz, 3H), 7.10 – 7.04 (m, 2H), 4.83 (s, 1H), 4.68 – 4.63 (m, 1H), 4.22 (d, *J* = 6.4 Hz, 2H), 3.01 – 2.89 (m, 2H), 1.31 (t, *J* = 7.1 Hz, 3H); ^{13}C NMR (101 MHz, $CDCl_3$) δ 164.3, 152.6, 145.7, 142.4, 135.8, 130.5, 130.2, 129.6, 129.5, 128.7, 128.5, 127.3, 125.8, 123.5, 123.4, 117.1, 112.0, 98.6, 60.2, 55.9, 34.3, 14.3; HRMS (EI) m/z calcd. For $C_{26}H_{23}BrN_2O_3$ $[M]^+$: 490.0892, found 490.0895.

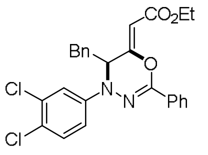

(*Z*)-Ethyl 2-(5-benzyl-4-(3,4-dichlorophenyl)-2-phenyl-4*H*-1,3,4-oxadiazin-6(5*H*)-ylidene)acetate (3i)[4]; yellow oil, EtOAc/Hx = 1:10, ^{1}H NMR (400 MHz, $CDCl_3$) δ 8.14 – 8.10 (m, 2H), 7.50 – 7.47 (m, 3H), 7.34 – 7.27 (m, 4H), 7.23 (d, *J* = 6.9 Hz, 1H), 7.14 (d, *J* = 6.8 Hz, 2H), 6.97 (dd, *J* = 9.0, 2.6 Hz, 1H), 4.87 (s, 1H), 4.63 (dd, *J* = 8.3, 5.9 Hz, 1H), 4.24 – 4.18 (m, 2H), 2.95 (td, *J* = 16.4, 15.0, 7.1 Hz, 2H), 1.32 (d, *J* = 7.1 Hz, 3H); ^{13}C NMR (101 MHz, $CDCl_3$) δ 164.2, 152.5, 144.1, 142.8, 135.7, 133.2, 130.6, 130.4, 129.5, 129.5, 128.8, 128.6, 127.4, 125.9, 123.6, 115.8, 113.0, 98.8, 60.3, 56.1, 34.5, 14.3.

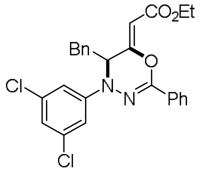

(*Z*)-Ethyl 2-(5-benzyl-4-(3,5-dichlorophenyl)-2-phenyl-4*H*-1,3,4-oxadiazin-6(5*H*)-ylidene)acetate (3j)[4]; yellow oil, EtOAc/Hx = 1:10, ^{1}H NMR (400 MHz, $CDCl_3$) δ 8.13 (s, 2H), 7.49 (s, 3H), 7.29 (d, *J* = 7.0 Hz, 2H), 7.23 (d, *J* = 6.6 Hz, 1H), 7.15 (d, *J* = 7.0 Hz, 2H), 7.03 (s, 2H), 6.87 (s, 1H), 4.90 (s, 1H), 4.64 – 4.59 (m, 1H), 4.25 – 4.19 (m, 2H), 2.95 (dd, *J* = 18.2, 7.1 Hz, 2H), 1.32 (t, *J* = 7.1 Hz, 3H); ^{13}C NMR (101 MHz, $CDCl_3$) δ 164.2, 152.4, 146.1, 135.6, 135.4, 130.5, 129.5, 129.3, 128.8, 128.8, 128.6, 127.4, 126.0, 120.2, 112.0, 98.9, 60.3, 55.8, 34.7, 14.3.

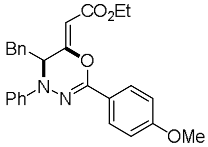

(*Z*)-Ethyl 2-(5-benzyl-2-(4-methoxyphenyl)-4-phenyl-4*H*-1,3,4-oxadiazin-6(5*H*)-ylidene)acetate (3m)[4]; yellow solid, EtOAc/Hx = 1:10, ^{1}H NMR (400 MHz, $CDCl_3$) δ 8.11 – 8.01 (m, 2H), 7.34 (dd, J = 8.8, 7.1 Hz, 2H), 7.30 – 7.25 (m, 4H), 7.23 – 7.15 (m, 3H), 7.01 – 6.91 (m, 3H), 4.73 (s, 1H), 4.67 (dd, J = 9.6, 4.8 Hz, 1H), 4.24 – 4.14 (m, 2H), 3.87 (s, 3H), 3.04 – 2.83 (m, 2H), 1.29 (t, J = 7.2 Hz, 3H); ^{13}C NMR (101 MHz, $CDCl_3$) δ 153.1, 144.6, 142.2, 140.2, 136.3, 129.5, 129.4, 129.2, 128.7, 127.2, 127.1, 125.7, 120.8, 118.2, 114.0, 98.2, 60.1, 56.2, 33.8, 21.6, 14.4.

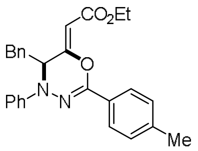

(*Z*)-Ethyl 2-(5-benzyl-4-phenyl-2-(*p*-tolyl)-4*H*-1,3,4-oxadiazin-6(5*H*)-ylidene)acetate (3n)[4]; yellow oil, EtOAc/Hx = 1:10, ^{1}H NMR (300 MHz, $CDCl_3$) δ 8.05 – 7.99 (m, 2H), 7.34 (dd, *J* = 8.8, 7.0 Hz, 2H), 7.30 – 7.19 (m, 7H), 7.15 (dd, *J* = 8.0, 1.6 Hz, 2H), 6.99 – 6.91 (m, 1H), 4.73 (s, 1H), 4.67 (dd, *J* = 9.4, 4.9 Hz, 1H), 4.24 – 4.13 (m, 2H), 3.03 – 2.82 (m, 2H), 2.41 (s, 3H), 1.29 (t, *J* = 7.1 Hz, 3H); ^{13}C NMR (75 MHz, $CDCl_3$) δ 164.5, 153.1, 144.6, 142.2, 140.1, 136.3, 129.5, 129.3, 129.2, 128.7, 127.2, 127.1, 125.7, 120.8, 114.0, 98.2, 60.1, 56.2, 33.9, 21.5, 14.3.

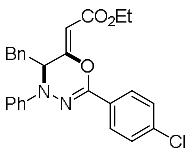

(*Z*)-Ethyl 2-(5-benzyl-2-(4-chlorophenyl)-4-phenyl-4*H*-1,3,4-oxadiazin-6(5*H*)-ylidene)acetate (3o)[4]; yellow solid, EtOAc/Hx = 1:10, ^{1}H NMR (300 MHz, $CDCl_3$) δ 8.14 – 8.02 (m, 2H), 7.49 – 7.28 (m, 8H), 7.26 – 7.17 (m, 3H), 7.01 (t, *J* = 7.2 Hz, 1H), 4.82 (s, 1H), 4.74 (dd, *J* = 9.3, 4.8 Hz, 1H), 4.27 – 4.18 (m, 2H), 3.01 – 2.85 (m, 2H), 1.34 (d, *J* = 7.1 Hz, 3H); ^{13}C NMR (75 MHz, $CDCl_3$) δ 164.4, 152.8, 144.3, 141.1, 136.1, 135.9, 129.5, 129.4, 128.7, 128.7, 128.6, 127.2, 127.0, 121.2, 114.1, 98.5, 60.2, 56.1, 34.2, 14.3.

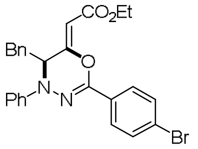

(*Z*)-Ethyl 2-(5-benzyl-2-(4-bromophenyl)-4-phenyl-4*H*-1,3,4-oxadiazin-6(5*H*)-ylidene)acetate (3p) [4]; yellow solid, EtOAc/Hx = 1:10, ^{1}H NMR (300 MHz, $CDCl_3$) δ 7.99 – 7.91 (m, 2H), 7.60 – 7.54 (m, 2H), 7.35 (td, *J* = 7.2, 2.0 Hz, 2H), 7.30 – 7.20 (m, 5H), 7.17 – 7.12 (m, 2H), 6.97 (t, *J* = 7.2 Hz, 1H), 4.78 (s, 1H), 4.70 (dd, *J* = 9.3, 4.8 Hz, 1H), 4.22 – 4.16 (m, 2H), 3.02 – 2.84 (m, 2H), 1.29 (t, *J* = 7.1 Hz, 3H); ^{13}C NMR (75 MHz, $CDCl_3$) δ 164.3, 152.7, 144.3, 141.1, 136.1, 131.7, 129.5, 129.4, 129.0, 128.7, 127.2, 127.2, 124.2, 121.1, 114.1, 98.6, 60.2, 56.1, 34.2, 14.3.

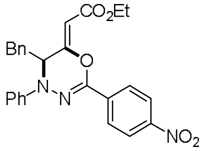

(*Z*)-Ethyl 2-(5-benzyl-2-(4-nitrophenyl)-4-phenyl-4*H*-1,3,4-oxadiazin-6(5*H*)-ylidene)acetate (3q); orange solid, EtOAc/Hx =1 : 10, mp 75–76 °C, ^{1}H NMR (400 MHz, $CDCl_3$) δ 8.31 – 8.19 (m, 4H), 7.43 – 7.34 (m, 2H), 7.34 – 7.24 (m, 4H), 7.23 – 7.13 (m, 3H), 7.03 (t, *J* = 7.2 Hz, 1H), 4.88 (s, 1H), 4.77 (dd, *J* = 9.1, 4.7 Hz, 1H), 4.27 – 4.17 (m, 2H), 3.07 – 2.89 (m, 2H), 1.31 (t, *J* = 7.1 Hz, 3H); ^{13}C NMR (101 MHz, $CDCl_3$) δ 164.2, 152.1, 148.2, 143.8, 139.7, 135.9, 135.7, 129.5, 129.5, 128.7, 127.3, 126.1, 123.8, 121.8, 114.2, 99.0, 60.3, 56.0, 34.7, 14.3; HRMS (EI) m/z calcd. For $C_{26}H_{23}N_3O_5$ $[M]^+$: 457.1638, found 457.1639.

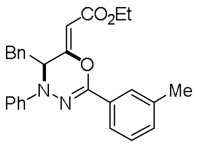

(*Z*)-Ethyl 2-(5-benzyl-4-phenyl-2-(*m*-tolyl)-4*H*-1,3,4-oxadiazin-6(5*H*)-ylidene)acetate (3r); yellow oil, EtOAc/Hx = 1:10, ^{1}H NMR (300 MHz, $CDCl_3$) δ 7.94 (d, *J* = 7.5 Hz, 2H), 7.39 – 7.32 (m, 3H), 7.31 – 7.20 (m, 6H), 7.20 – 7.14 (m, 2H), 7.00 – 6.91 (m, 1H), 4.74 (s, 1H), 4.68 (dd, *J* = 9.5, 4.9 Hz, 1H), 4.20 (dd, *J* = 7.1, 5.6 Hz, 2H), 3.01 – 2.84 (m, 2H), 2.45 (s, 3H), 1.31 (t, *J* = 7.1 Hz, 3H); ^{13}C NMR

(75 MHz, $CDCl_3$) δ 164.6, 153.0, 144.6, 142.2, 138.2, 136.4, 130.9, 130.0, 129.5, 129.4, 128.8, 128.5, 127.2, 126.2, 123.1, 121.0, 114.2, 98.4, 60.2, 56.3, 33.9, 21.6, 14.4; HRMS (EI) m/z calcd. For $C_{27}H_{26}N_2O_3$ $[M]^+$: 426.1943, found 426.1939.

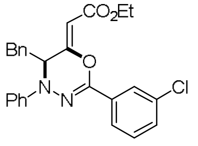

(*Z*)-Ethyl 2-(5-benzyl-2-(3-chlorophenyl)-4-phenyl-4*H*-1,3,4-oxadiazin-6(5*H*)-ylidene)acetate (3s); yellow oil, EtOAc/Hx = 1:10, ^{1}H NMR (300 MHz, $CDCl_3$) δ 8.07 (q, J = 1.3 Hz, 1H), 7.99 (dd, J = 5.5, 1.8 Hz, 1H), 7.42 – 7.33 (m, 4H), 7.32 – 7.21 (m, 5H), 7.18 – 7.13 (m, 2H), 6.99 (d, J = 7.2 Hz, 1H), 4.78 (s, 1H), 4.71 (dd, J = 9.3, 4.8 Hz, 1H), 4.21 (dd, J = 7.1, 5.0 Hz, 2H), 3.02 – 2.84 (m, 2H), 1.32 (t, J = 7.1 Hz, 3H); ^{13}C NMR (75 MHz, $CDCl_3$) δ 164.4, 152.4, 144.2, 140.6, 136.1, 134.6, 131.8, 129.8, 129.8, 129.5, 129.4, 128.7, 127.2, 125.6, 123.8, 121.3, 114.1, 98.8, 60.3, 56.2, 34.1, 14.4; HRMS (EI) m/z calcd. For $C_{26}H_{23}ClN_2O_3$ $[M]^+$: 446.1397, found 446.1400.

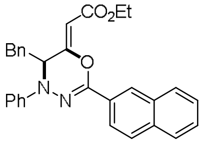

(*Z*)-Ethyl 2-(5-benzyl-2-(naphthalen-2-yl)-4-phenyl-4*H*-1,3,4-oxadiazin-6(5*H*)-ylidene)acetate (3t); yellow solid, EtOAc/Hx = 1:10, mp 66–67 °C, ^{1}H NMR (400 MHz, $CDCl_3$) δ 8.61 (s, 1H), 8.25 (d, J = 8.6 Hz, 1H), 8.05 – 7.98 (m, 1H), 7.96 – 7.85 (m, 2H), 7.63 – 7.52 (m, 2H), 7.46 – 7.18 (m, 9H), 7.02 (t, J = 6.8 Hz, 1H), 4.82 (s, 1H), 4.76 (dd, J = 9.3, 4.6 Hz, 1H), 4.28 (dd, J = 12.4, 6.9 Hz, 2H), 3.07 – 2.94 (m, 2H), 1.39 (t, J = 7.1 Hz, 3H); ^{13}C NMR (75 MHz, $CDCl_3$) δ 164.6, 152.9, 144.5, 142.1, 136.3, 134.1, 133.1, 129.5, 129.4, 128.8, 128.7, 128.2, 127.8, 127.4, 127.2, 126.9, 126.5, 125.4, 123.0, 121.1, 114.1, 98.5, 60.2, 56.3, 34.1, 14.5; HRMS (EI) calcd, for $C_{30}H_{26}N_2O_3$ $[M]^+$: 462.1943, found: 462.1947.

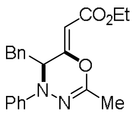

(*Z*)-Ethyl 2-(5-benzyl-2-methyl-4-phenyl-4*H*-1,3,4-oxadiazin-6(5*H*)-ylidene)acetate (3u)[4]; yellow oil, EtOAc/Hx = 1:4, ^{1}H NMR (300 MHz, $CDCl_3$) δ 7.36 – 7.27 (m, 5H), 7.19 – 7.15 (m, 4H), 7.01 – 6.88 (m, 1H), 4.69 (s, 1H), 4.57 (dd, J = 8.9, 5.0 Hz, 1H), 4.20 – 4.12 (m, 2H), 2.95 – 2.83 (m, 2H), 2.27 (s, 3H), 1.28 (d, J = 7.1 Hz, 3H); ^{13}C NMR (75 MHz, $CDCl_3$) δ 164.3, 153.6, 144.7, 143.3, 136.4, 129.5, 129.3, 128.7, 127.0, 120.6, 113.8, 97.7, 60.0, 56.0, 33.4, 17.9, 14.2.

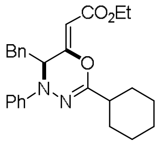

(*Z*)-Ethyl 2-(5-benzyl-2-cyclohexyl-4-phenyl-4*H*-1,3,4-oxadiazin-6(5*H*)-ylidene)acetate (3v); yellow oil, EtOAc/Hx = 1:10, ^{1}H NMR (300 MHz, $CDCl_3$) δ 7.33 – 7.26 (m, 3H), 7.26 – 7.21 (m, 2H), 7.19 – 7.11 (m, 4H), 6.91 (d, *J* = 7.2 Hz, 1H), 4.61 (s, 1H), 4.54 (dd, *J* = 9.3, 5.0 Hz, 1H), 4.25 – 4.11 (m, 2H), 2.90 – 2.74 (m, 2H), 2.60 – 2.44 (m, 1H), 2.14 – 2.05 (m, 2H), 1.85 (d, *J* = 10.6 Hz, 2H), 1.67 (dd, *J* = 27.1, 13.4 Hz, 3H), 1.41 – 1.29 (m, 3H), 1.28 – 1.24 (m, 3H); ^{13}C NMR (75 MHz, $CDCl_3$) δ 164.4, 153.8, 149.1, 144.9, 136.4, 129.5, 129.3, 128.6, 127.0, 120.4, 113.9, 97.6, 59.9, 56.1, 40.7, 32.8, 29.7, 25.9, 25.7, 14.3; HRMS (EI) m/z calcd. For $C_{26}H_{30}N_2O_3$ $[M]^+$: 418.2256, found 418.2254.

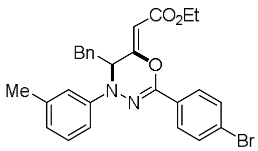

(*Z*)-Ethyl 2-(5-benzyl-2-(4-bromophenyl)-4-(*m*-tolyl)-4*H*-1,3,4-oxadiazin-6(5*H*)-ylidene)acetate (3w); yellow solid, EtOAc/Hx = 1:10, mp 95–96 °C, ^{1}H NMR (400 MHz, $CDCl_3$) δ 8.06 – 7.89 (m, 2H), 7.64 – 7.52 (m, 2H), 7.29 (d, *J* = 7.9 Hz, 2H), 7.23 (d, *J* = 7.8 Hz, 2H), 7.16 (d, *J* = 7.3 Hz, 2H), 7.09 (s, 1H), 7.04 (d, *J* = 8.3 Hz, 1H), 6.80 (d, *J* = 7.4 Hz, 1H), 4.78 (s, 1H), 4.69 (dd, *J* = 9.5, 4.7 Hz, 1H), 4.31 – 4.11 (m, 2H), 3.01 – 2.85 (m, 2H), 2.39 (s, 3H), 1.29 (dd, *J* = 7.9, 6.3 Hz, 3H); ^{13}C NMR (75 MHz, $CDCl_3$) δ 164.4, 152.8, 144.3, 141.0, 139.3, 136.2, 131.6, 129.5, 129.2, 129.0, 128.8, 128.7, 127.2, 124.2, 122.1, 115.0, 111.2, 98.4, 60.1, 56.2, 34.2, 21.9, 14.3; HRMS (EI) m/z calcd. For $C_{27}H_{25}BrN_2O_3$ $[M]^+$: 504.1049, found 504.1050.

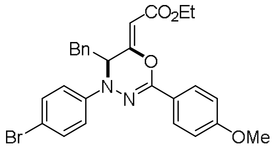

(*Z*)-Ethyl 2-(5-benzyl-4-(4-bromophenyl)-2-(4-methoxyphenyl)-4*H*-1,3,4-oxadiazin-6(5*H*)-ylidene)acetate (3x)[4]; yellow oil, EtOAc/Hx = 1:10, ^{1}H NMR (400 MHz, $CDCl_3$) δ 8.05 (d, *J* = 8.8 Hz, 2H), 7.40 (d, *J* = 8.9 Hz, 2H), 7.24 (dd, *J* = 15.7, 6.2 Hz, 3H), 7.12 (dd, *J* = 18.6, 7.7 Hz, 4H), 6.98 (d, *J* = 8.8 Hz, 2H), 4.78 (s, 1H), 4.61 (d, *J* = 3.5 Hz, 1H), 4.25 – 4.13 (m, 2H), 3.87 (s, 3H), 2.90 (dt, *J* = 22.5, 11.3 Hz, 2H), 1.30 (t, *J* = 7.1 Hz, 3H); ^{13}C NMR (101 MHz, $CDCl_3$) δ 164.4, 161.4, 153.1, 143.9, 142.6, 136.1, 132.1, 129.5, 128.8, 127.5, 127.3, 122.4, 115.6, 114.0, 112.9, 98.4, 60.2, 56.2, 55.5, 34.2, 14.4.

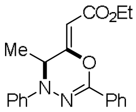

(*Z*)-Ethyl 2-(5-methyl-2,4-diphenyl-4*H*-1,3,4-oxadiazin-6(5*H*)-ylidene)acetate (3y)[4]; yellow oil, EtOAc/Hx = 1:10, ^{1}H NMR (300 MHz, $CDCl_3$) δ 8.12 – 8.09 (m, 2H), 7.48 – 7.40 (m, 3H), 7.39 – 7.25 (m, 4H), 7.00 – 6.93 (m, 1H), 4.71 (q, *J* = 6.7 Hz, 1H), 4.26 (q, *J* = 7.1 Hz, 2H), 1.38 – 1.29 (m, 6H); ^{13}C NMR (75 MHz, $CDCl_3$) δ 164.8, 156.4, 144.6, 141.3, 130.0, 129.8, 129.3, 128.4, 125.6, 121.0, 114.3, 96.1, 60.1, 49.1, 14.4, 13.3.

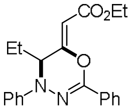

(*Z*)-Ethyl 2-(5-ethyl-2,4-diphenyl-4*H*-1,3,4-oxadiazin-6(5*H*)-ylidene)acetate (3z)[4]; yellow oil, EtOAc/Hx = 1:10, ^{1}H NMR (300 MHz, $CDCl_3$) δ 8.18 – 8.10 (m, 2H), 7.50 – 7.44 (m, 3H), 7.42 – 7.35 (m, 2H), 7.33 – 7.28 (m, 2H), 7.04 – 6.94 (m, 1H), 5.23 (s, 1H), 4.48 (s, 1H), 4.31 (dd, *J* = 7.1, 1.4 Hz, 2H), 1.83 – 1.71 (m, 2H), 1.40 (t, *J* = 7.1 Hz, 3H), 1.04 (t, *J* = 7.5 Hz, 3H); ^{13}C NMR (75 MHz, $CDCl_3$) δ 164.7, 153.9, 144.7, 141.5, 130.1, 129.8, 129.4, 128.5, 125.6, 120.8, 114.0, 97.8, 60.3, 55.5, 20.5, 14.4, 10.8.

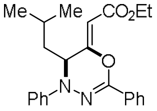

(*Z*)-Ethyl 2-(5-isobutyl-2,4-diphenyl-4*H*-1,3,4-oxadiazin-6(5*H*)-ylidene)acetate (3aa); yellow oil, EtOAc/Hx = 1:10, ^{1}H NMR (300 MHz, $CDCl_3$) δ 8.14 – 8.05 (m, 2H), 7.47 – 7.39 (m, 3H), 7.37 – 7.31 (m, 2H), 7.29 – 7.23 (m, 2H), 6.95 (tt, *J* = 7.2, 1.2 Hz, 1H), 5.20 (s, 1H), 4.61 (dd, *J* = 10.9, 3.9 Hz, 1H), 4.30 – 4.22 (m, 2H), 1.74 – 1.62 (m, 2H), 1.35 (t, *J* = 7.1 Hz, 4H), 1.04 (d, *J* = 6.4 Hz, 3H), 0.90 (d, *J* = 6.5 Hz, 3H); ^{13}C NMR (75 MHz, $CDCl_3$) δ 164.7, 154.0, 144.6, 141.4, 130.0, 129.8, 129.3, 128.4, 125.6, 120.8, 113.8, 97.5, 60.2, 52.2, 35.1, 24.7, 23.5, 21.4, 14.4; HRMS (EI) m/z calcd. For $C_{23}H_{26}N_2O_3$ $[M]^+$: 378.1943, found 378.1945.

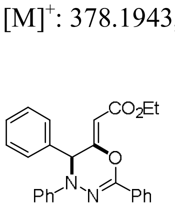

(*Z*)-Ethyl 2-(2,4,5-triphenyl-4*H*-1,3,4-oxadiazin-6(5*H*)-ylidene)acetate (3ab); yellow oil, EtOAc/Hx = 1:10, ^{1}H NMR (300 MHz, $CDCl_3$) δ 8.10 – 8.01 (m, 2H), 7.42 – 7.37 (m, 3H), 7.34 – 7.22 (m, 9H), 7.00 – 6.89 (m, 1H), 5.71 (s, 1H), 5.52 (s, 1H), 4.26 (q, *J* = 7.1 Hz, 2H), 1.35 (t, *J* = 7.2 Hz, 3H); ^{13}C NMR (126 MHz, $CDCl_3$) δ 164.7, 153.9, 144.9, 141.5, 134.6, 130.0, 129.8, 129.3, 129.1, 128.5, 128.4,

126.8, 125.6, 121.0, 113.9, 98.2, 60.4, 57.0, 14.4; HRMS (EI) m/z calcd. For $C_{25}H_{22}N_2O_3$ $[M]^+$: 398.1630, found 398.1634.

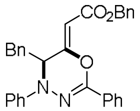

(*Z*)-Benzyl 2-(5-benzyl-2,4-diphenyl-4*H*-1,3,4-oxadiazin-6(5*H*)-ylidene)acetate (3ac); yellow oil, EtOAc/Hx =1:10, ^{1}H NMR (300 MHz, $CDCl_3$) δ 8.04 – 7.98 (m, 2H), 7.44 – 7.31 (m, 10H), 7.29 – 7.19 (m, 5H), 7.18 – 7.13 (m, 2H), 6.96 (tt, *J* = 7.1, 1.3 Hz, 1H), 5.23 – 5.11 (m, 2H), 4.80 (s, 1H), 4.70 (dd, *J* = 9.5, 4.9 Hz, 1H), 3.03 – 2.81 (m, 2H); ^{13}C NMR (75 MHz, $CDCl_3$) δ 164.2, 153.4, 144.5, 141.9, 136.2, 136.0, 129.8, 129.5, 129.4, 128.7, 128.6, 128.4, 128.3, 128.2, 127.1, 125.7, 121.0, 114.1, 98.0, 66.1, 56.2, 33.9.; HRMS (EI) m/z calcd. For $C_{31}H_{26}N_2O_3$ $[M]^+$: 474.1943, found 474.1944.

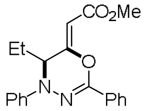

(*Z*)-Methyl 2-(5-ethyl-2,4-diphenyl-4*H*-1,3,4-oxadiazin-6(5*H*)-ylidene)acetate (3ad); yellow oil, EtOAc/Hx = 1:10, ^{1}H NMR (300 MHz, $CDCl_3$) δ 8.10 – 7.99 (m, 2H), 7.47 – 7.23 (m, 7H), 6.94 (tt, *J* = 7.0, 1.3 Hz, 1H), 5.20 (s, 1H), 4.43 (dd, *J* = 8.0, 6.6 Hz, 1H), 3.79 (s, 3H), 1.83 – 1.65 (m, 2H), 0.99 (t, *J* = 7.5 Hz, 3H); δ ^{13}C NMR (75 MHz, $CDCl_3$) δ 165.0, 154.1, 144.7, 141.4, 130.0, 129.7, 129.3, 128.4, 125.6, 120.7, 113.9, 97.2, 55.4, 51.4, 20.4, 10.7; HRMS (EI) m/z calcd. For $C_{20}H_{20}N_2O_3$ $[M]^+$: 336.1474, found 336.1471.

6. Scale-up process for one-pot synthesis of 3a

One 250 mL round-bottle flask (Flask A), which was equipped with a magnetic stir bar and charged with *N'*-phenylbenzohydrazide (**1a**) (5.0 mmol, 1.1 g) and $NaNO_2$ (0.5 mmol, 10 mol %), and backfilled with oxygen (this process was repeated three times). After toluene (15.0 mL), HNO_3 (1.0 mmol, 20 mol %), and additional toluene (5.0 mL) were added in sequence, the reaction mixture was stirred for 4h. The other 250 mL round-bottle flask (Flask B), which was equipped with a magnetic stir bar, was evacuated and backfilled with nitrogen (this process was repeated three times). Benzyl allenoate (**2a**) (6.0 mmol, 1.2 eq, 1.2 g) in toluene (5.0 mL) was added to Flask B. Then, the reaction mixture in Flask A was added to Flask B using a syringe. By using toluene (3.0 mL), Flask A was washed, and the solution was transferred to Flask B. After the combined mixture in Flask B was stirred at room temperature for 0.5 h, DMAP (1.5 mmol, 30 mol %) in toluene (7.0 mL) was added. After 48 h, the reaction mixture in Flask B was diluted by adding CH_2Cl_2 and washed with a saturated aqueous solution of Na_2CO_3. Two layers were separated, and the aqueous layer was extracted with CH_2Cl_2. The combined

organic layer was dried over $MgSO_4$, filtered, and concentrated on a rotary evaporator. The residue was purified by column chromatography to give 1,3,4-oxadiazines **3a**.

7. References

(1) Lim, J. H.; Baek, S. E.; Lad, B.; Kim, J. Synthesis of 2-Imino-1,3,4-oxadiazolines from Acylhydrazides and Isothiocyanates via Aerobic Oxidation and DMAP-Mediated Annulation Sequence. *ACS Omega* **2022**, *7*, 28148-28159.

(2) Fang, T.; Tan, O.; Ding, Z.; Liu, B.; Bin Xu, B. Pd-Catalyzed Oxidative Annulation of Hydrazides with Isocyanides: Synthesis of 2-Amino-1,3,4-oxadiazoles. *Org. Lett.* **2014**, *16*, 2342−2345.

(3) Xu, S.; Zhou, L.; Ma, R.; Song, H.; He, Z. Phosphane-Catalyzed [3+2] Annulation of Allenoates with Aldehydes: A Simple and Efficient Synthesis of 2-Alkylidenetetrahydrofurans. *Chem. Eur. J.* **2009**, *15*, 8698–8702.

(4) Zhang, Q.; Meng, L-.G.; Zhang, J.; Wang, L. DMAP-Catalyzed [2+4] Cycloadditions of Allenoates with *N*-Acyldiazenes: Direct Method to 1,3,4-Oxadiazine Derivatives. *Org. Lett.* **2015**, *17*, 3272–3275.

8. ^{1}H and ^{13}C NMR spectra

Ethyl 6-methylhepta-2,3-dienoate (for **3aa**)

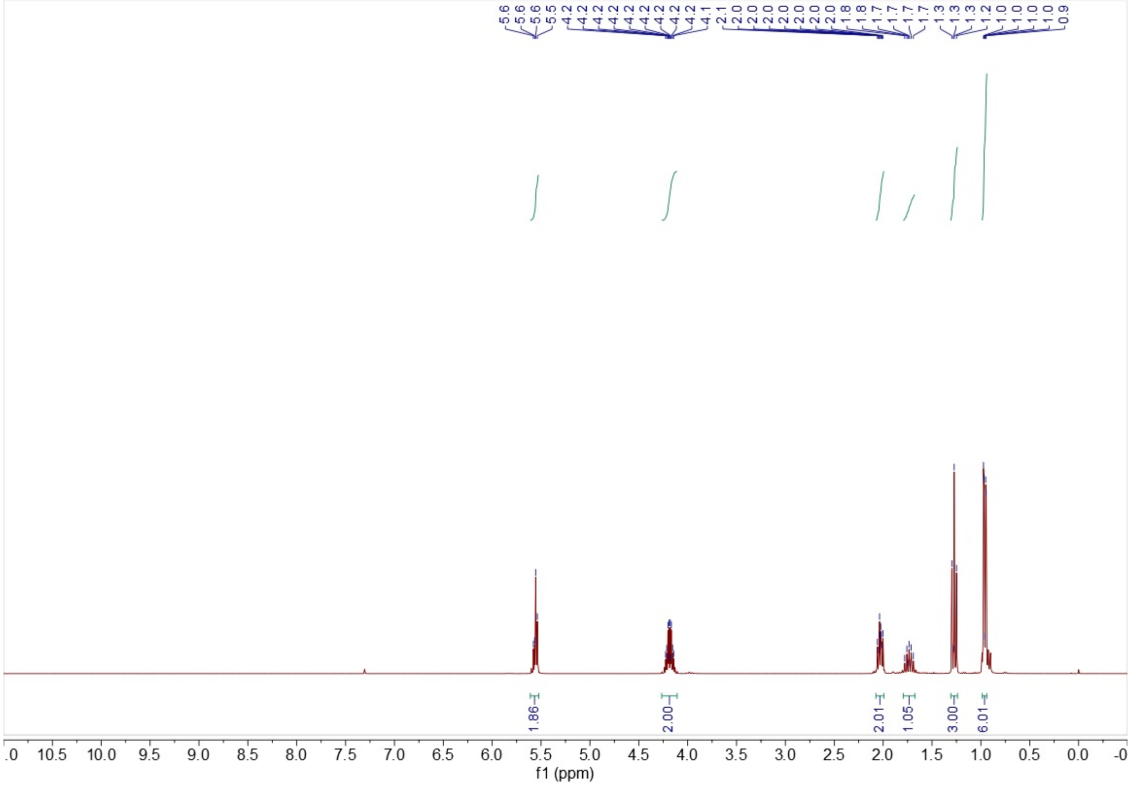

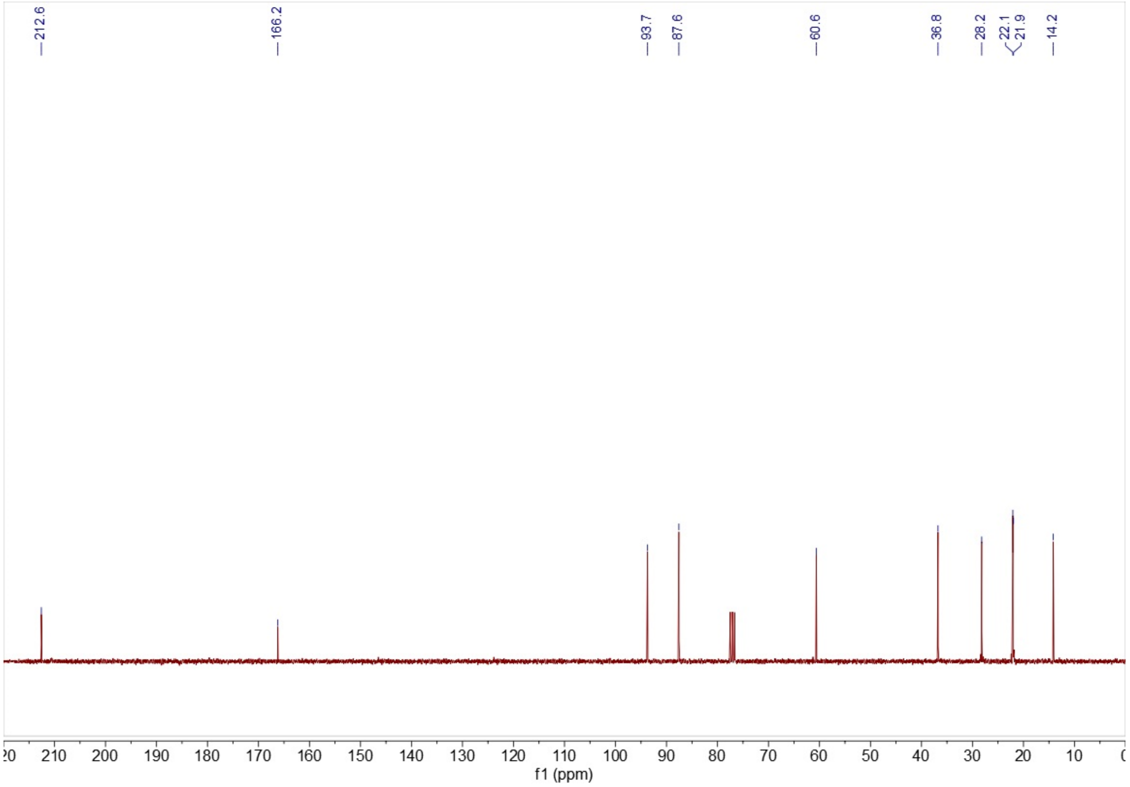

Methyl hexa-2,3-dienoate (for **3ad**)

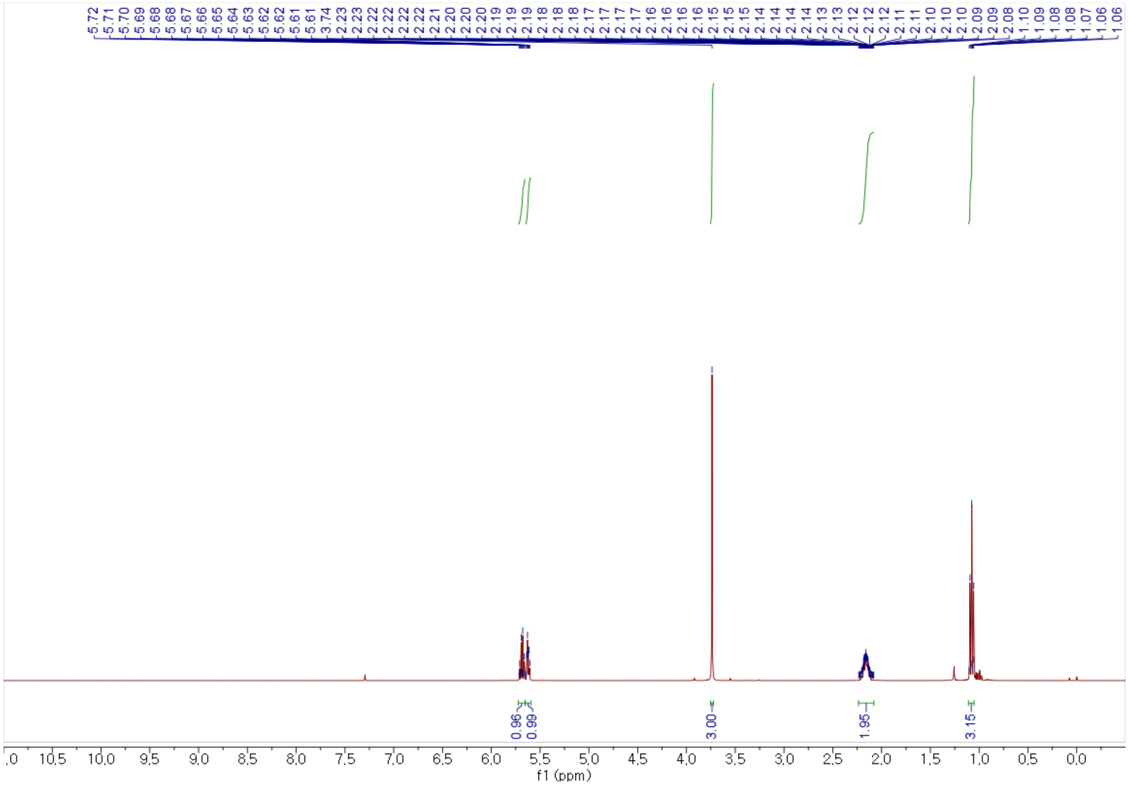

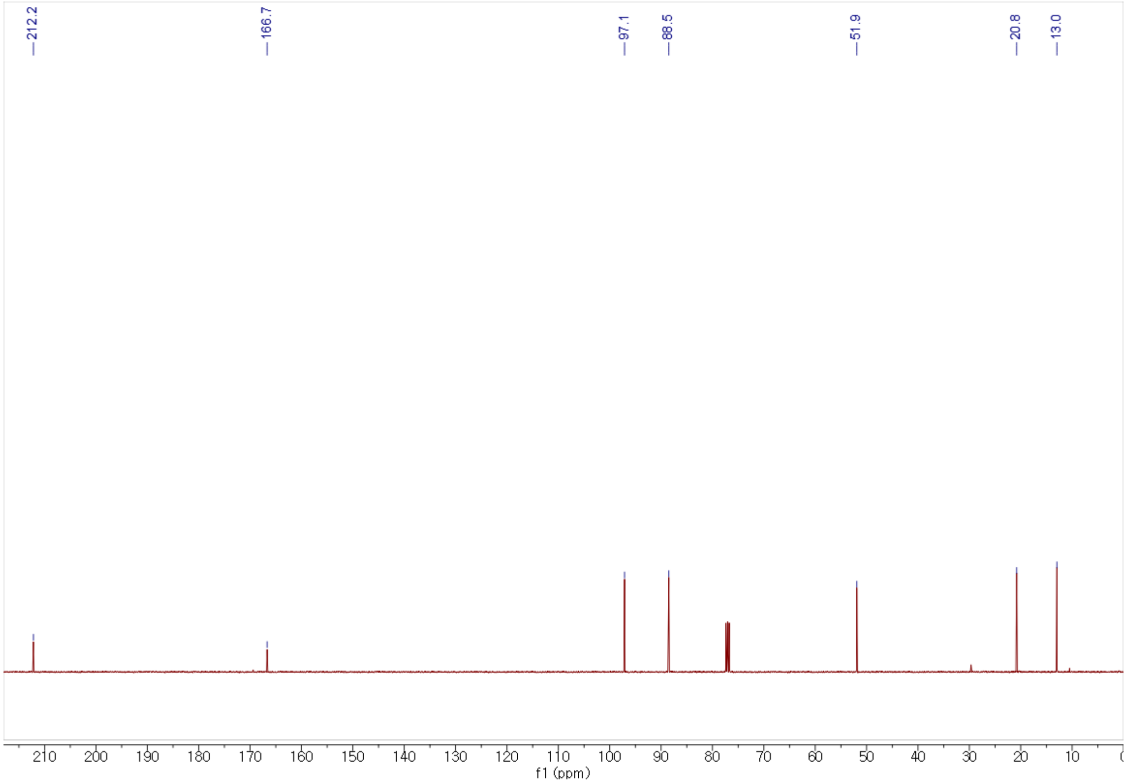

(*Z*)-Ethyl 2-(5-benzyl-2,4-diphenyl-4*H*-1,3,4-oxadiazin-6(5*H*)-ylidene)acetate (3a)

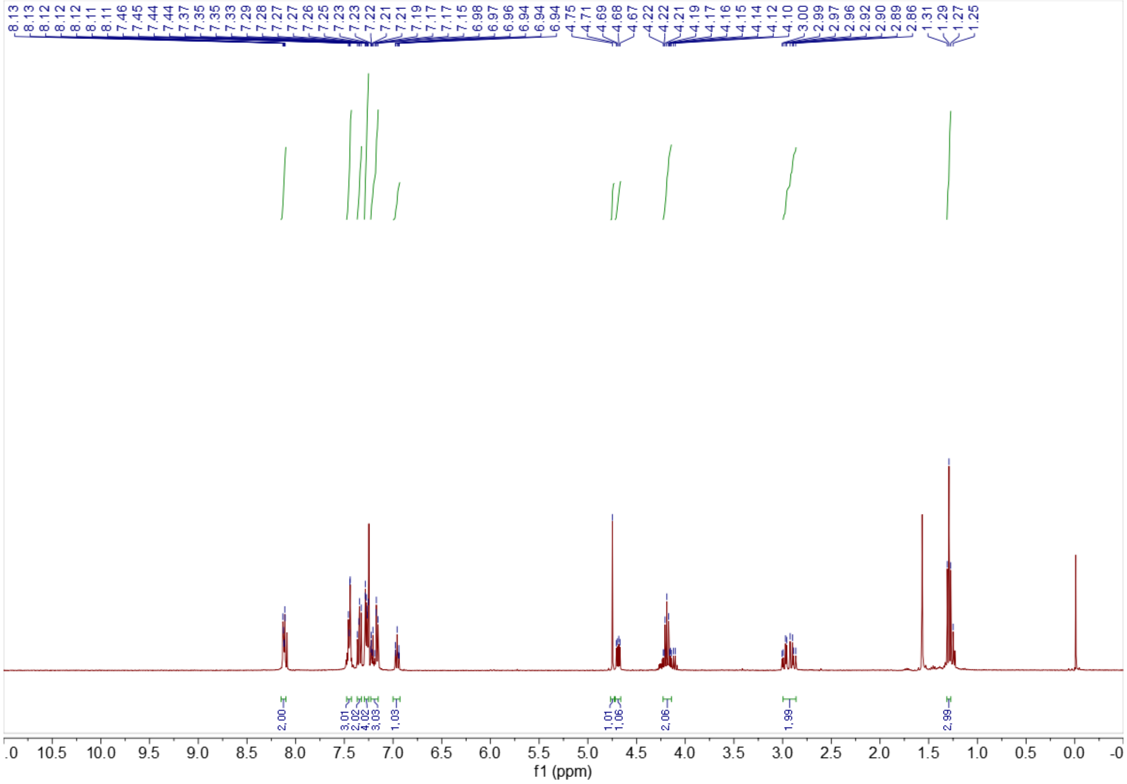

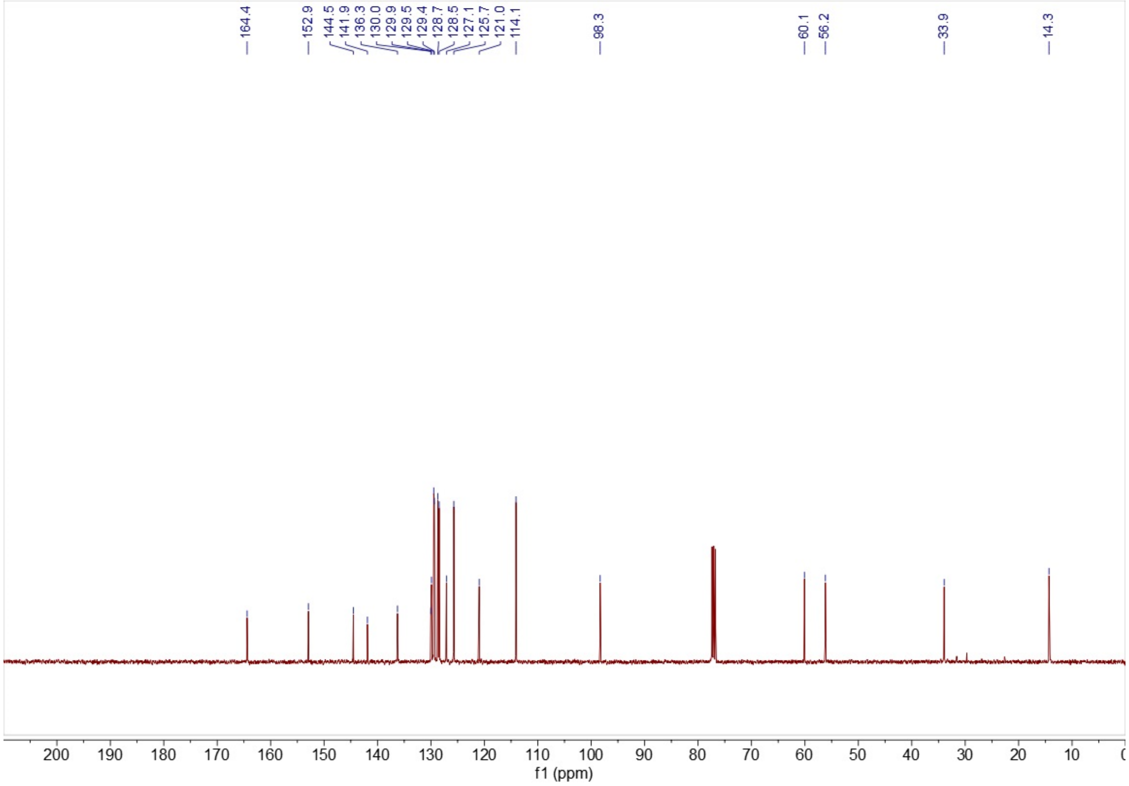

(*Z*)-Ethyl 2-(5-benzyl-4-(4-methoxyphenyl)-2-phenyl-4*H*-1,3,4-oxadiazin-6(5*H*)-ylidene)acetate (3b)

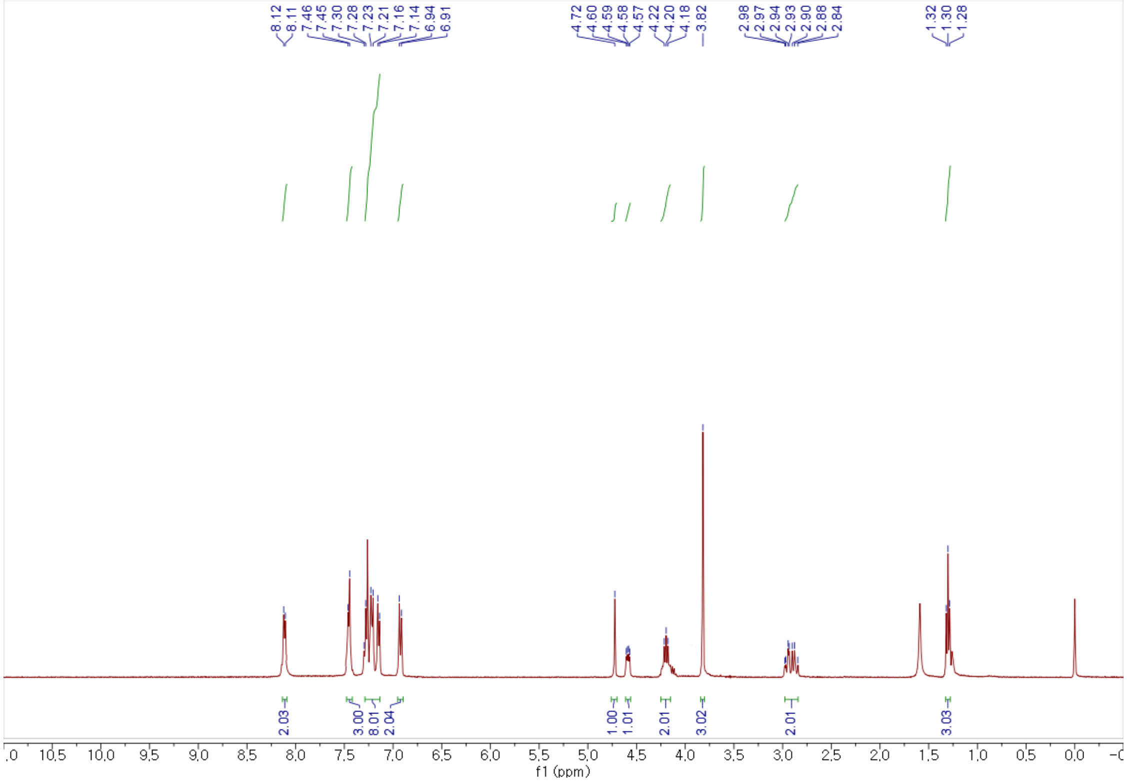

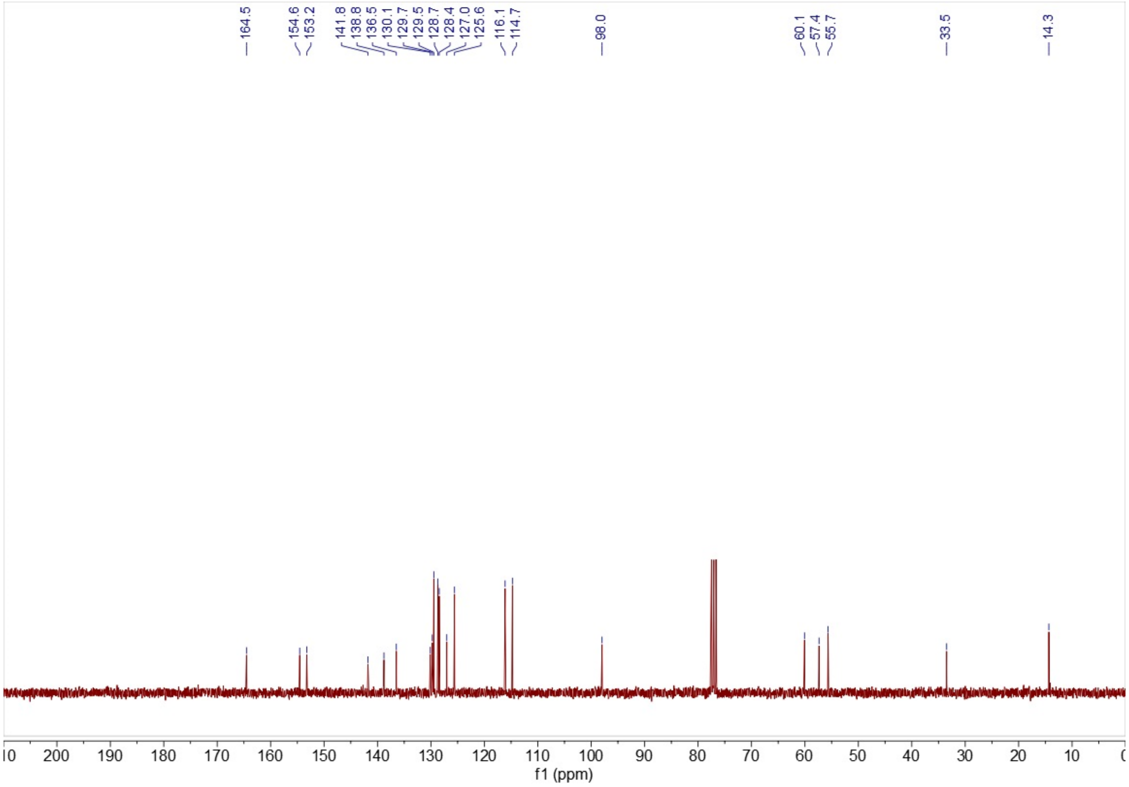

(*Z*)-Ethyl 2-(5-benzyl-2-phenyl-4-(*p*-tolyl)-4*H*-1,3,4-oxadiazin-6(5*H*)-ylidene)acetate (3c)

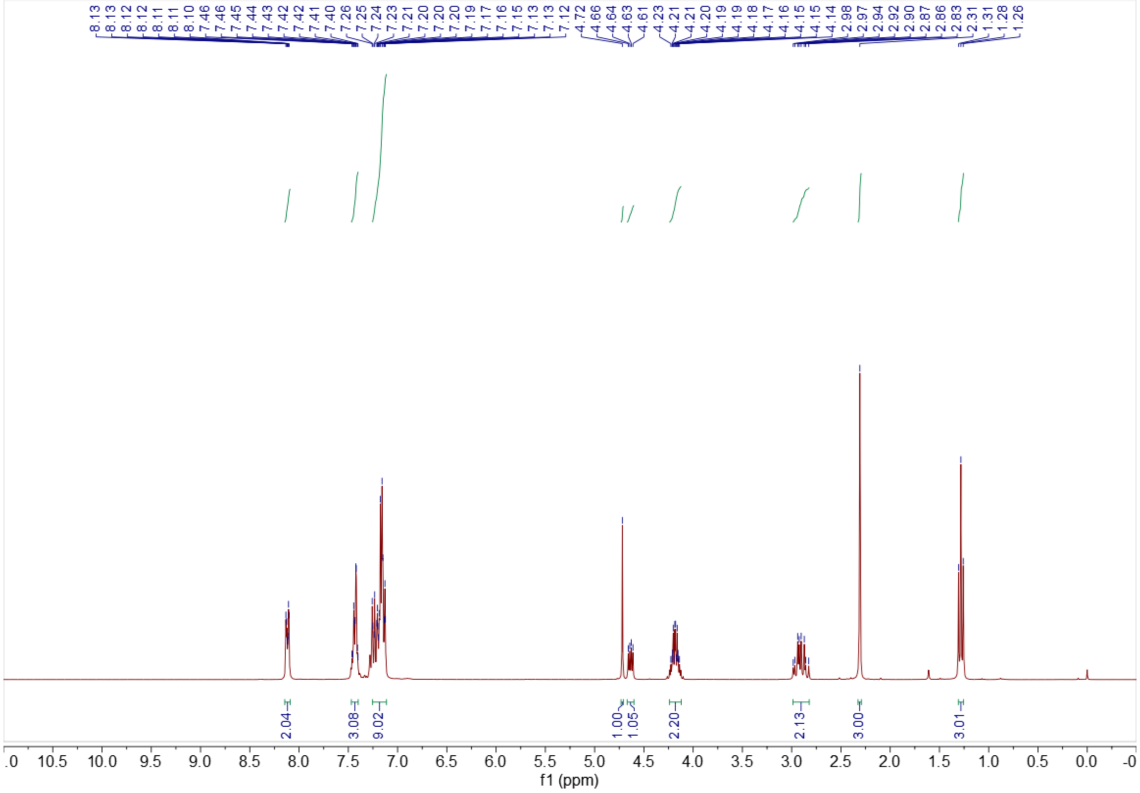

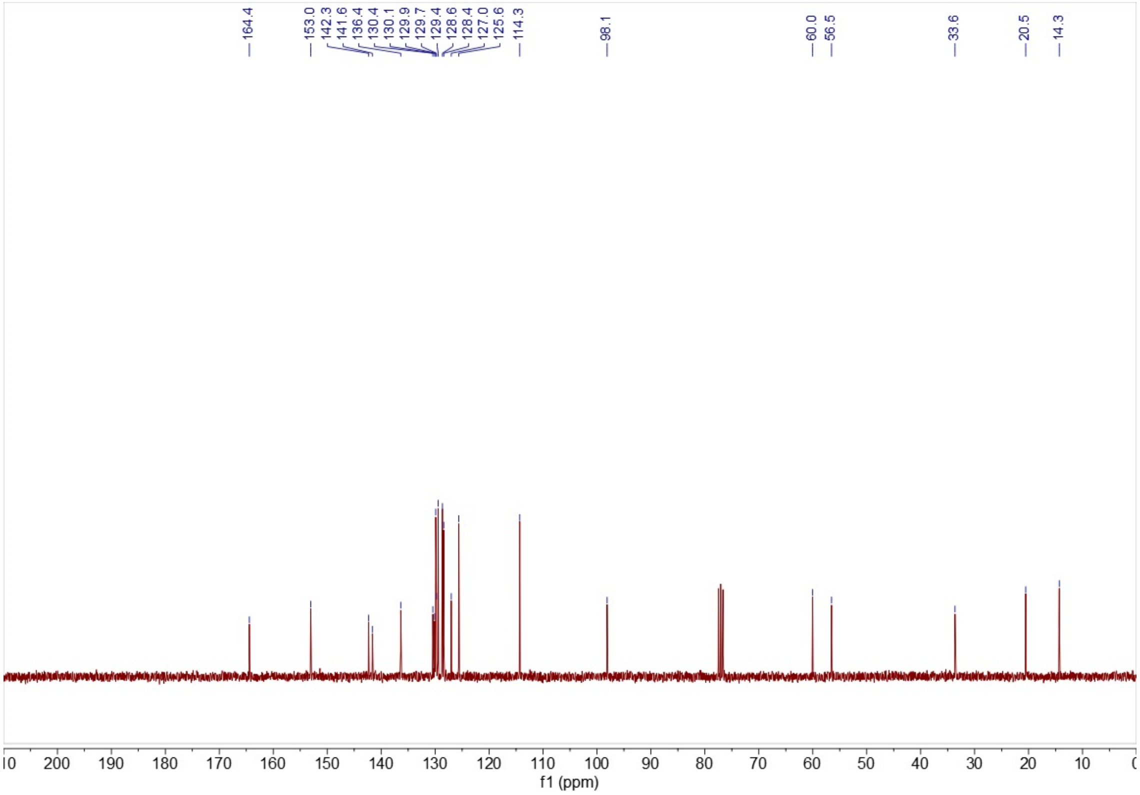

(*Z*)-Ethyl 2-(5-benzyl-4-(4-chlorophenyl)-2-phenyl-4*H*-1,3,4-oxadiazin-6(5*H*)-ylidene)acetate (3d)

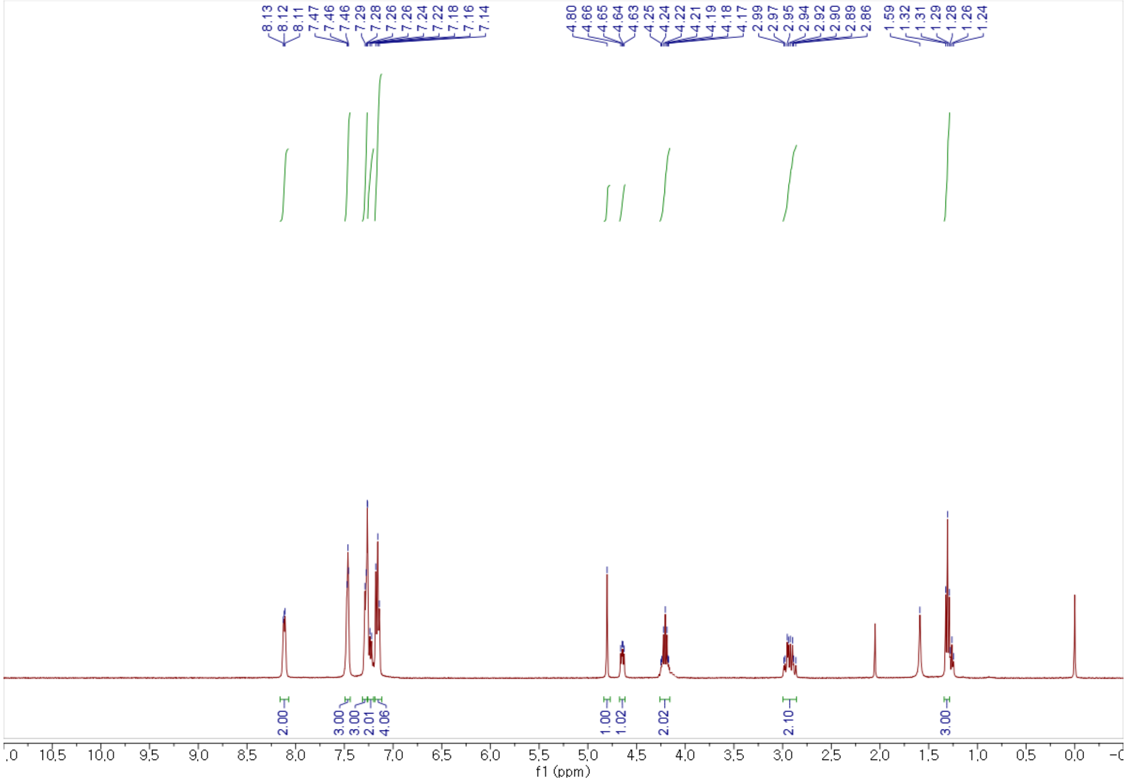

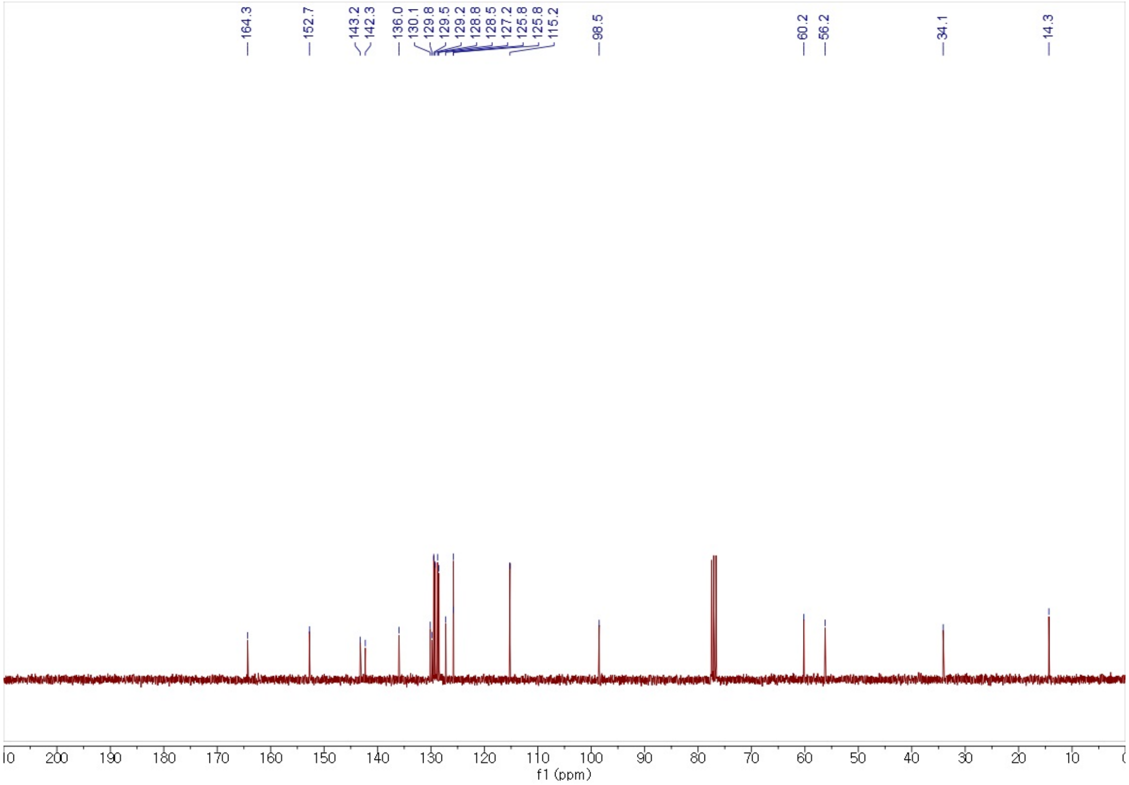

(*Z*)-Ethyl 2-(5-benzyl-4-(4-bromophenyl)-2-phenyl-4*H*-1,3,4-oxadiazin-6(5*H*)-ylidene)acetate (3e)

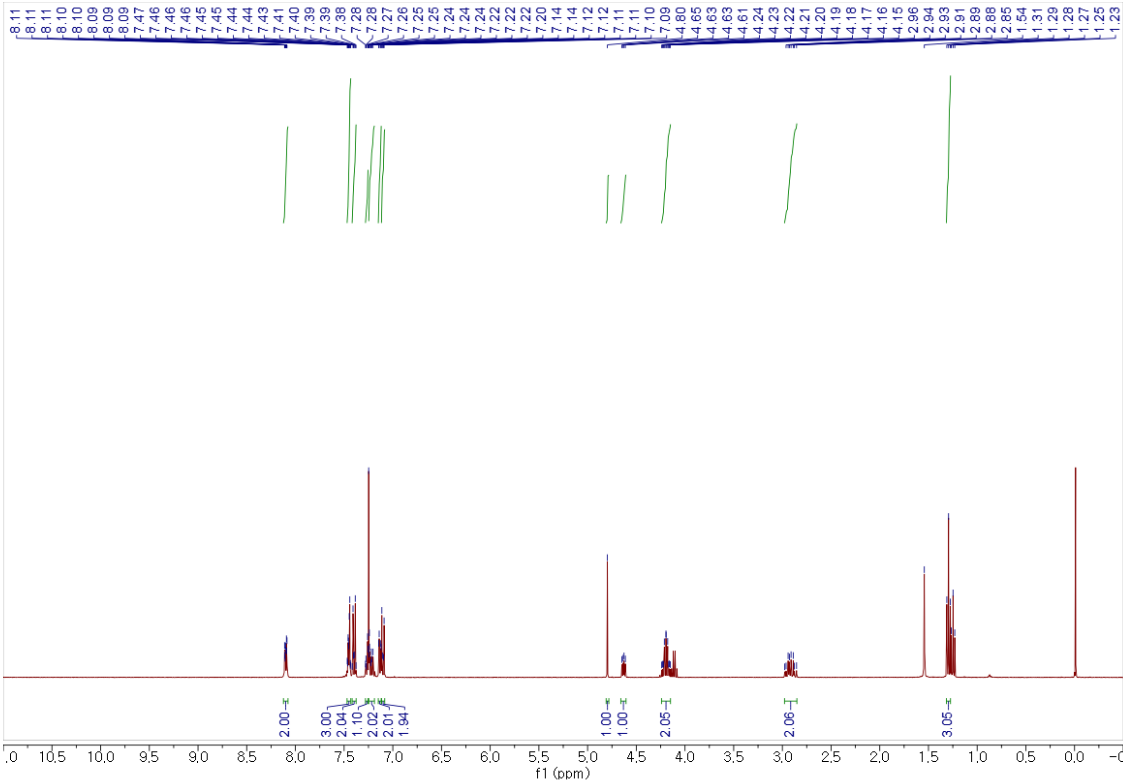

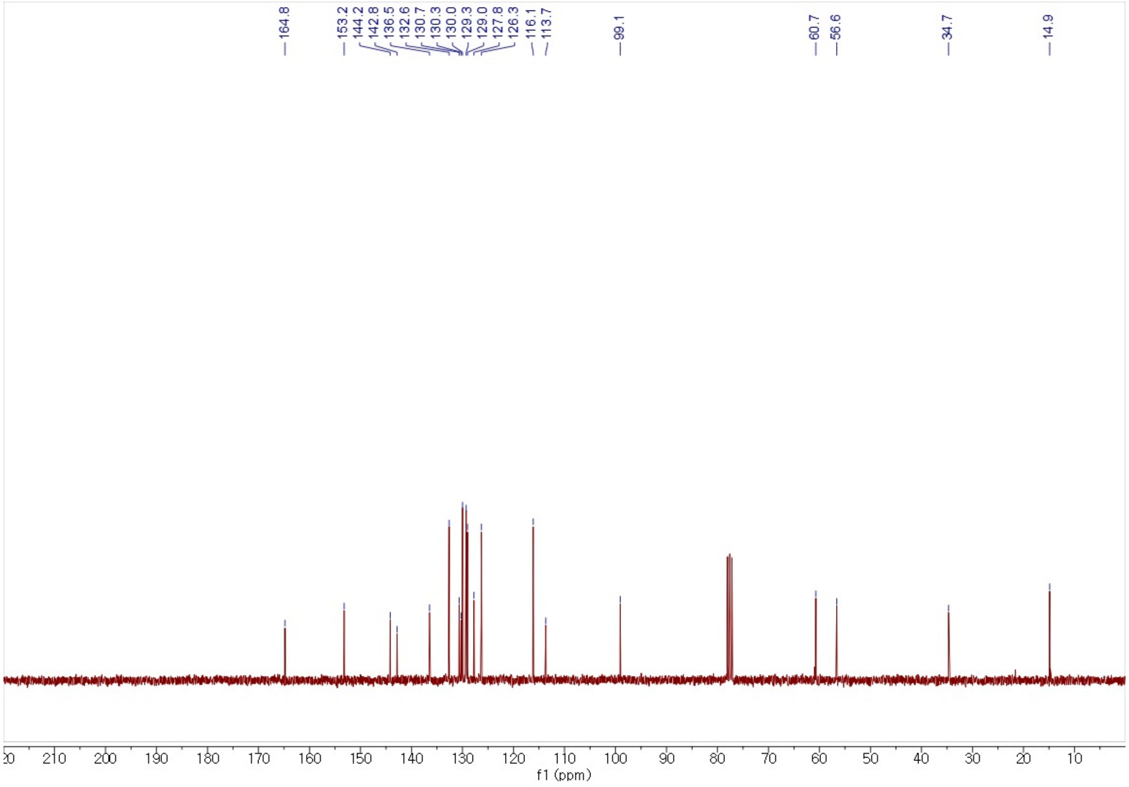

(*Z*)-ethyl 2-(5-benzyl-4-(4-nitrophenyl)-2-phenyl-4*H*-1,3,4-oxadiazin-6(5*H*)-ylidene)acetate (3f)

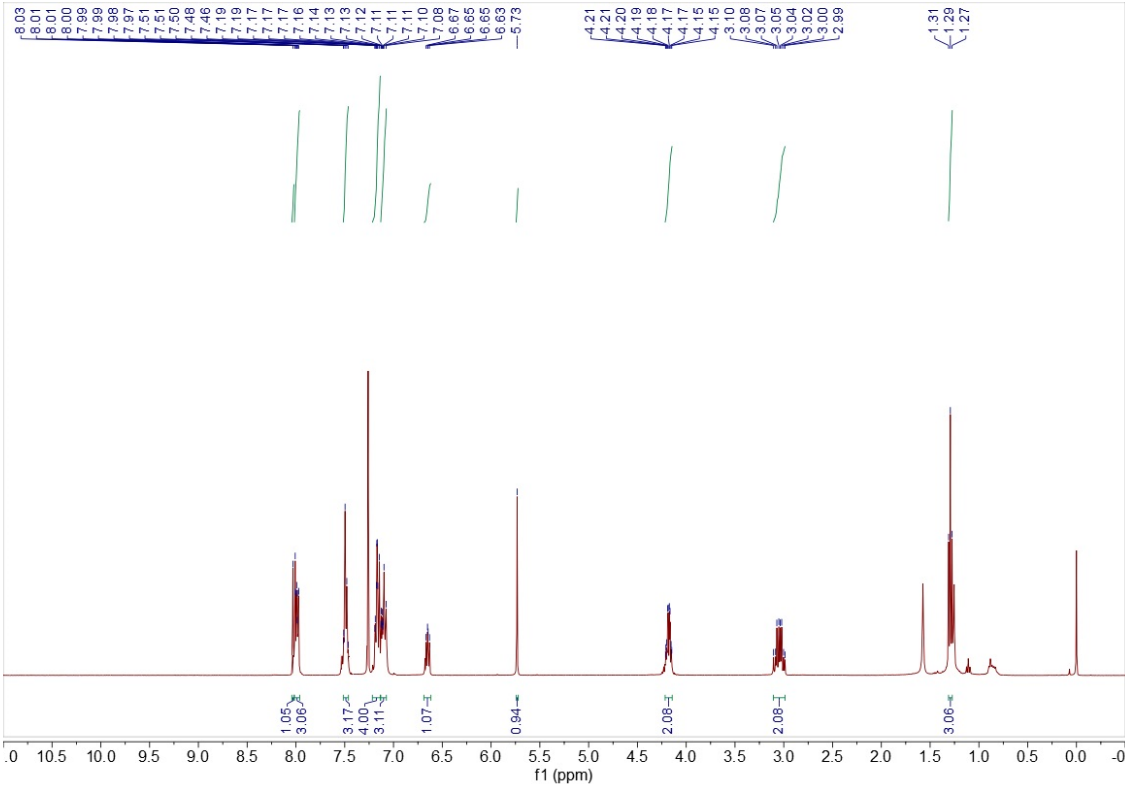

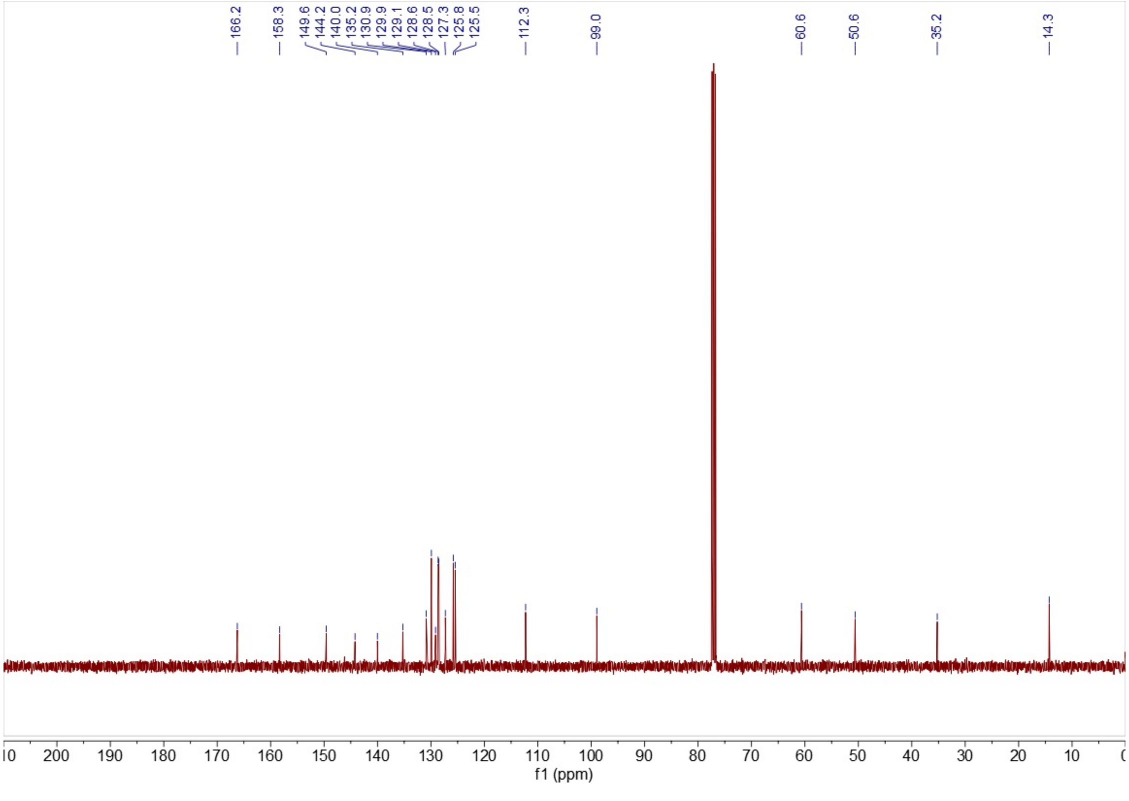

(*Z*)-Ethyl 2-(5-benzyl-2-phenyl-4-(*m*-tolyl)-4*H*-1,3,4-oxadiazin-6(5*H*)-ylidene)acetate (3g)

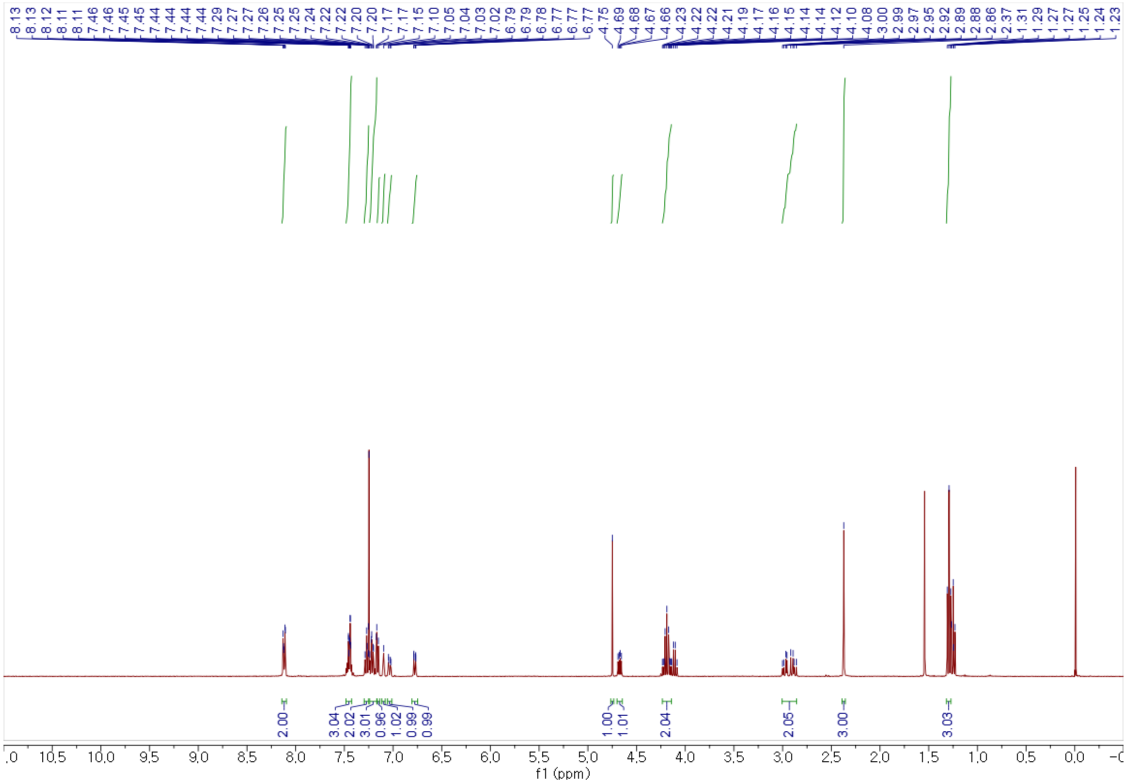

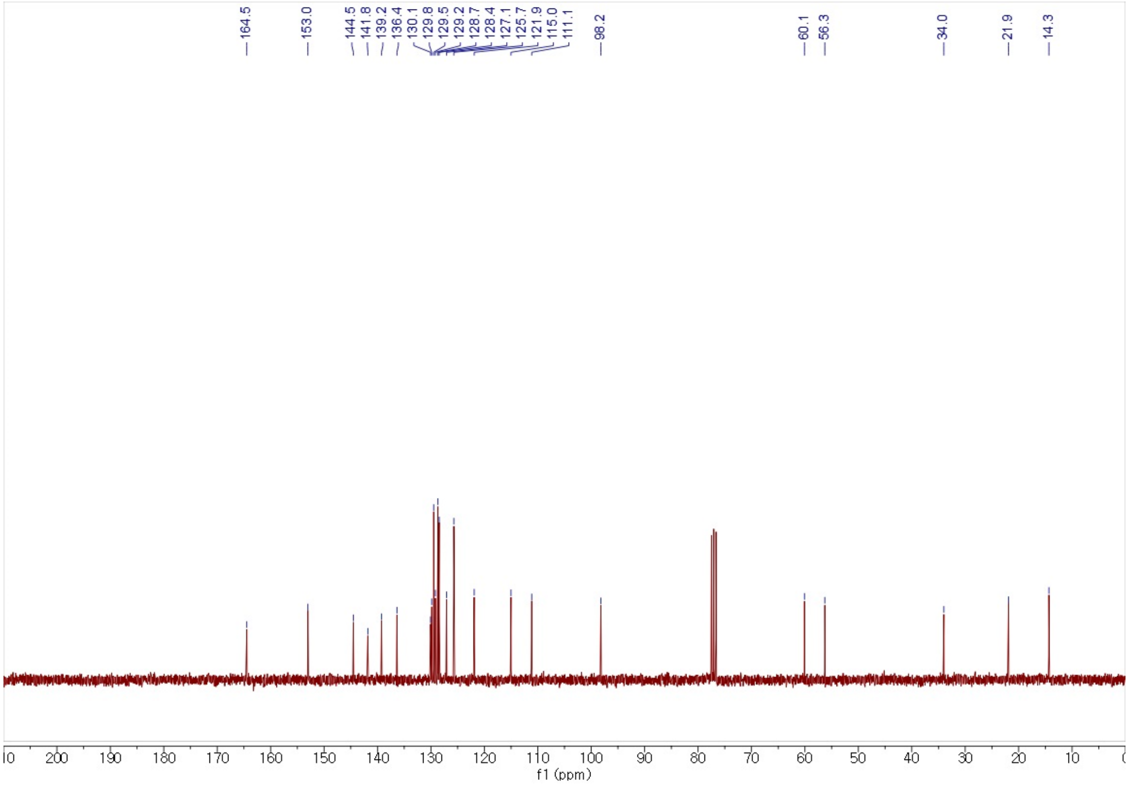

(*Z*)-Ethyl 2-(5-benzyl-4-(3-bromophenyl)-2-phenyl-4*H*-1,3,4-oxadiazin-6(5*H*)-ylidene)acetate (3h)

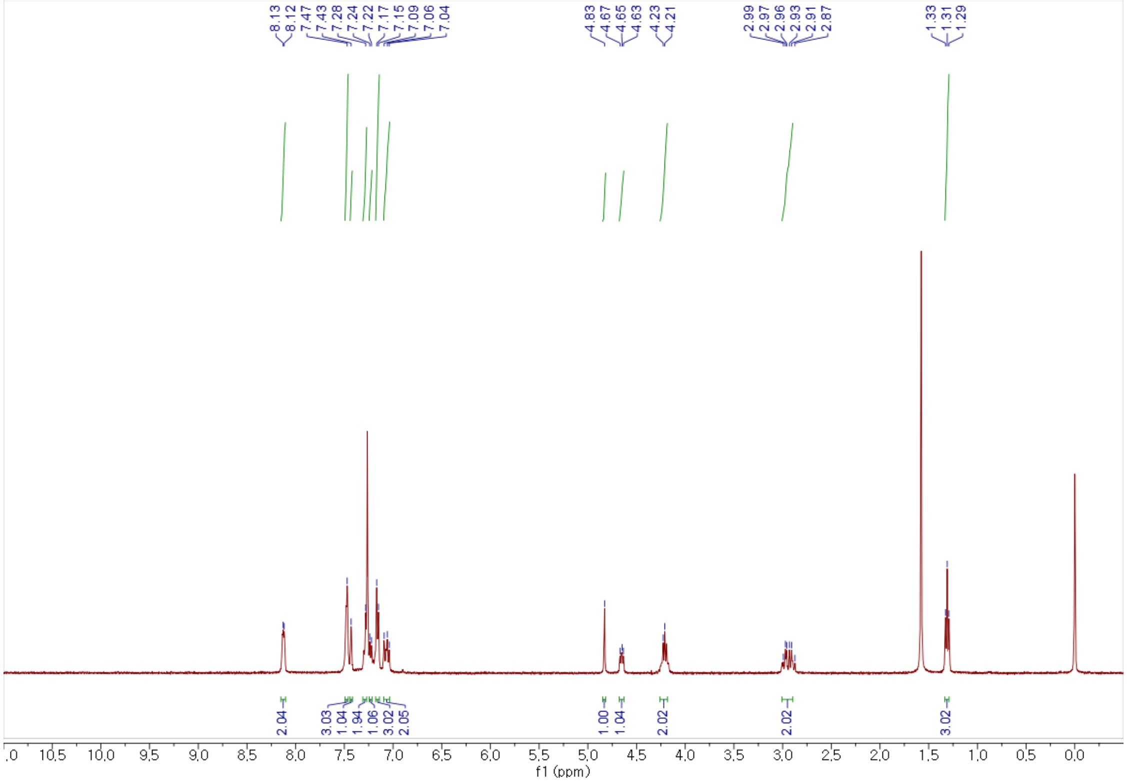

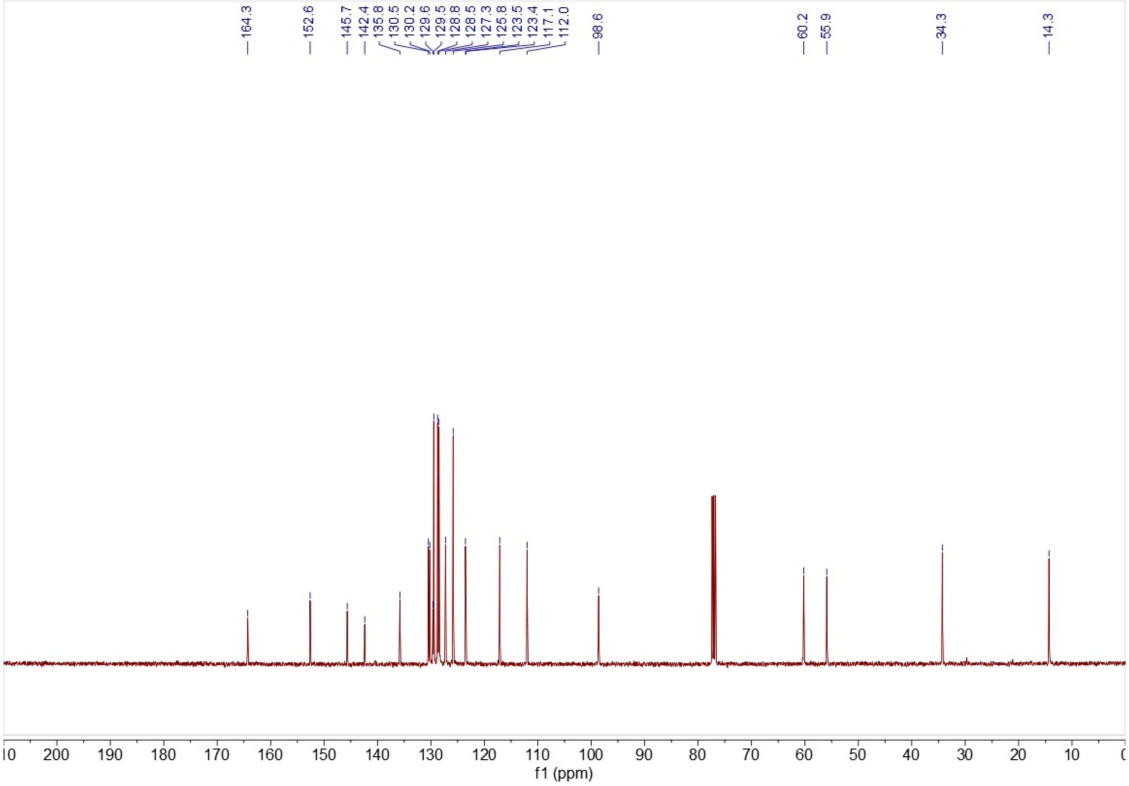

(*Z*)-Ethyl 2-(5-benzyl-4-(3,4-dichlorophenyl)-2-phenyl-4*H*-1,3,4-oxadiazin-6(5*H*)-ylidene)acetate (3i)

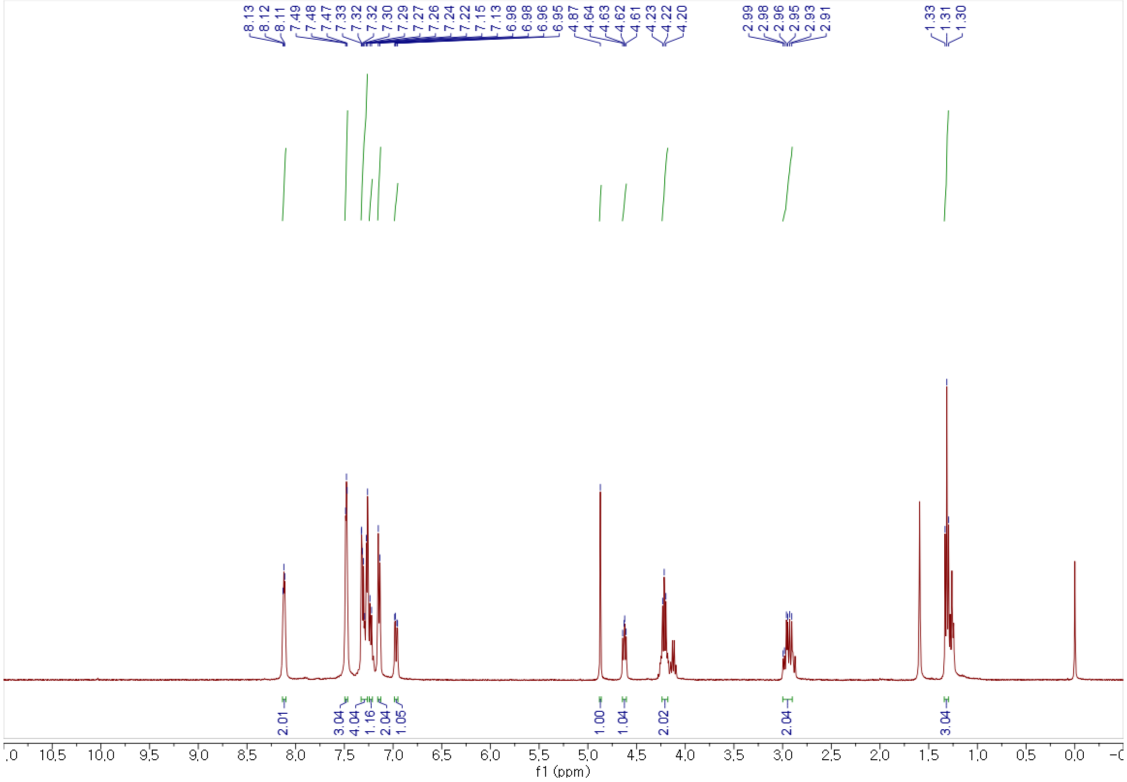

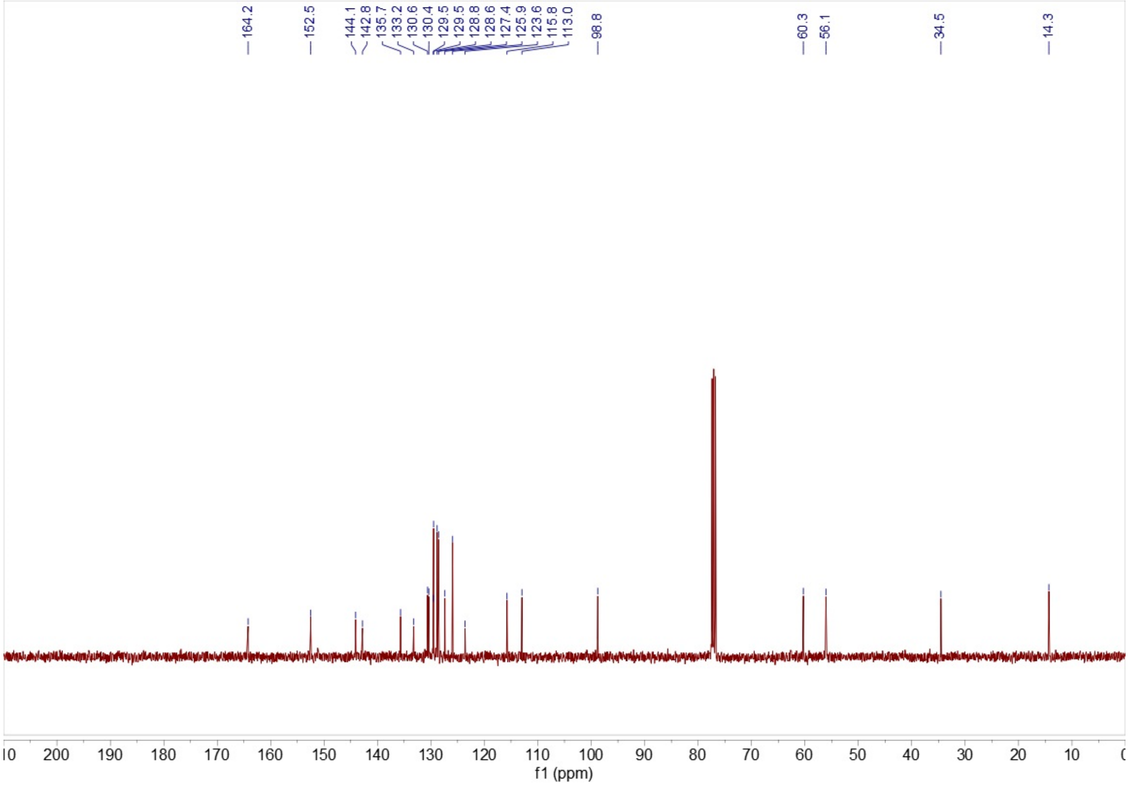

(*Z*)-Ethyl 2-(5-benzyl-4-(3,5-dichlorophenyl)-2-phenyl-4*H*-1,3,4-oxadiazin-6(5*H*)-ylidene)acetate (3j)

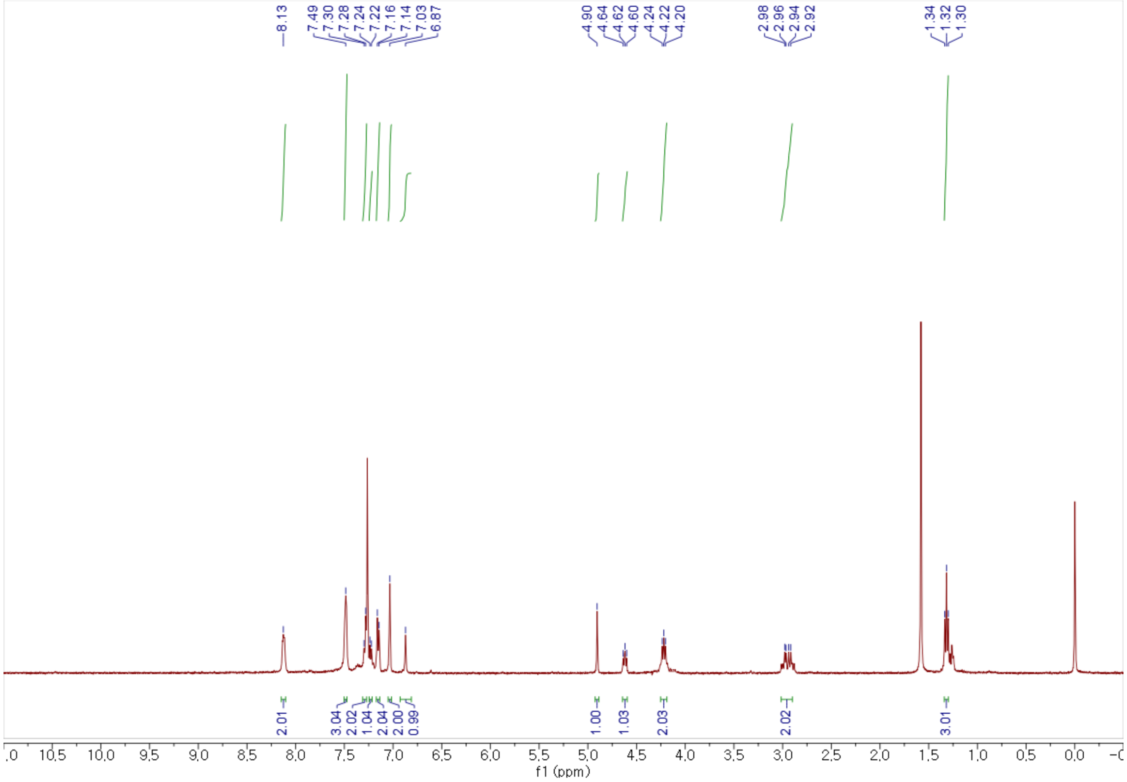

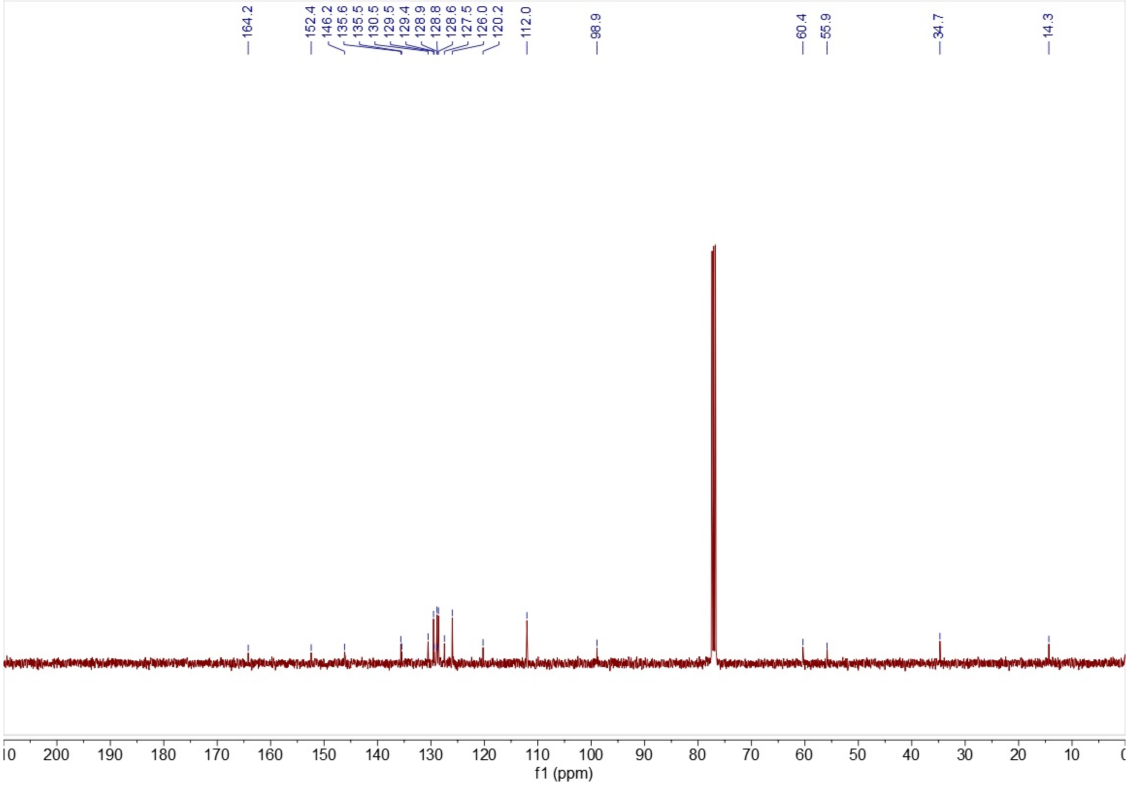

(*Z*)-Ethyl 2-(5-benzyl-2-(4-methoxyphenyl)-4-phenyl-4*H*-1,3,4-oxadiazin-6(5*H*)-ylidene)acetate (3m)

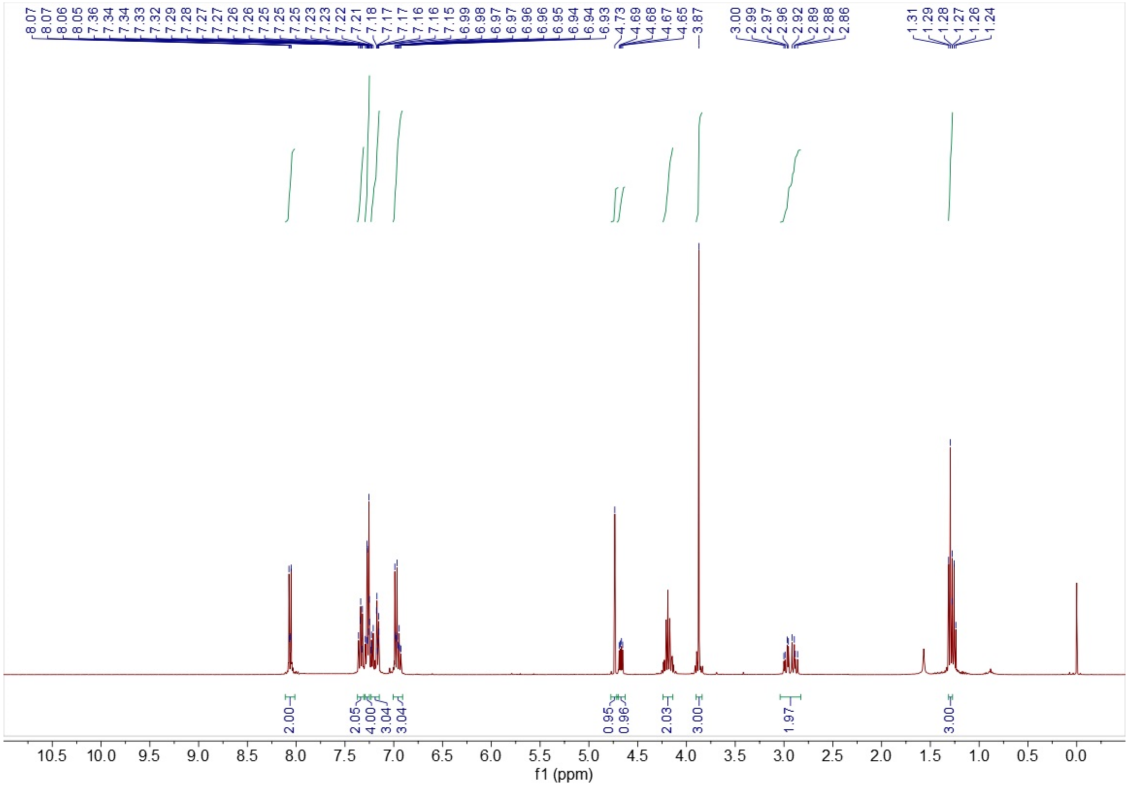

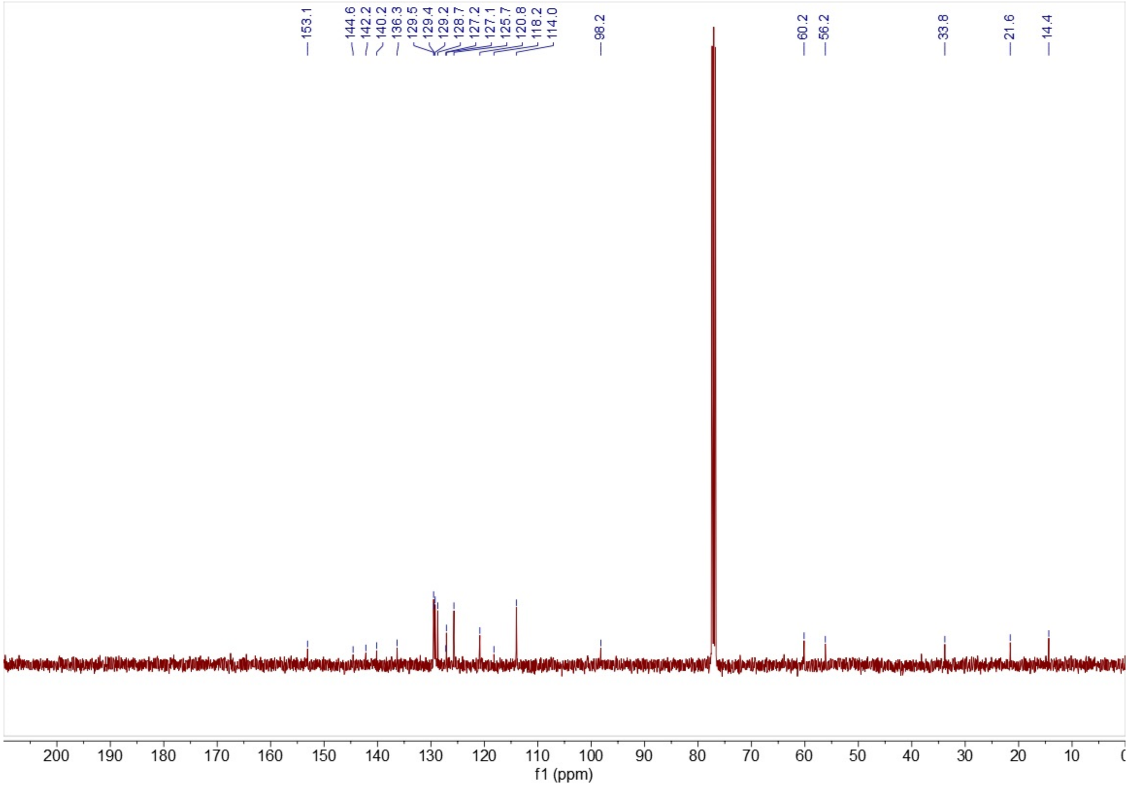

(*Z*)-Ethyl 2-(5-benzyl-4-phenyl-2-(*p*-tolyl)-4*H*-1,3,4-oxadiazin-6(5*H*)-ylidene)acetate (3n)

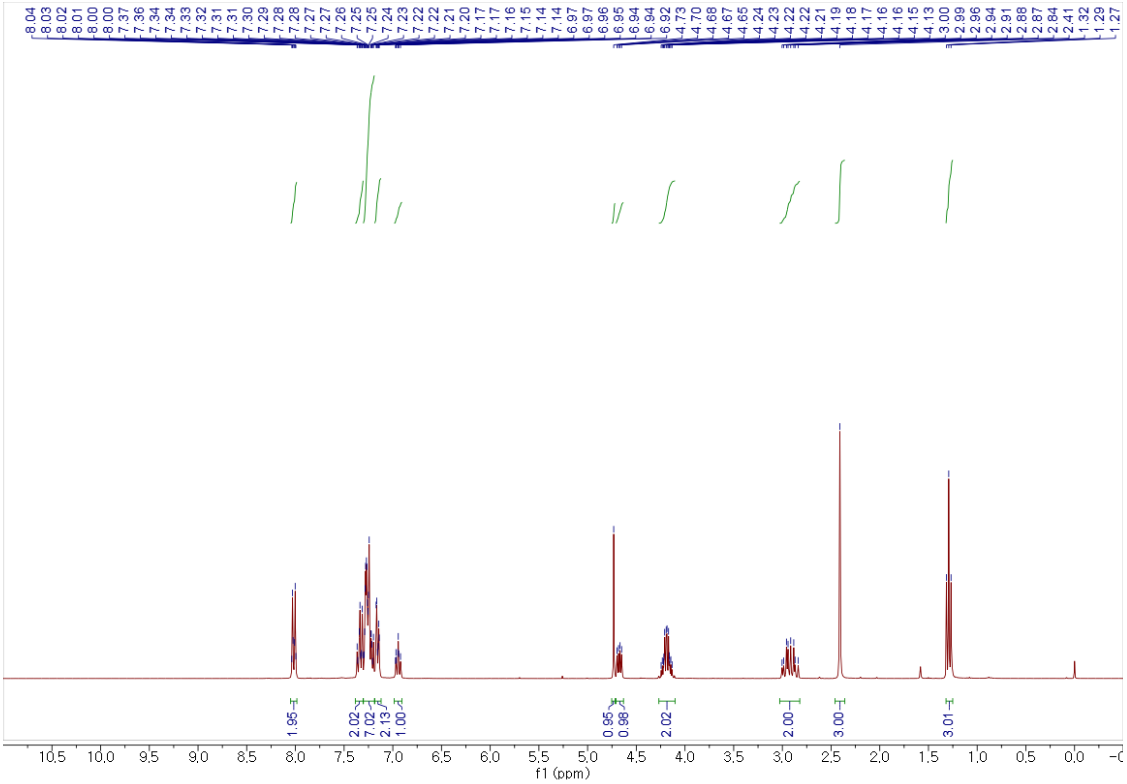

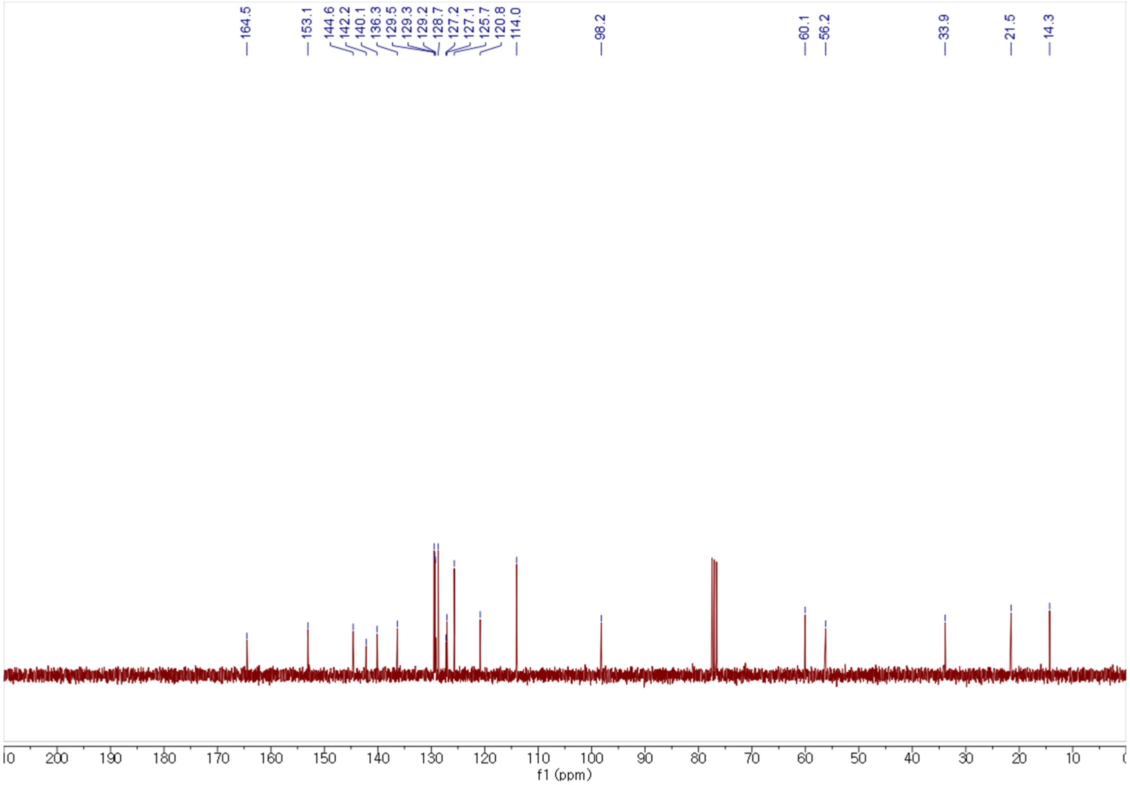

(*Z*)-Ethyl 2-(5-benzyl-2-(4-chlorophenyl)-4-phenyl-4*H*-1,3,4-oxadiazin-6(5*H*)-ylidene)acetate (3o)

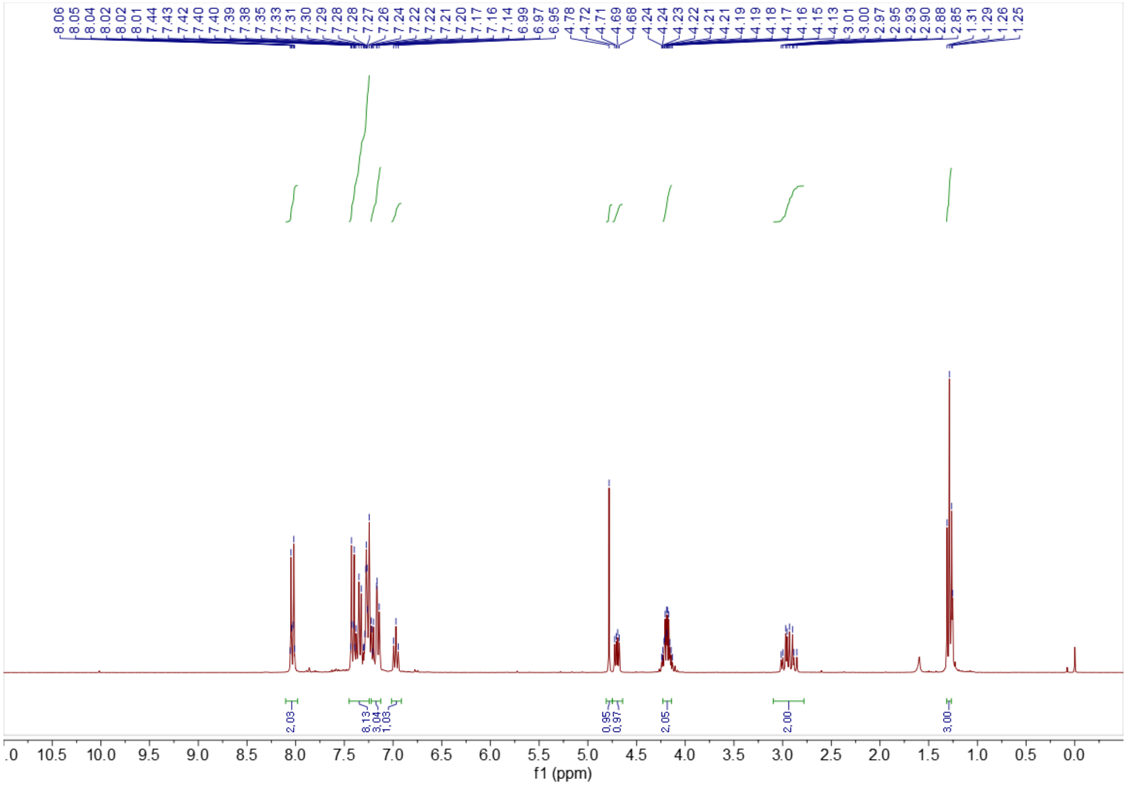

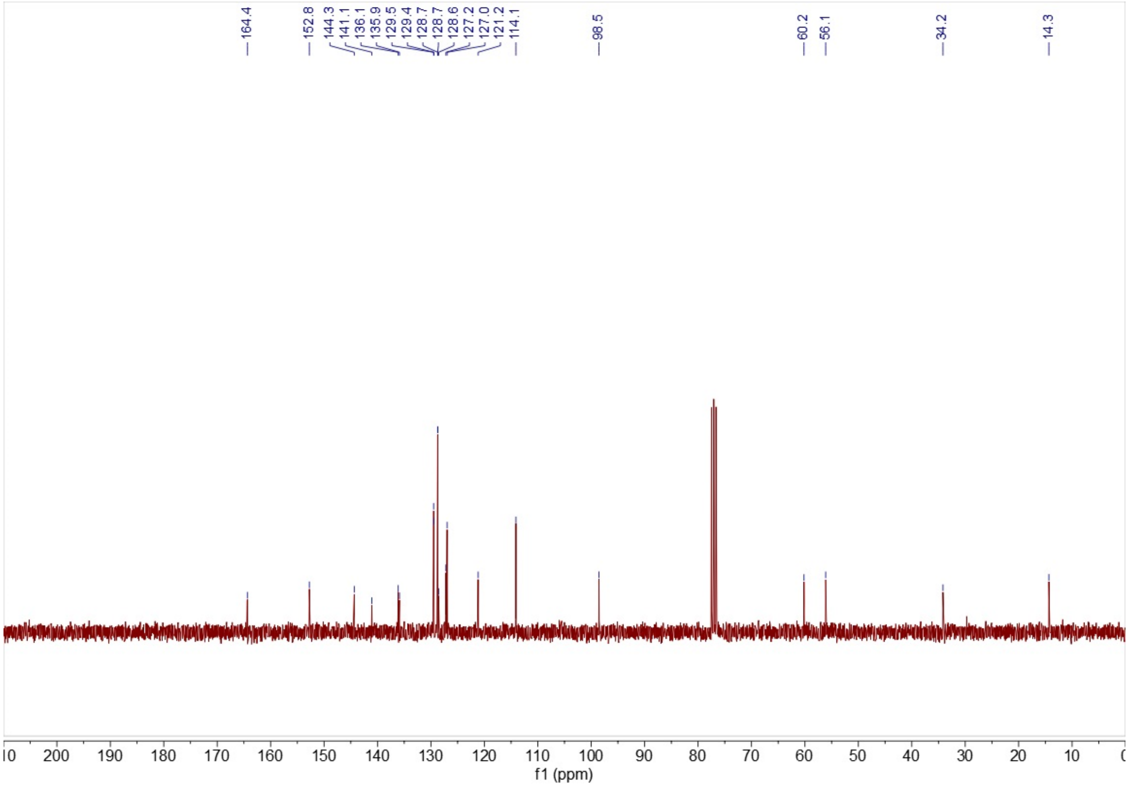

(*Z*)-Ethyl 2-(5-benzyl-2-(4-bromophenyl)-4-phenyl-4*H*-1,3,4-oxadiazin-6(5*H*)-ylidene)acetate (3p)

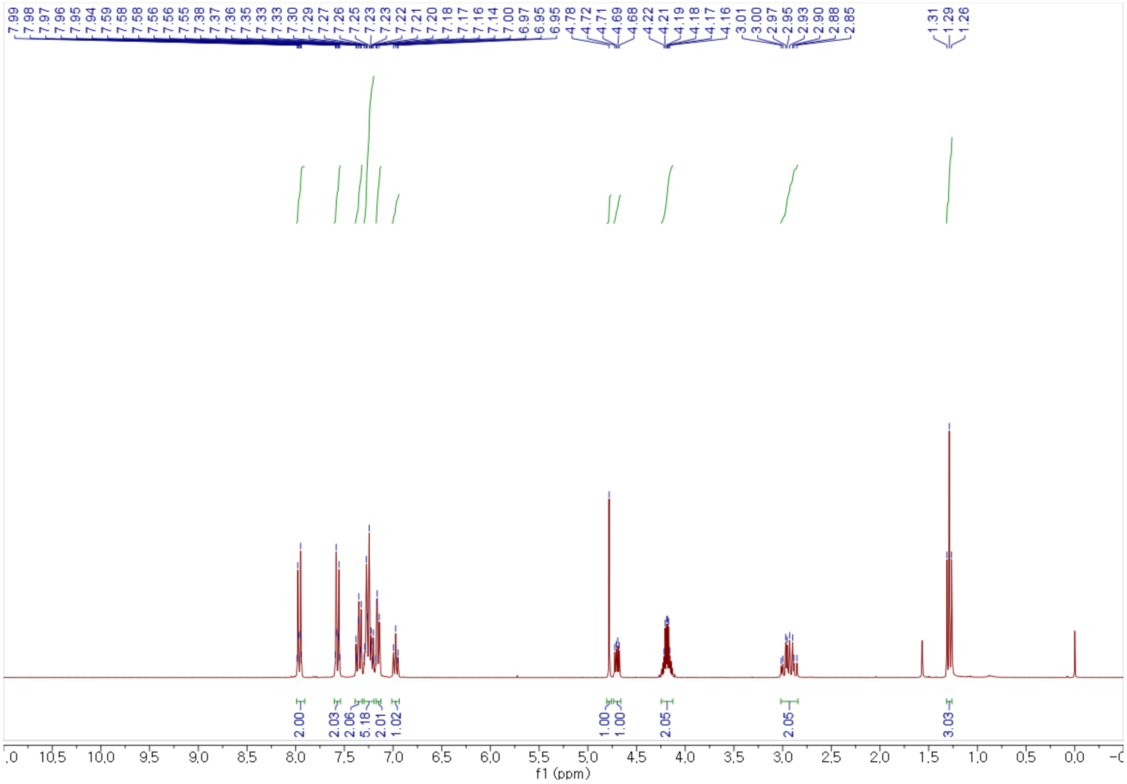

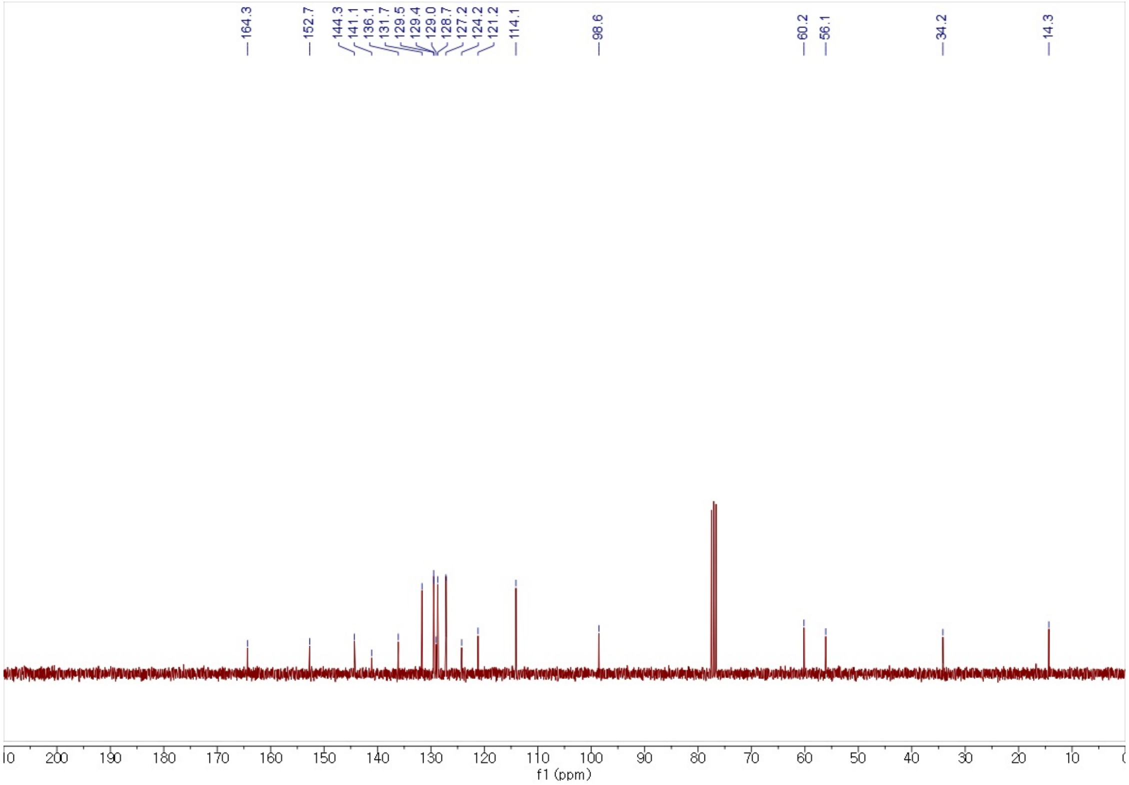

(*Z*)-Ethyl 2-(5-benzyl-2-(4-nitrophenyl)-4-phenyl-4*H*-1,3,4-oxadiazin-6(5*H*)-ylidene)acetate (3q)

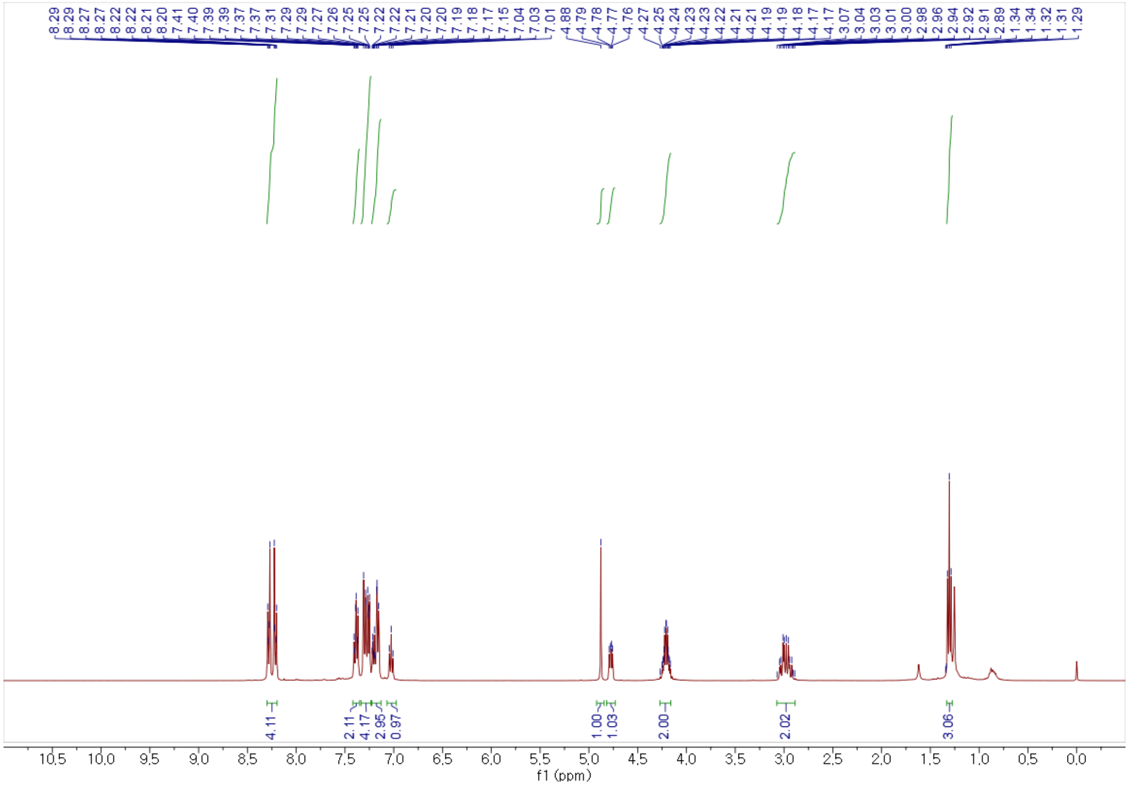

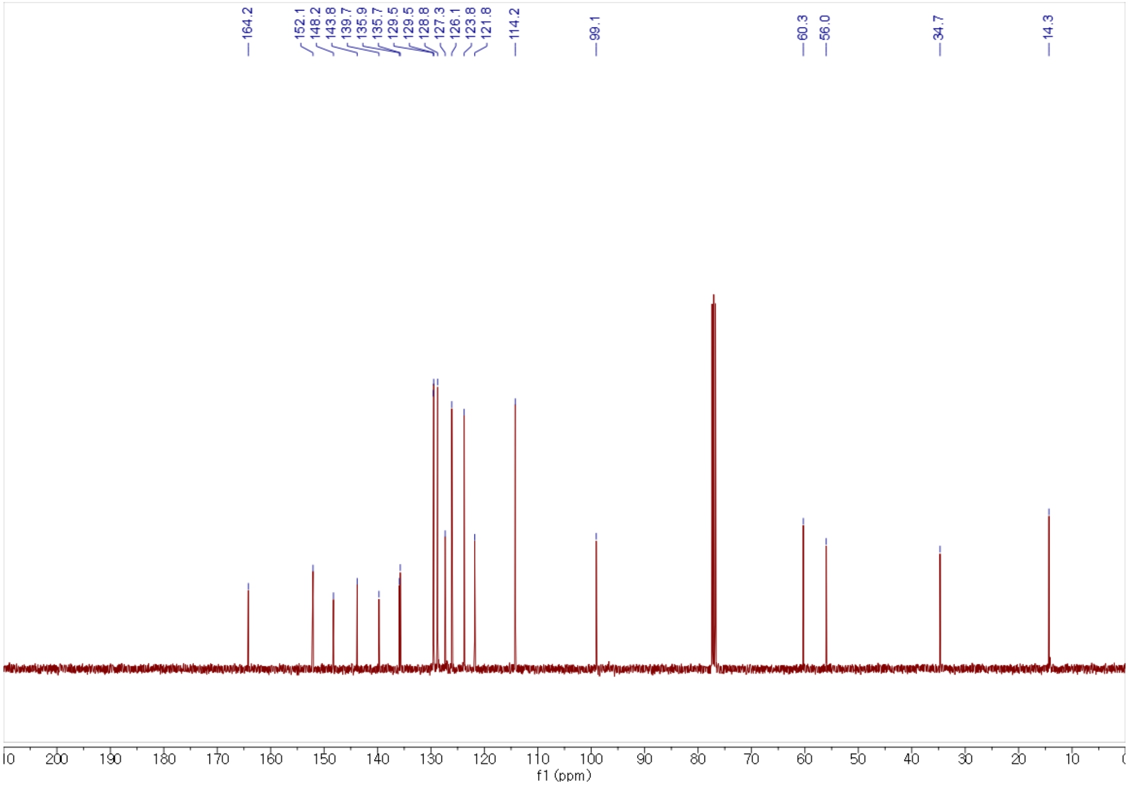

(*Z*)-Ethyl 2-(5-benzyl-4-phenyl-2-(*m*-tolyl)-4*H*-1,3,4-oxadiazin-6(5*H*)-ylidene)acetate (3r)

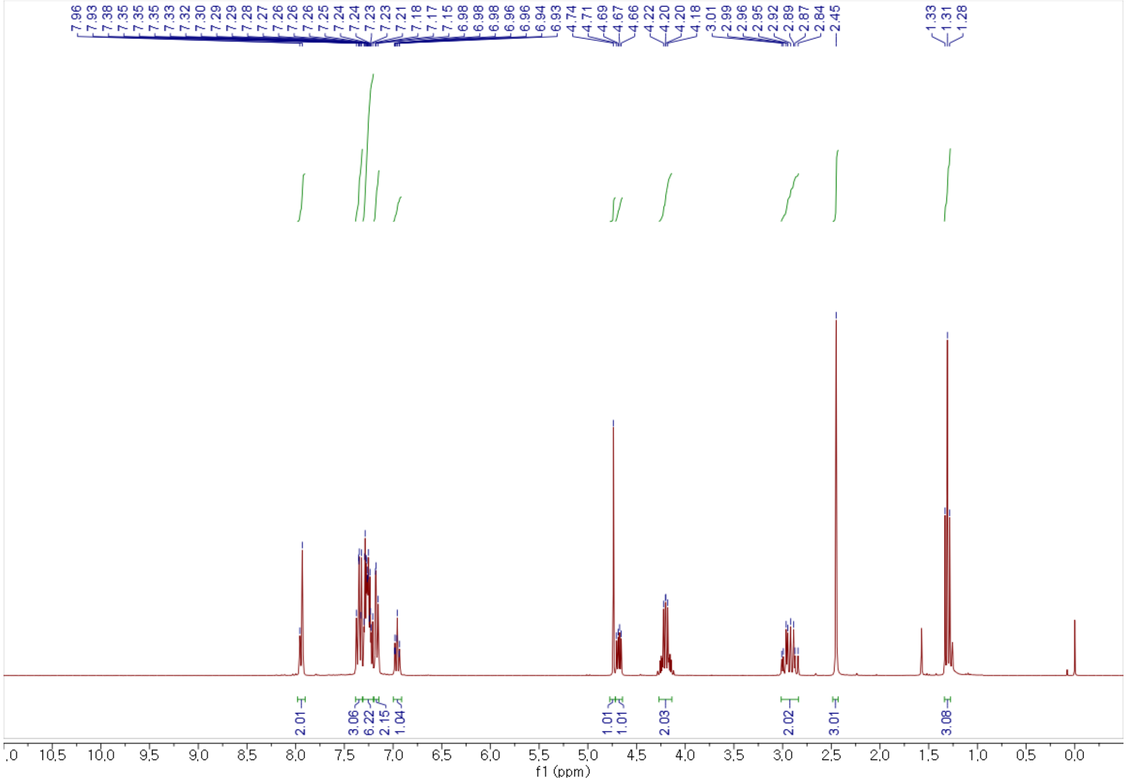

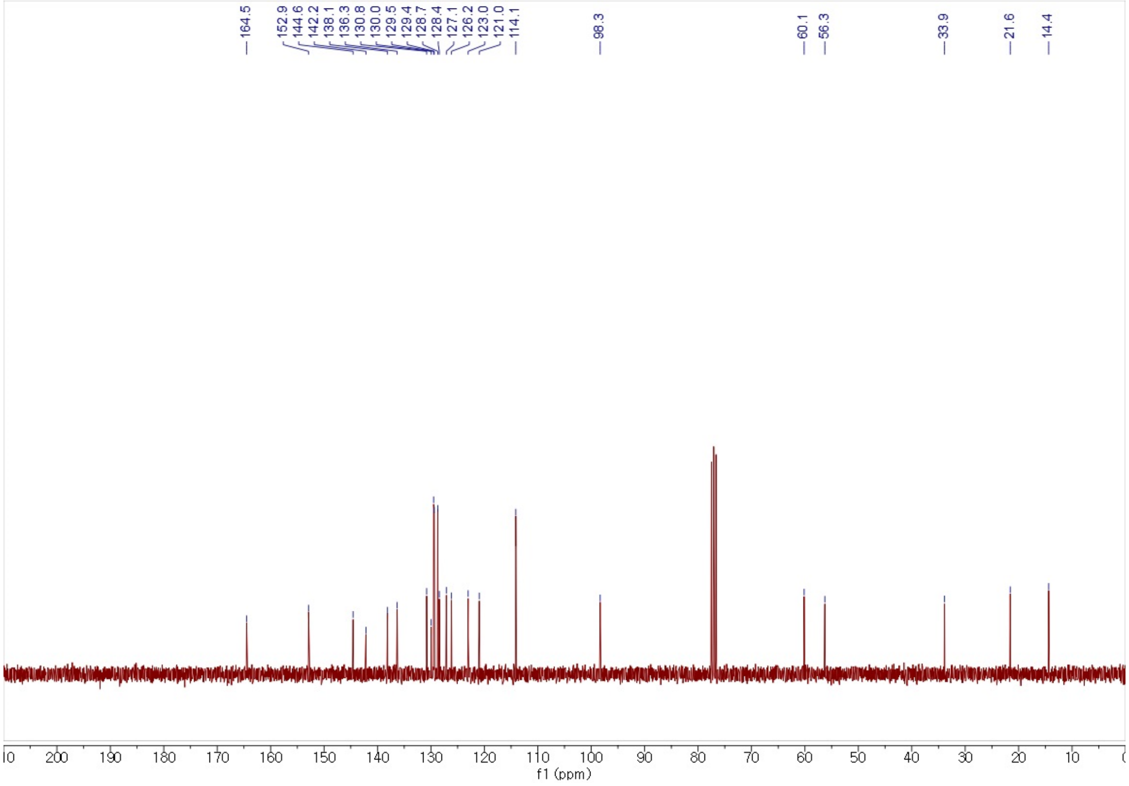

(*Z*)-Ethyl 2-(5-benzyl-2-(3-chlorophenyl)-4-phenyl-4*H*-1,3,4-oxadiazin-6(5*H*)-ylidene)acetate (3s)

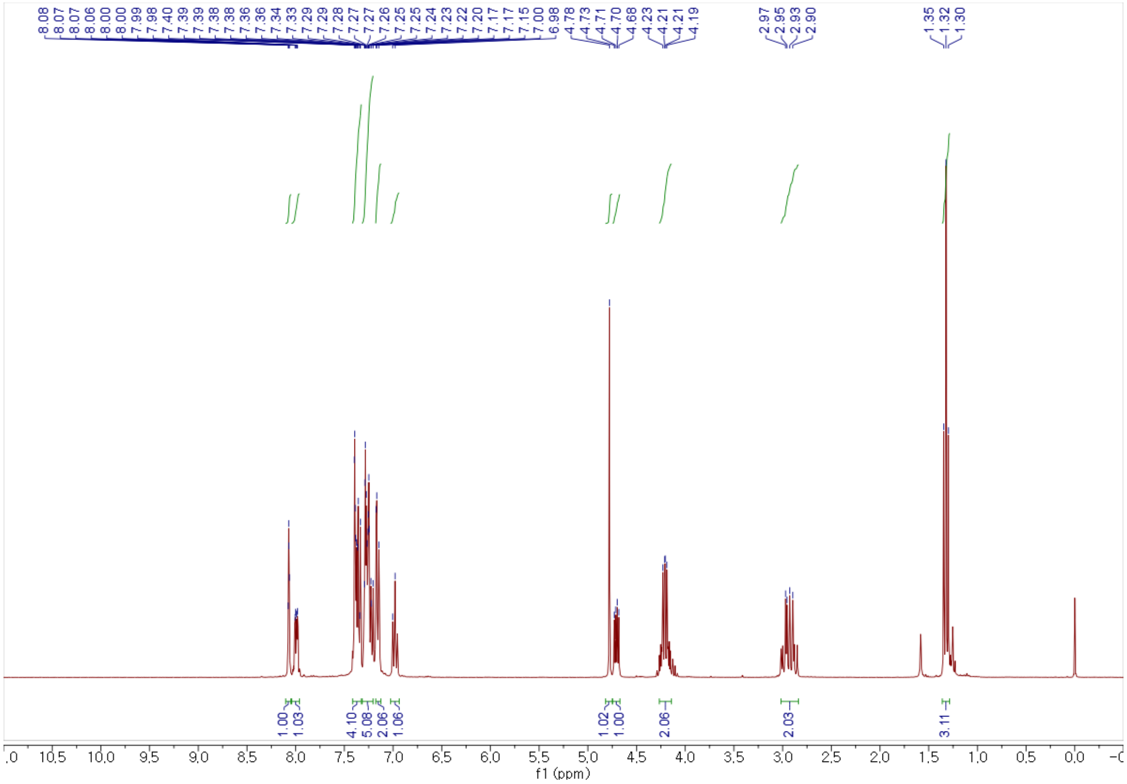

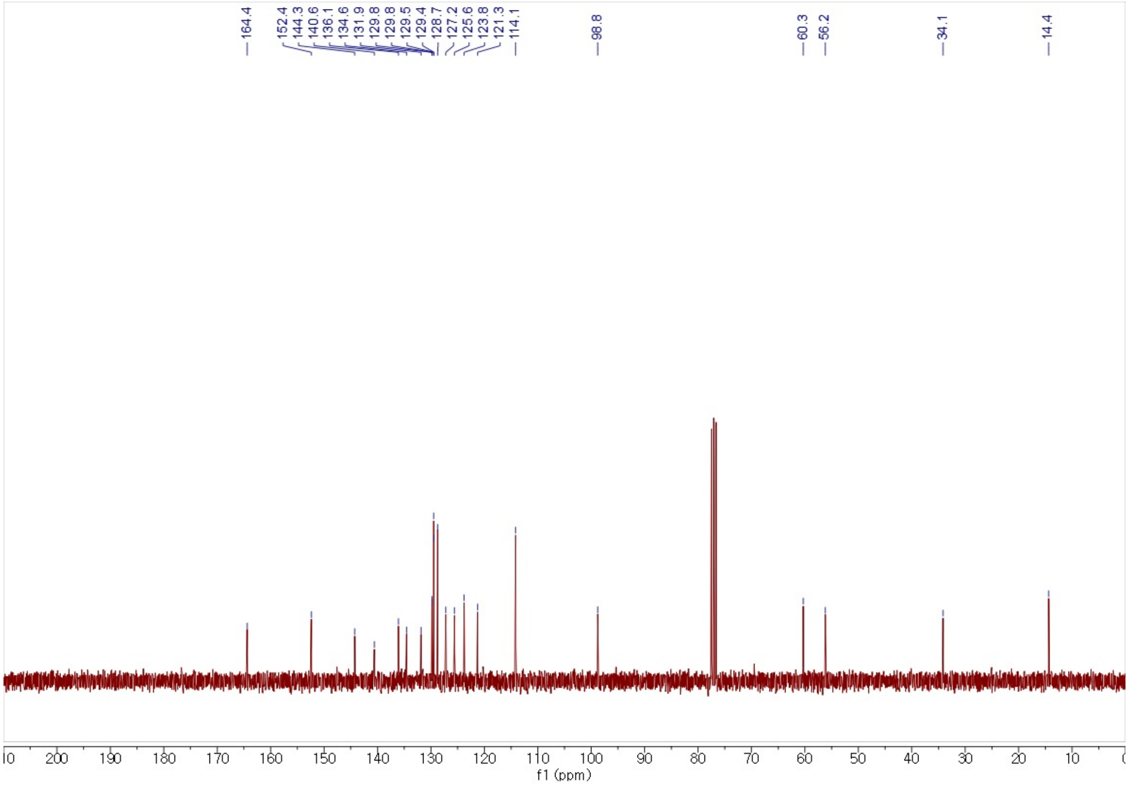

(*Z*)-Ethyl 2-(5-benzyl-2-(naphthalen-2-yl)-4-phenyl-4*H*-1,3,4-oxadiazin-6(5*H*)-ylidene)acetate (3t)

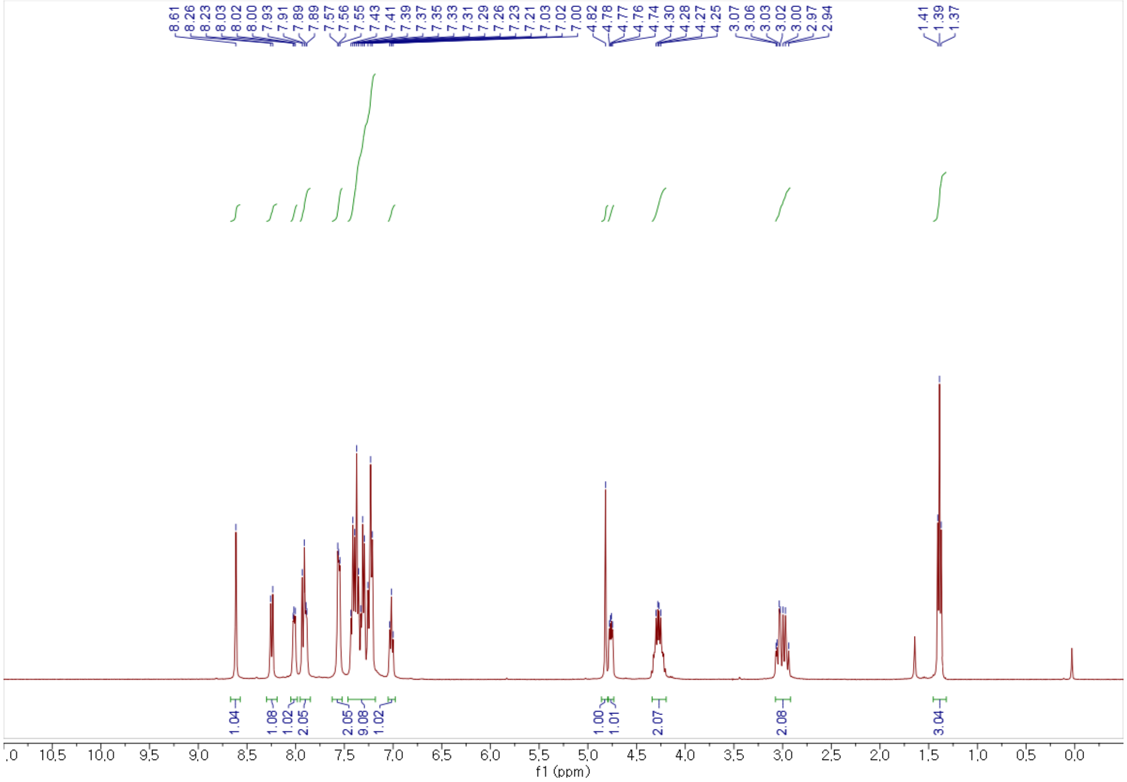

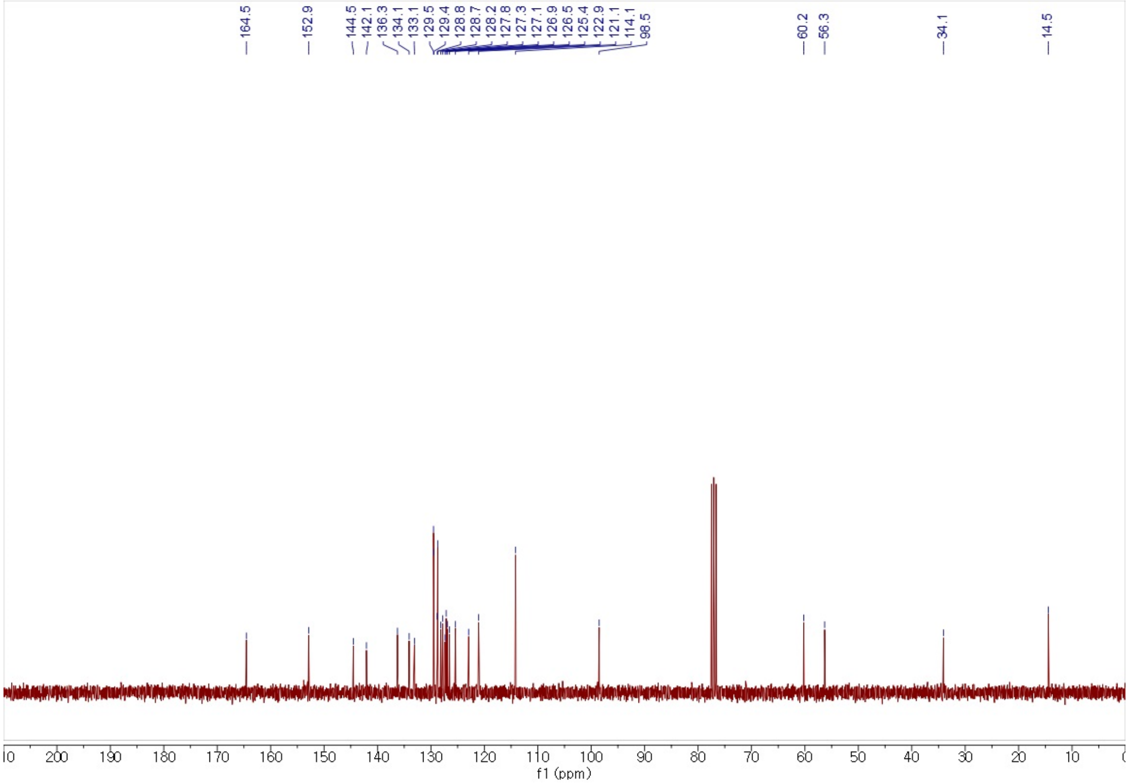

(*Z*)-Ethyl 2-(5-benzyl-2-methyl-4-phenyl-4*H*-1,3,4-oxadiazin-6(5*H*)-ylidene)acetate (3u)

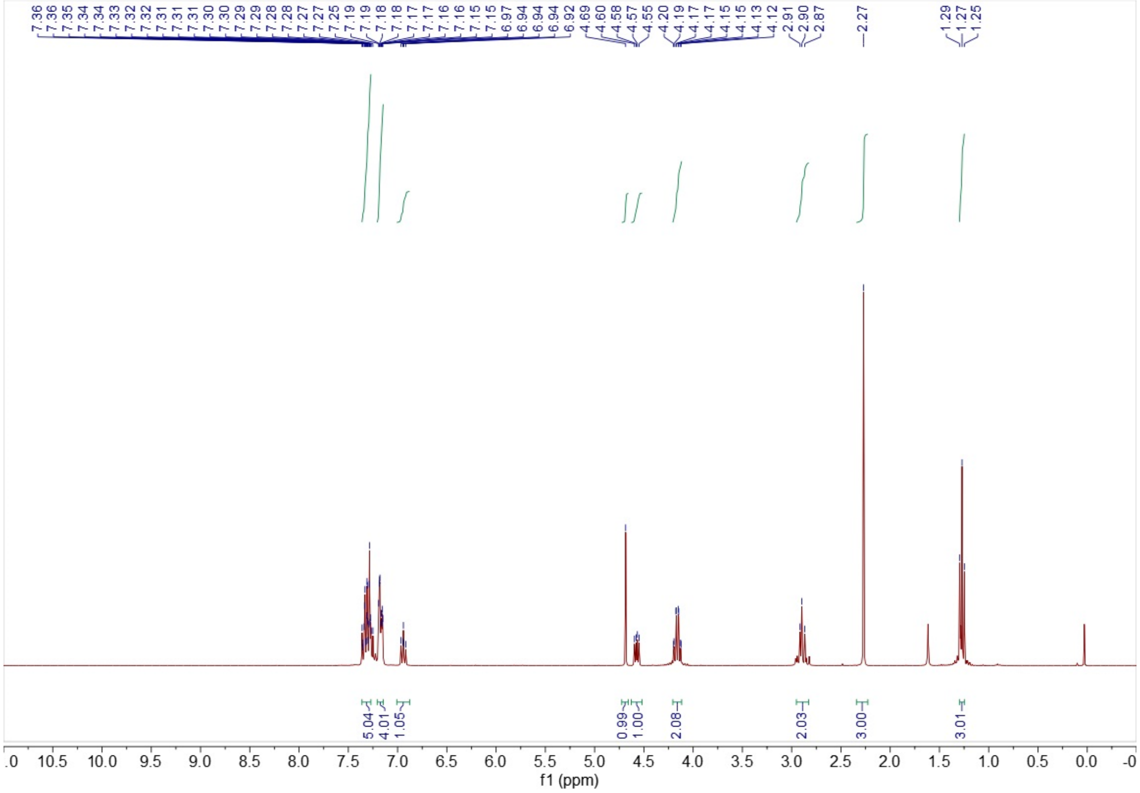

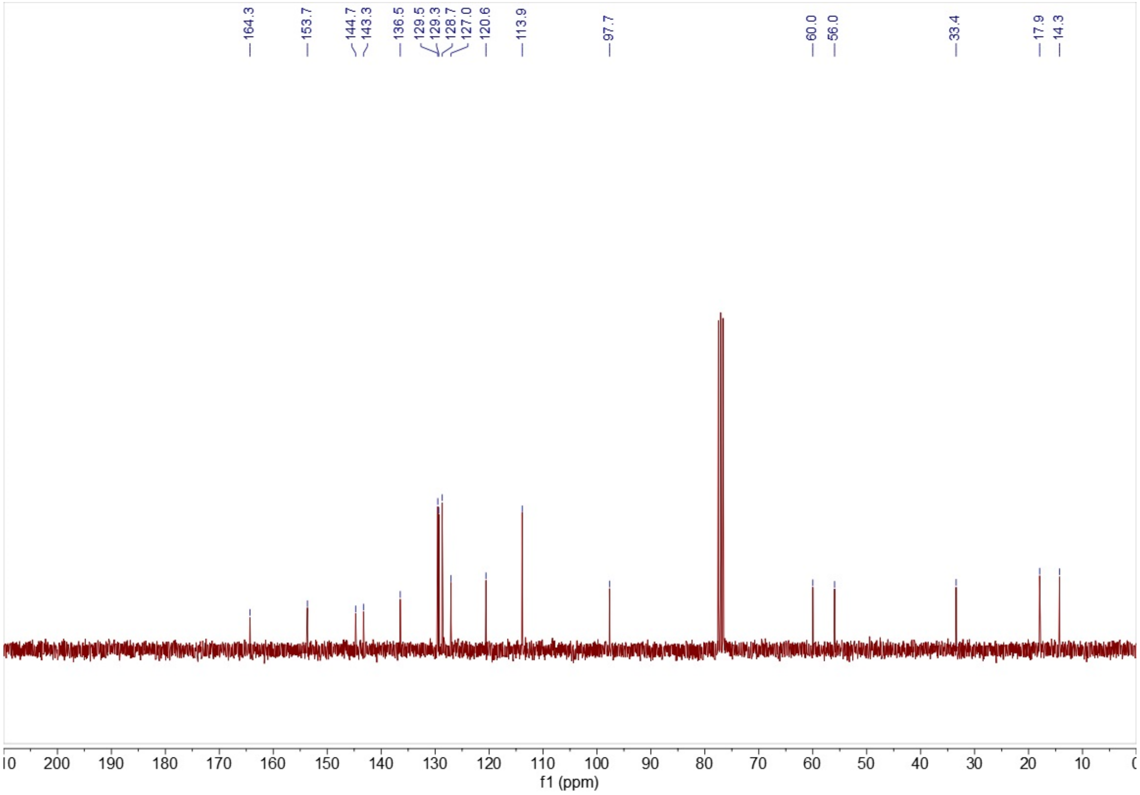

(*Z*)-Ethyl 2-(5-benzyl-2-cyclohexyl-4-phenyl-4*H*-1,3,4-oxadiazin-6(5*H*)-ylidene)acetate (3v)

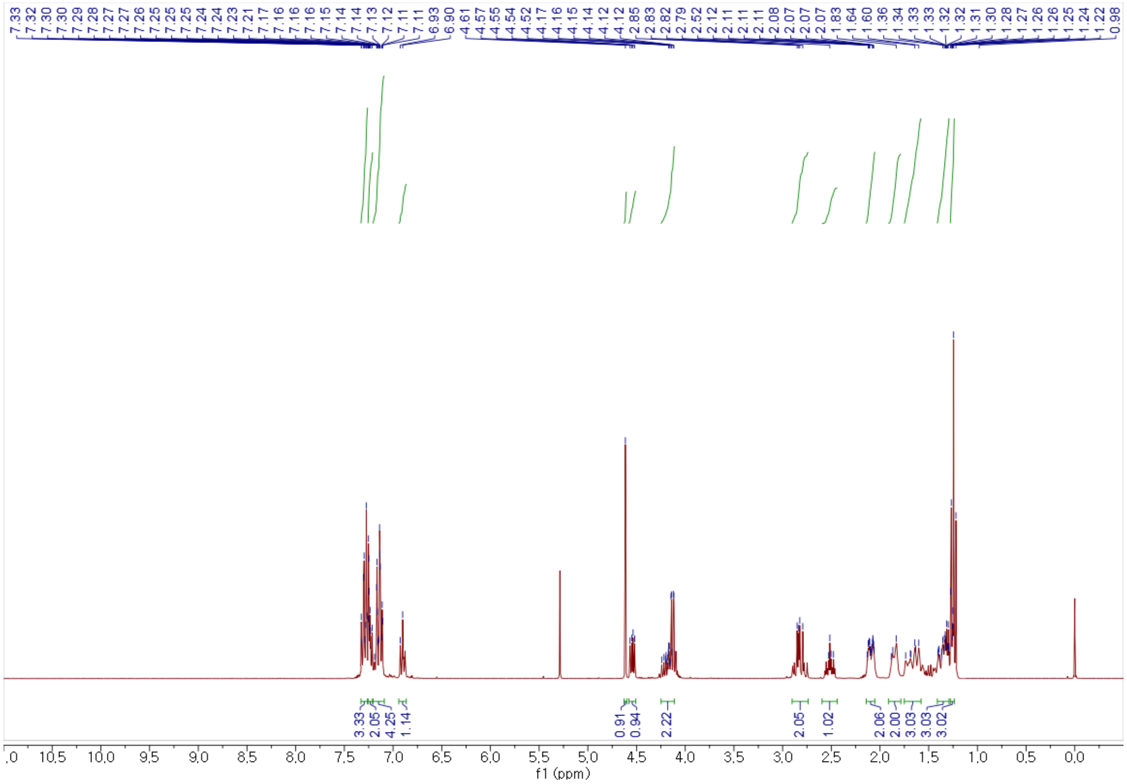

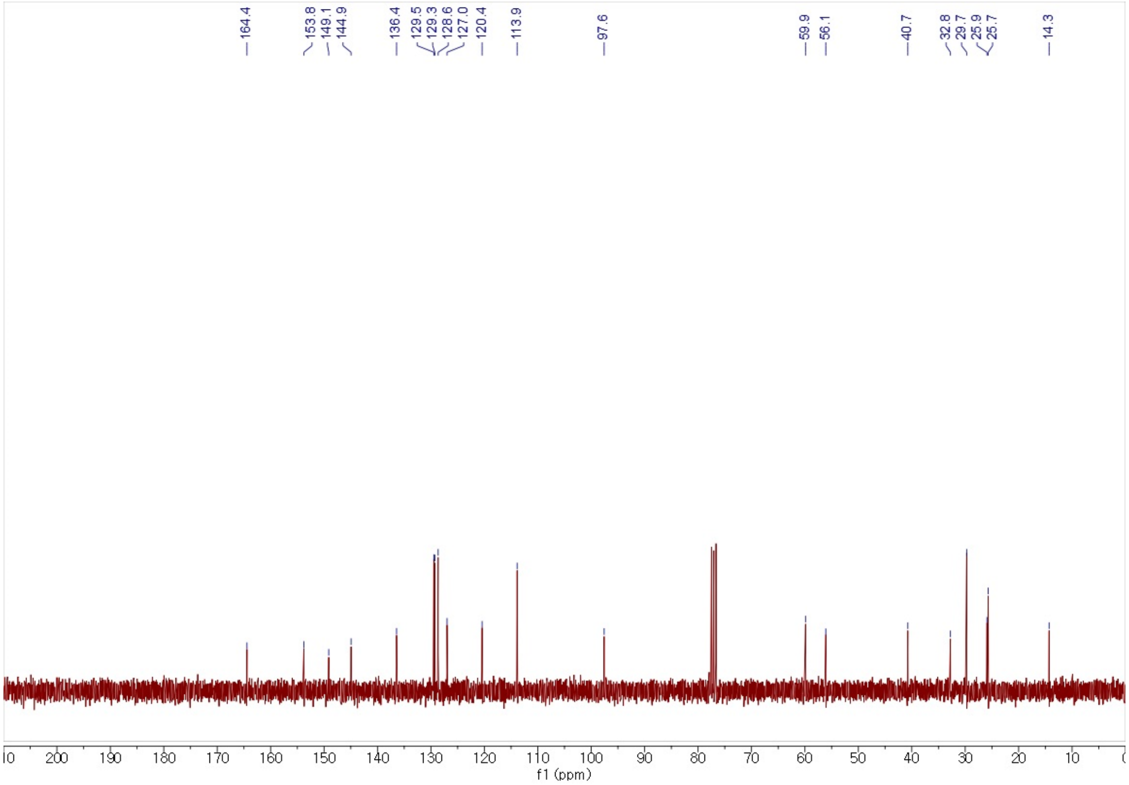

(*Z*)-Ethyl 2-(5-benzyl-2-(4-bromophenyl)-4-(*m*-tolyl)-4*H*-1,3,4-oxadiazin-6(5*H*)-ylidene)acetate (3w)

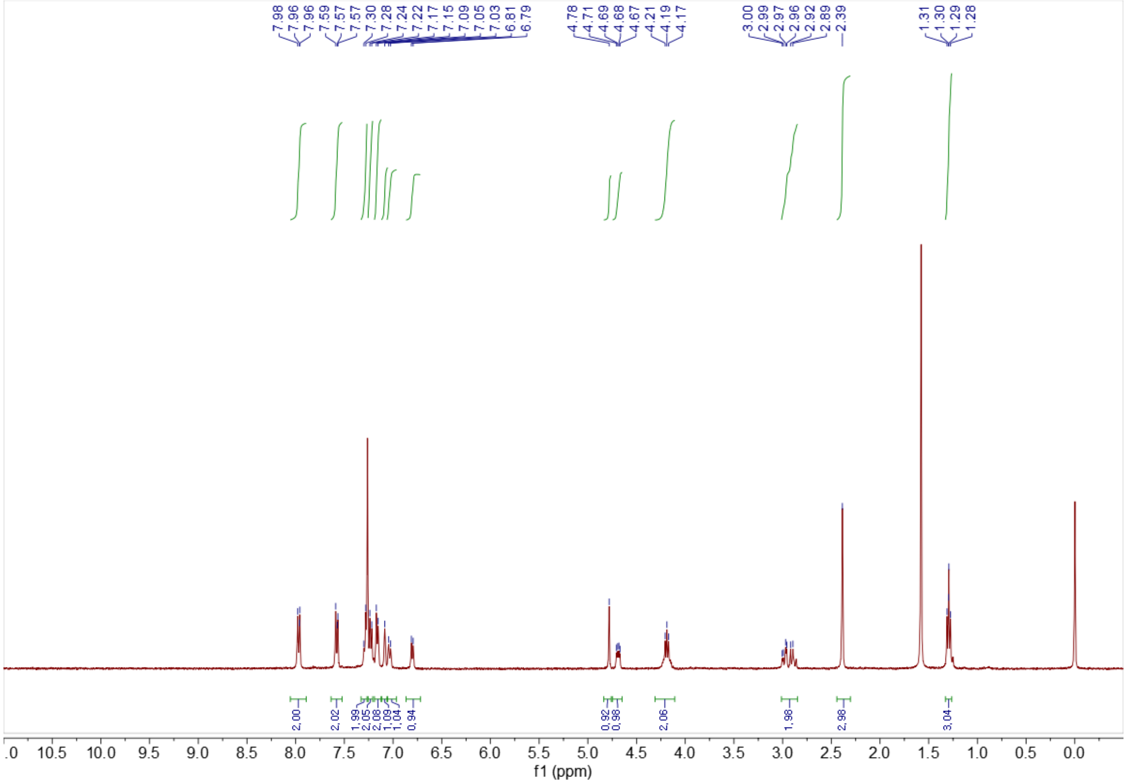

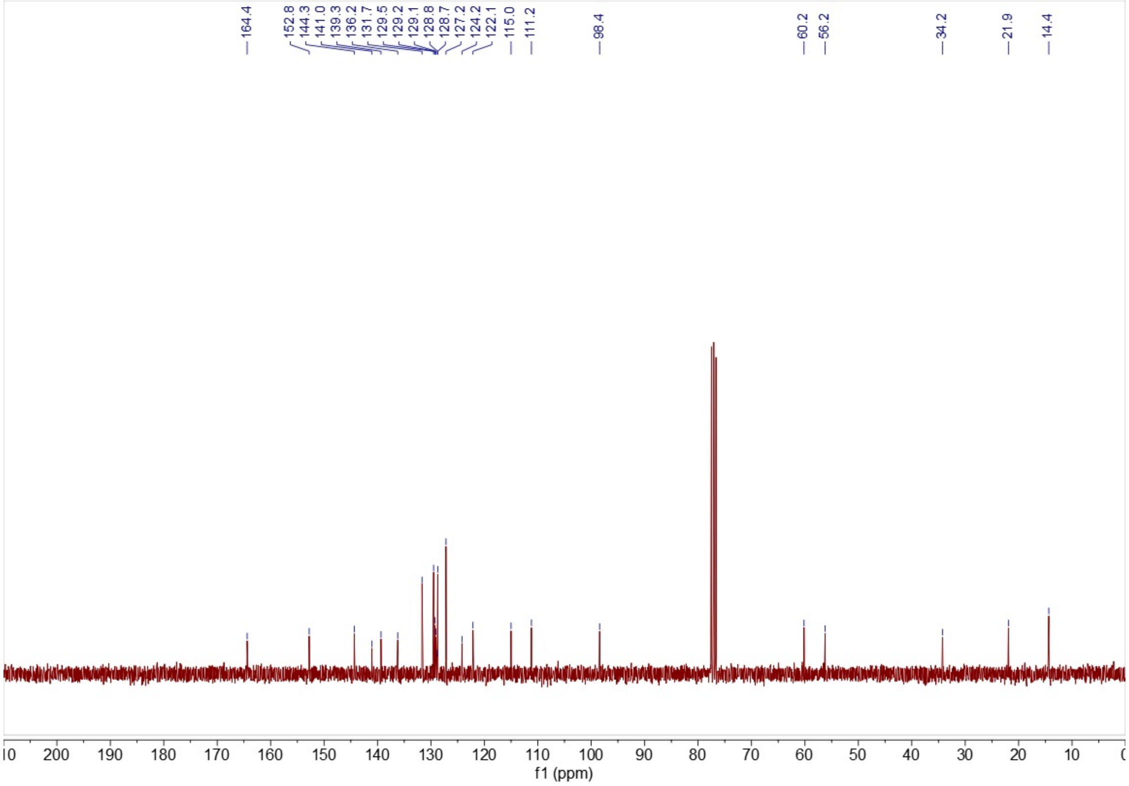

(*Z*)-Ethyl 2-(5-benzyl-4-(4-bromophenyl)-2-(4-methoxyphenyl)-4*H*-1,3,4-oxadiazin-6(5*H*)-ylidene)acetate (3x)

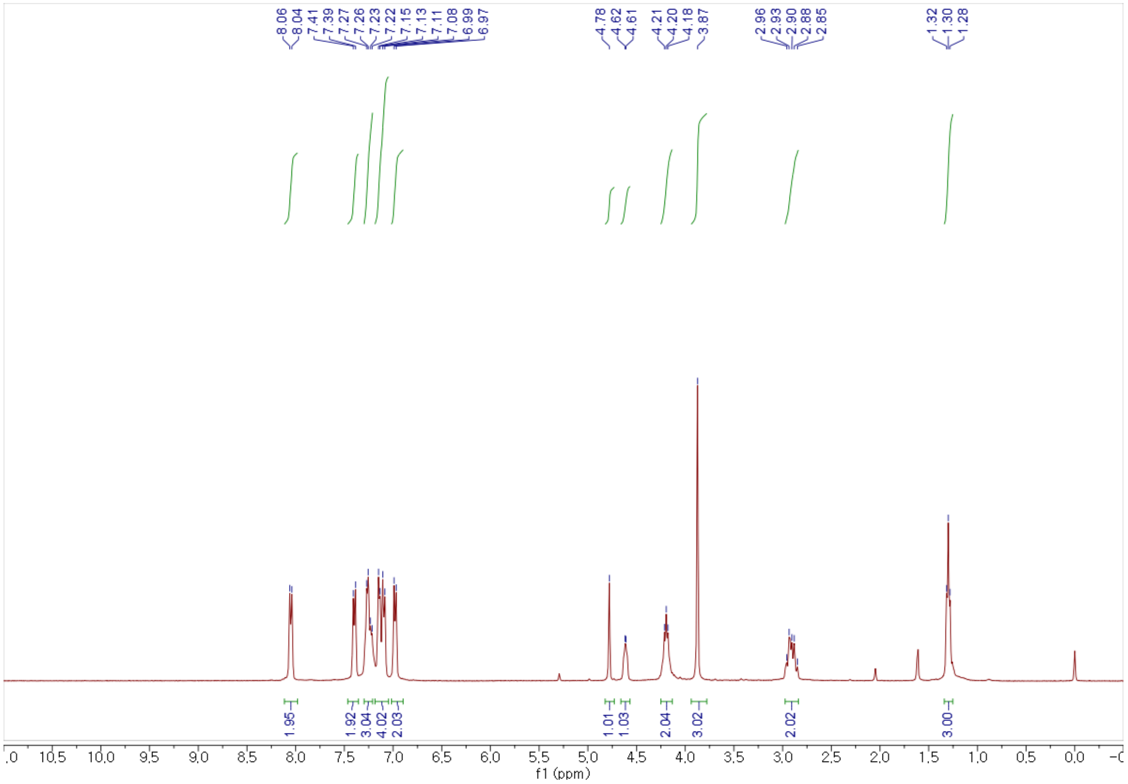

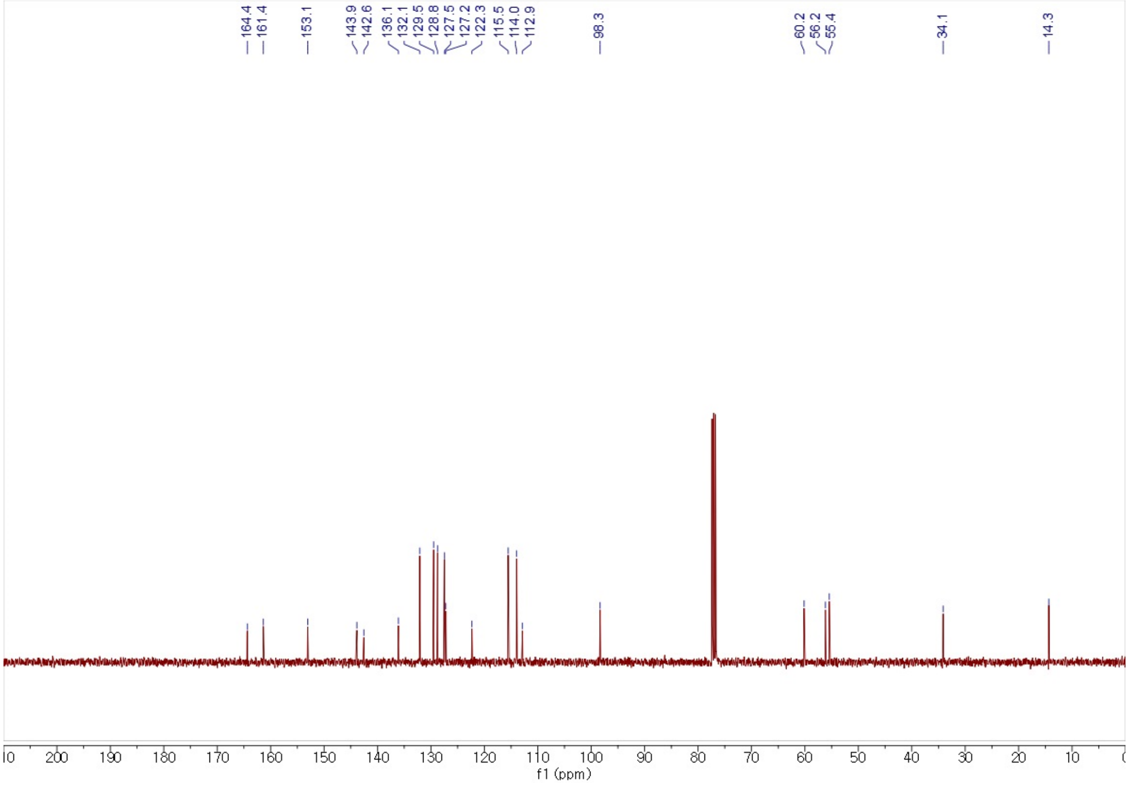

(*Z*)-Ethyl 2-(5-methyl-2,4-diphenyl-4*H*-1,3,4-oxadiazin-6(5*H*)-ylidene)acetate (3y)

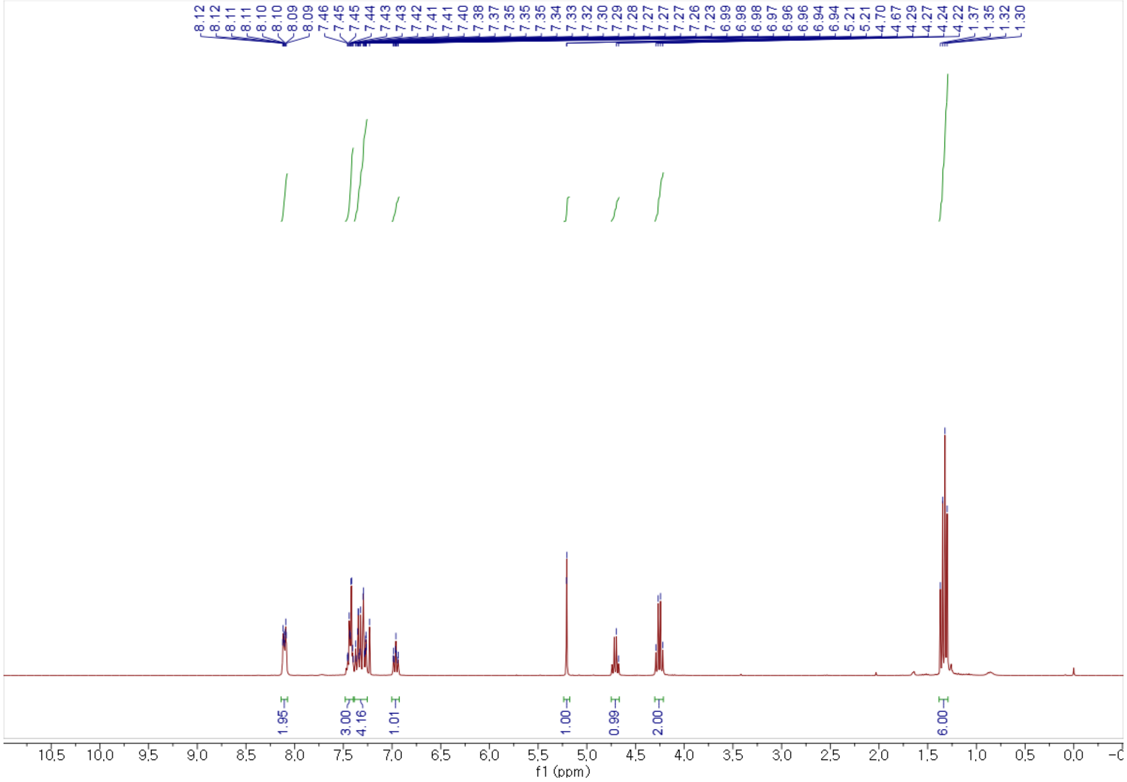

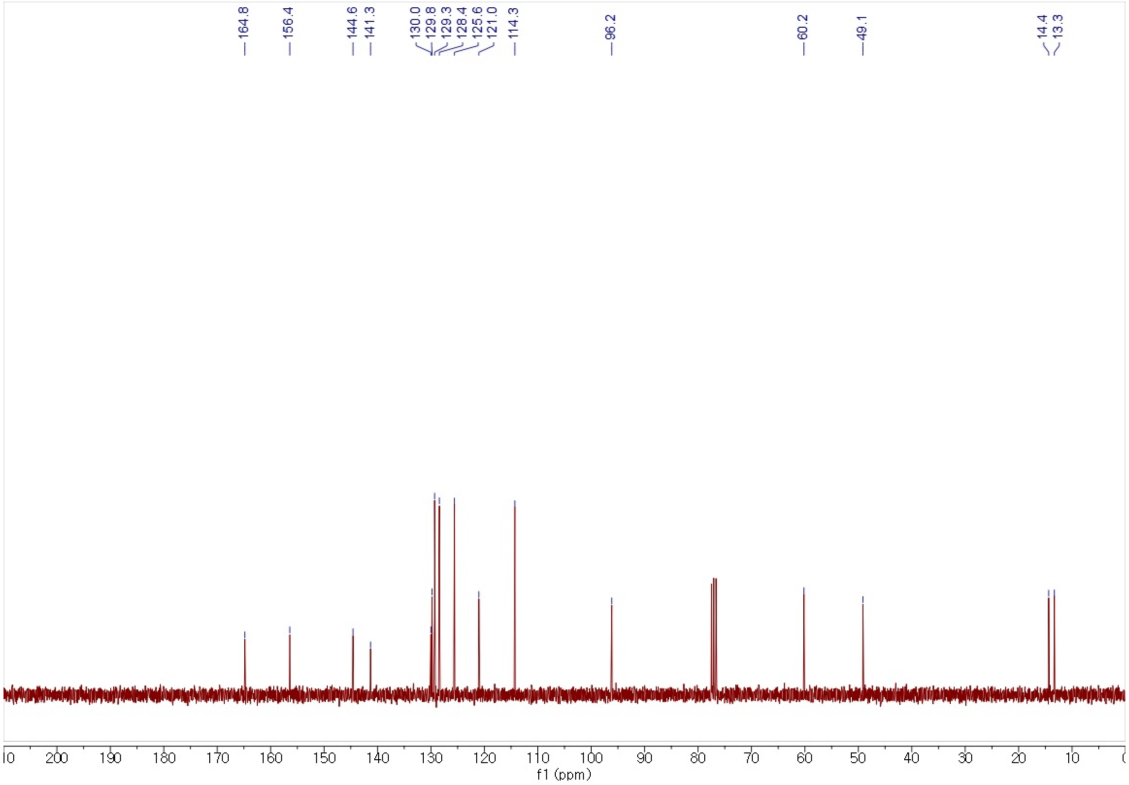

(*Z*)-Ethyl 2-(5-ethyl-2,4-diphenyl-4*H*-1,3,4-oxadiazin-6(5*H*)-ylidene)acetate (3z)

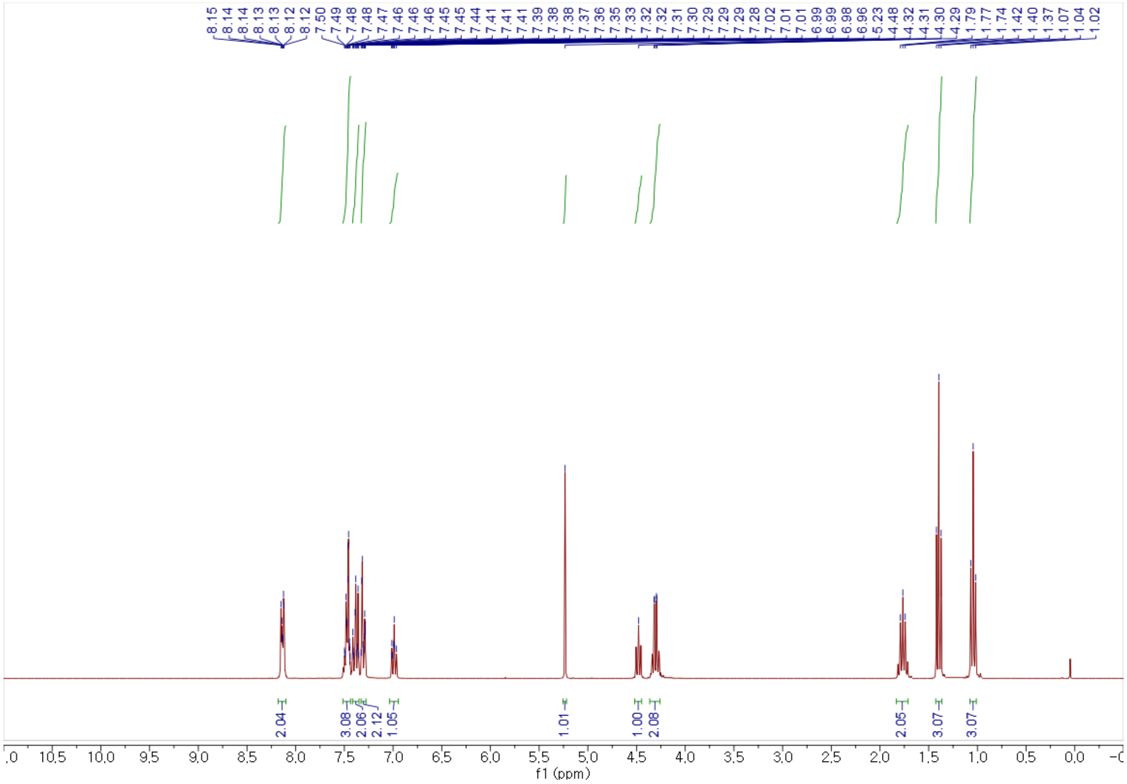

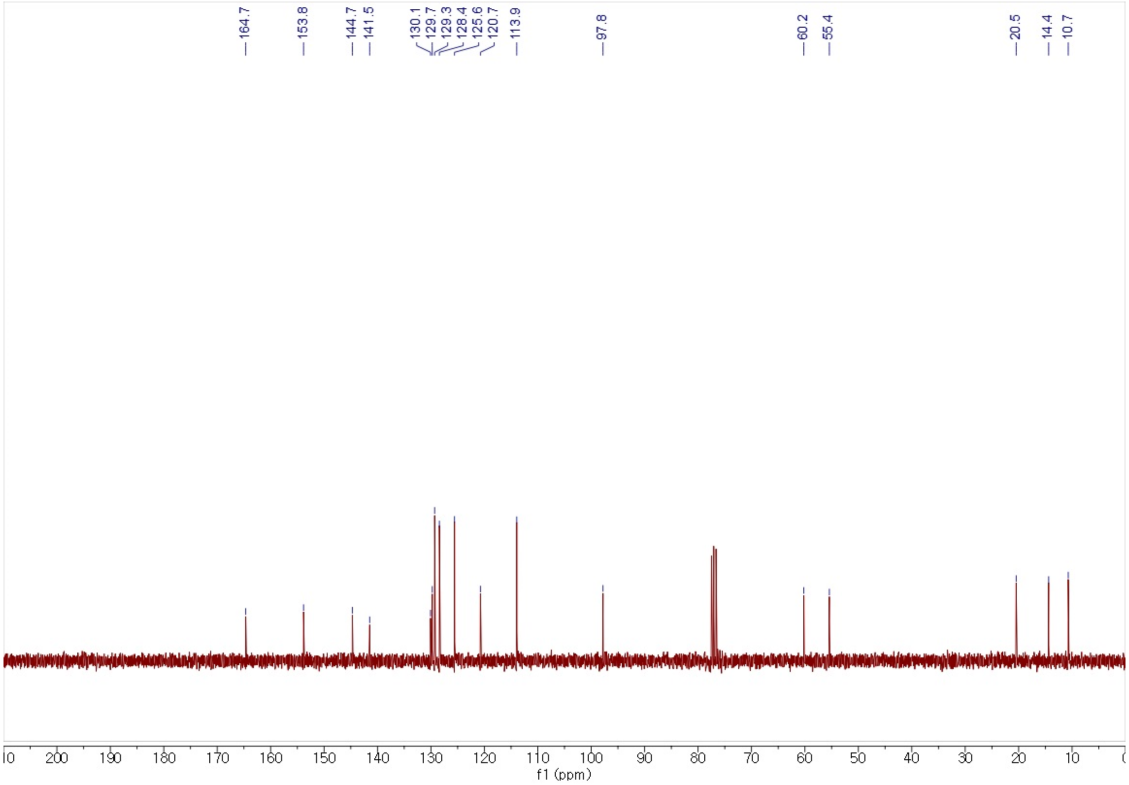

(*Z*)-Ethyl 2-(5-isobutyl-2,4-diphenyl-4*H*-1,3,4-oxadiazin-6(5*H*)-ylidene)acetate (3aa)

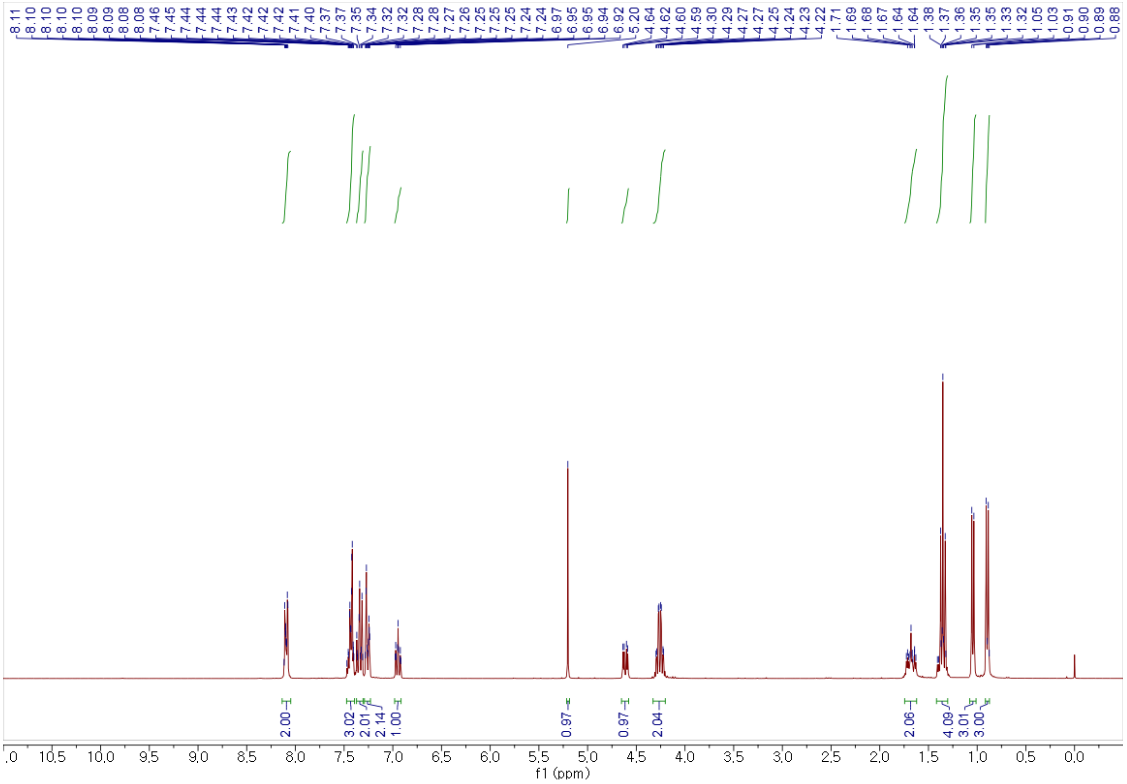

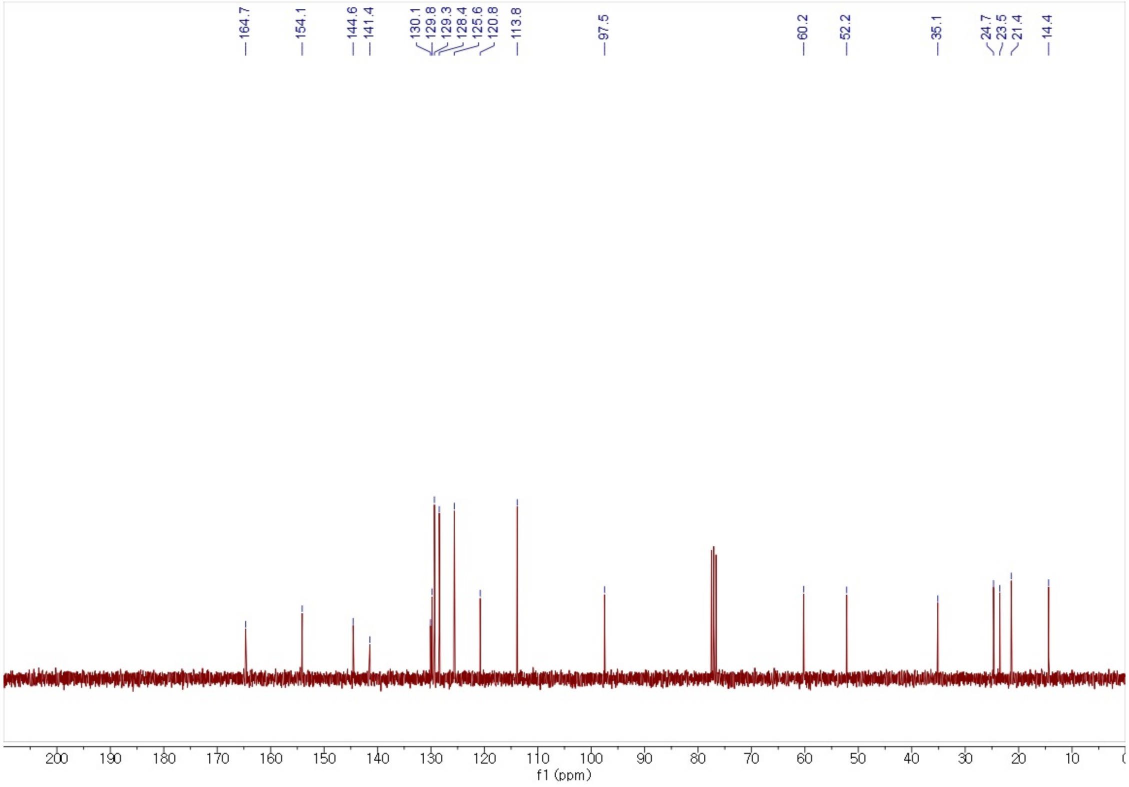

(*Z*)-Ethyl 2-(2,4,5-triphenyl-4*H*-1,3,4-oxadiazin-6(5*H*)-ylidene)acetate (3ab)

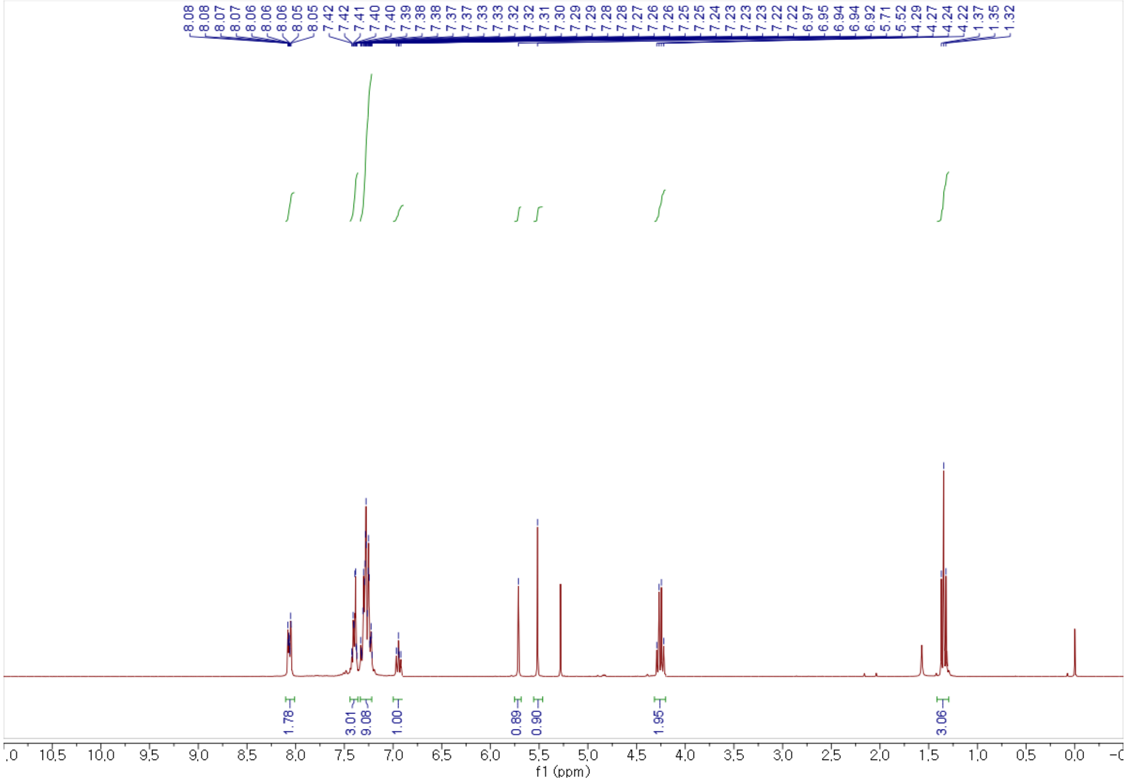

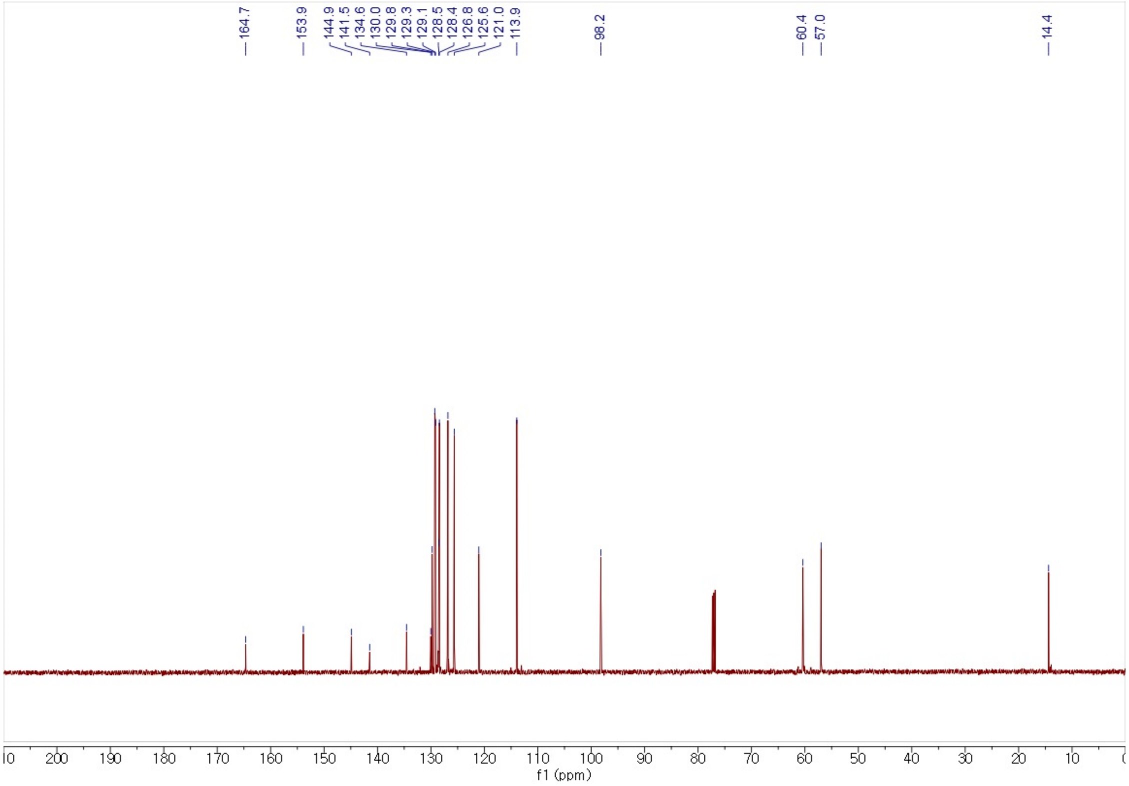

(*Z*)-Benzyl 2-(5-benzyl-2,4-diphenyl-4*H*-1,3,4-oxadiazin-6(5*H*)-ylidene)acetate (3ac)

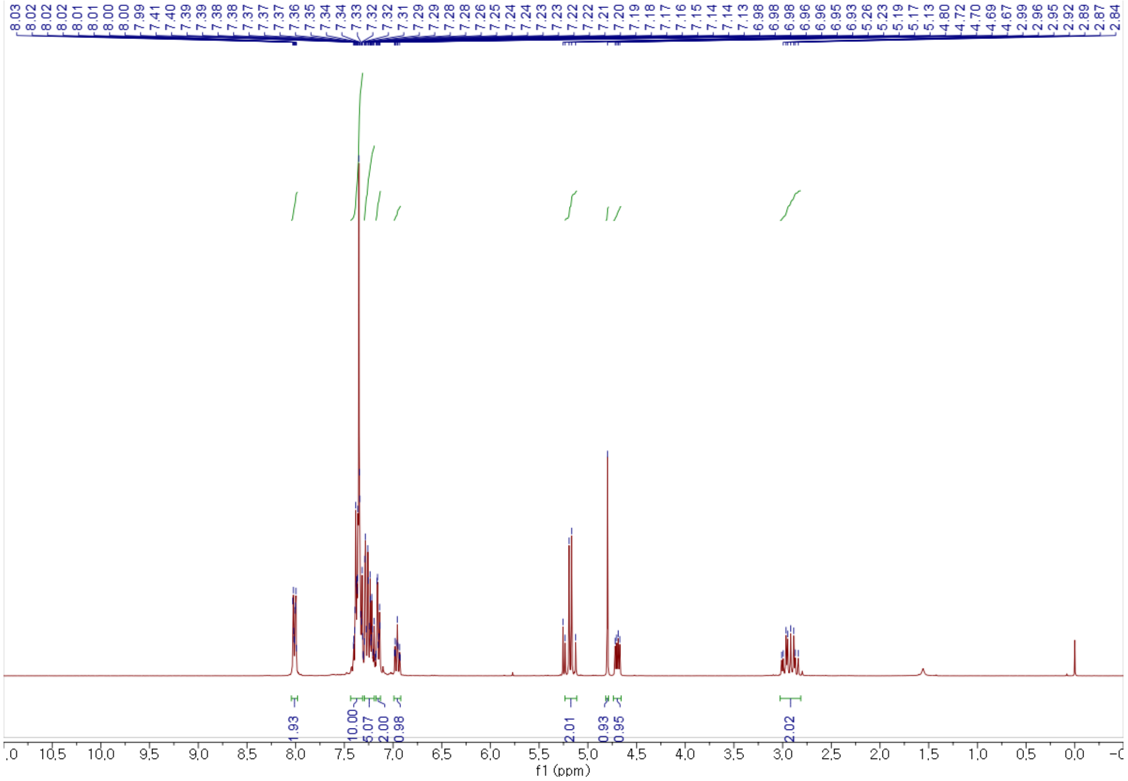

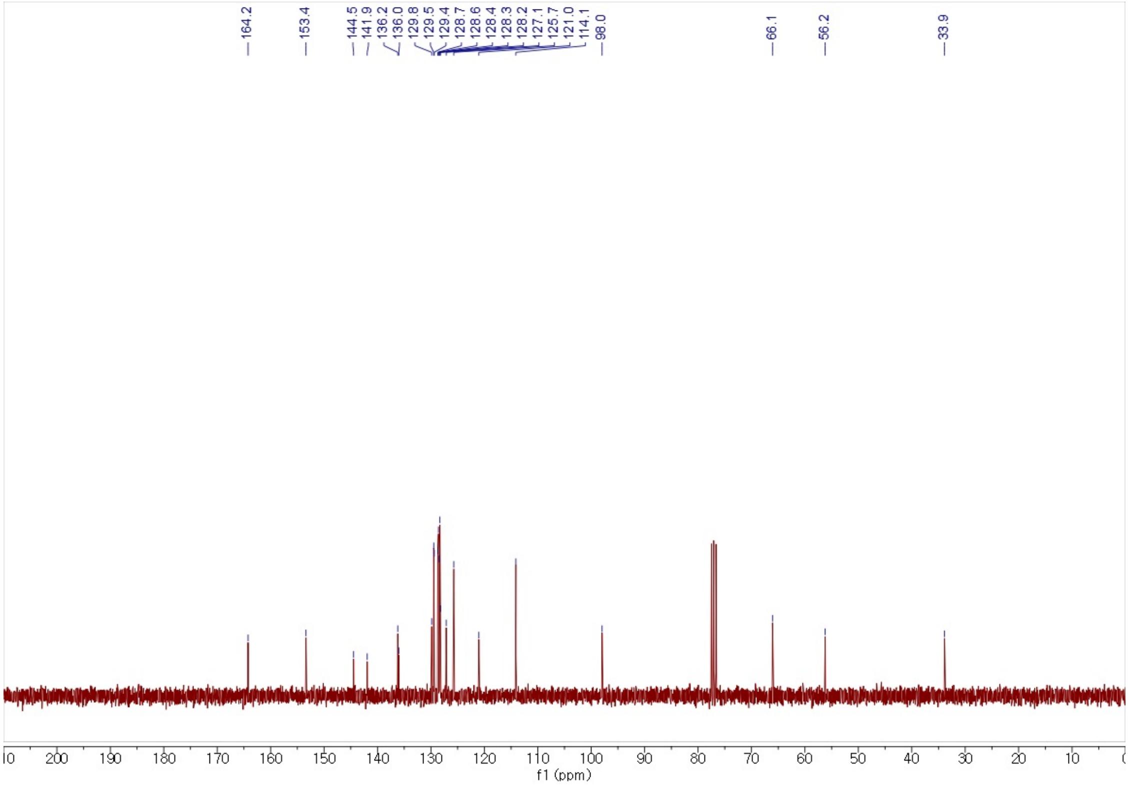

(*Z*)-Methyl 2-(5-ethyl-2,4-diphenyl-4*H*-1,3,4-oxadiazin-6(5*H*)-ylidene)acetate (3ad)

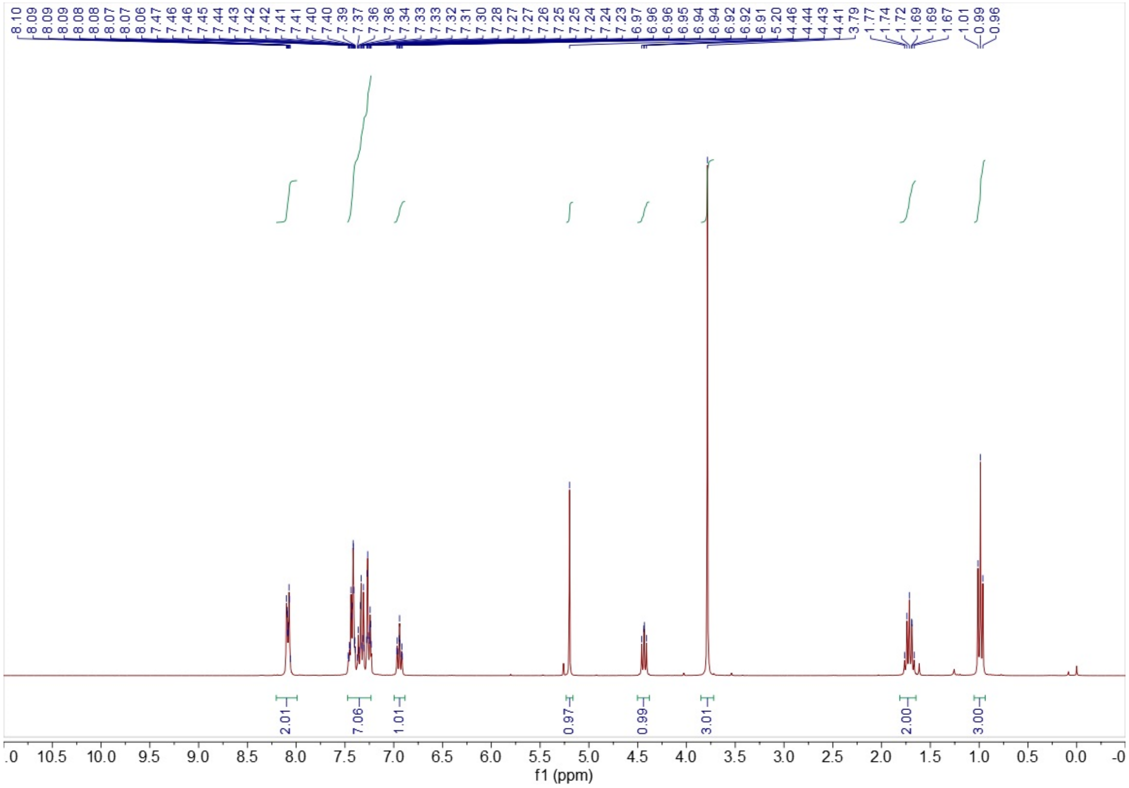

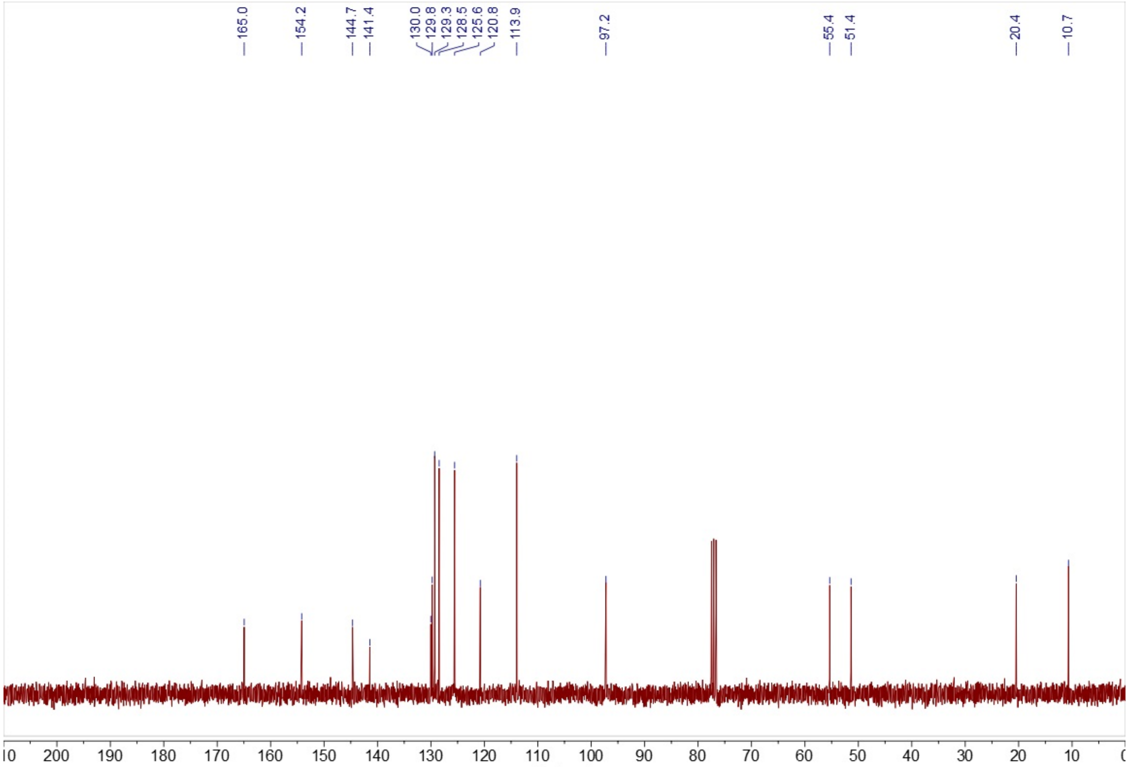

Supplement: Supplementary file 1 [file molecules-28-03815-s001.zip › molecules-2345055-supplementary.pdf]
